# A broad assessment of forty-one skin phenotypes reveals complex dimensions of skin ageing

**DOI:** 10.1186/s40101-024-00383-2

**Published:** 2025-02-08

**Authors:** Jun Yan Ng, Qi Yi Ambrose Wong, Jun Jie Lim, Dingyu Cen, Jia Yi Karen Wong, Yi Ying Eliza Lim, Yang Yie Sio, Kavita Reginald, Yee-How Say, Fook Tim Chew

**Affiliations:** 1 Department of Biological Sciences, Faculty of Science, National University of Singapore, Singapore, 117543 Singapore; 2 Department of Biological Sciences, School of Medicine and Life Sciences, Sunway University, Petaling Jaya, Malaysia; 3 Department of Biomedical Science, Faculty of Science, Universiti Tunku Abdul Rahman (UTAR), Kampar, Malaysia; 4 Allergy and Molecular Immunology Laboratory, Lee Hiok Kwee Functional Genomics Laboratories, Block S2, Level 5, 14 Science Drive 4, Lower Kent Ridge Road, Singapore, 117543 Singapore

**Keywords:** Skin aging, Skin ageing, Broad assessment, Cross-sectional study, Wrinkling, Sagging, Principal Component Analysis, Chinese, Singapore/Malaysia Cross-sectional Genetics Epidemiology Study, SMCGES

## Abstract

**Background:**

Skin ageing takes on many different forms. Despite this diversity in skin ageing phenotypes, literature published to date is limited in scope, as many research studies either focus on one single phenotype or just a few specific phenotypes. Presently, phenotypes such as wrinkles, pigment spots, and photo-ageing are receiving most of the research attention. We therefore wonder whether the current discourse on skin ageing places a disproportionate amount of focus on a few selected phenotypes, leaving other skin ageing phenotypes underexplored.

**Methods:**

In this cross-sectional study, we performed a broad assessment of forty-one signs of skin ageing and characterised the phenotypes that constituted key components of skin ageing. We also explored the interrelationship among forty-one skin ageing phenotypes using Spearman’s Correlation and Principal Component Analysis.

**Results:**

We analysed our study population, which is composed of 3281 ethnic Chinese participants from the Singapore/Malaysia Cross-sectional Genetics Epidemiology Study (SMCGES). The first ten principal components cumulatively explain 46.88% of the variance of skin ageing phenotypes in our study population. We discovered that the commonly discussed forms of skin ageing (i.e., wrinkles, pigmentation, and photo-ageing) only accounted for a small portion (24.39%) of the variance of all skin ageing phenotypes in our study population. Telangiectasia, a poor lip fullness, a lighter skin colour, xerosis, ephelides (freckles), ptosis of eyelids (droopy eyelids), eyebags, and a low eyebrow positioning were other key components of skin ageing, accounting for a further 22.49% of the variance of skin ageing phenotypes in our study population. We found that each of these ten skin ageing phenotypes characterises a key and important aspect of skin ageing. In this broad assessment of skin ageing, we first described the prevalence of forty-one signs of skin ageing and then characterised in detail both the prevalence and severity distribution of ten key skin ageing phenotypes.

**Conclusions:**

We presented clear evidence that skin ageing is much more than just wrinkles, pigmentation and photo-ageing. The addition of telangiectasia, poor lip fullness, a lighter skin colour, xerosis, ephelides, ptosis of eyelids, eyebags, and a low eyebrow positioning added more dimensions to skin ageing phenotype presentations.

**Supplementary Information:**

The online version contains supplementary material available at 10.1186/s40101-024-00383-2.

## Introduction

Skin ageing refers to any change to the skin that occurs due to ageing [1]. Skin ageing is driven by intrinsic factors (e.g., genetic factors, chronological influences) [2] and extrinsic factors (environmental sources) [3]. We previously examined seven key risk factors (age, sex, ethnicity, nutrition, smoking, air pollution, and exposure to ultraviolet (UV) light) and other important risk factors (stress and sleep) in a meta-analysis and systematic review [1]. The study of skin ageing in its entirety is challenging and complex.

The skin does not age uniformly. Instead, we identified at least fifty-six different skin ageing phenotypes [2]. Despite the diversity of these phenotypes, existing literature is limited in both range and scope. Most publications focused on one or a few phenotypes at a time (i.e., a limited range) [2], with wrinkles, pigment spots, and photo-ageing receiving the most interest (i.e., a limited scope) [4–6]. Therefore, we question whether current knowledge on skin ageing is disproportionately focused on a few selected phenotypes.

The objective of our study is a broad assessment of skin ageing. This is a cross-sectional study in which we examined the prevalence of skin ageing in 3281 ethnic Chinese from the Singapore/Malaysia Cross-sectional Genetics Epidemiology Study (SMCGES). Using Spearman Correlation and Principal Component Analysis (PCA) methods, we characterise how skin ageing phenotypes with different morphologies were interrelated in our study population.

## Materials and methods

### Participant recruitment and phenotype evaluations

The SMCGES is an ongoing genetics and epidemiology collection conducted in Singapore and Malaysia universities previously described [7–14]. To assess skin ageing, a volunteer sample of subjects was taken from Singapore (National University of Singapore) and Malaysia (Sunway University) universities. Skin ageing assessment volunteers were recruited from 2011 to 2023 via walk-in. While all volunteers aged between 18 and 80 and could read and write in English were eligible to participate in the study, those who attended our study venue and participated in the study were aged 18 to 73. Through an investigator-administered questionnaire, all participants provided sociodemographic data (Table 1) and self-reported their skin ageing phenotypes (Additional File 1). Sex, race, total monthly family income per capita, and perceived stress were self-reported. Total monthly family income per capita falls into one of four groups: low, moderate, high, and very high. In Singapore, low income refers to < $2000 Singapore dollars (SGD), moderate income refers to $2000–3999 SGD, high income refers to $4000–5999 SGD, and very high income refers to > $6000 SGD. In Malaysia, low income refers to < 3000 Malaysian ringgit (RM), middle income refers to RM3000-5999, high income refers to RM6000-12999, and very high income refers to > RM13000.

**Table 1:** Demographics of Singapore and Malaysia participants recruited from the SMCGES for the current assessment

Demographics	All respondents (*N* = 3876)^a^	Chinese respondents (*n* = 3281)^a^
Age, mean (SD), y	25.7 (6.9)	25.7 (6.9)
Height, mean (SD), cm	165.0 (8.7)	165.2 (8.4)
Weight, mean (SD), kg	60.6 (13.3)	59.8 (12.7)
BMI, mean (SD), kg/m^2^	22.2 (4.9)	21.8 (3.7)
Sex^b^		
Male	1352 (34.9)	1146 (34.9)
Female	2524 (65.1)	2135 (65.1)
Race^c^		
Chinese	3281 (84.6)	3281 (100)
Malay	194 (5.0)	0 (0)
Indian	226 (5.8)	0 (0)
Others^d^	175 (4.5)	0 (0)
Total monthly family income per capita^c^		
Low	482 (12.4)	377 (11.5)
Moderate	922 (23.8)	760 (23.2)
High	965 (24.9)	825 (25.1)
Very high	1501 (38.7)	1313 (40.0)
Missing/Invalid^e^	6 (0.2)	6 (0.2)
Housing^c^		
Flat	1819 (46.9)	1621 (49.4)
Condominium/Private Apartment	996 (25.7)	795 (24.2)
Landed Property	1053 (27.2)	857 (26.1)
Missing/Invalid^e^	8 (0.2)	8 (0.2)
Perceived Stress (measured using the PSS)^c^		
Low	Low	Low
Moderate	Moderate	Moderate
High	High	High
Missing/Invalid^e^	Missing/Invalid^e^	Missing/Invalid^e^

*Abbreviations*: *SMCGES* Singapore/Malaysia Cross-sectional Genetics Epidemiology Study, *SD* standard deviation, *y* years, *cm* centimetres, *kg* kilograms, *BMI* body mass index, *kg/m*^*2*^ kilograms per square metre, *PSS* perceived stress scale

^a^Data are presented as number (percentage) of study participants. The values after ± are standard deviation values

^b^Sex was self-reported

^c^Percentages may not total 100 due to rounding

^d^The other category included Arabs, Bulgarian, Burmese, Canadian, Egyptian, Filipino, German, Indonesian, Iranian, Japanese, Javanese, Khmer, Korean, Maldivian, Mauritian, Mexican, Mongolian, Nepali, Persian, Russian, Saudi, Siamese, Sri Lankan Moor, Sudanese, Trinidadian-British, Turkmen, Vietnamese, Whites, and Zimbabweans. All races were self-reported

^e^Missing/Invalid referred to responses that were either left blank or otherwise invalid

To account for potential bias, a randomised subset of self-reported phenotypes were compared against independent assessor gradings and was previously described [15].

To account for phenotype ascertainment bias, different skin ageing evaluation methods were compared in our earlier works [15–18].

In this study, we analysed a broad range of skin ageing phenotypes, broadly classified into wrinkles, sagging skin, dyspigmentation changes, and photo-ageing.

Skin ageing phenotypes were self-assessed using established tools: the Skin Ageing Atlas, validated photo-numeric scales, and photographic illustrations obtained from medical books (Additional File 1).

### Identification of skin ageing phenotypes to use in our current assessment

We previously performed a literature review in which we identified at least fifty-six skin ageing phenotypes [2]. We sought to identify these phenotypes in our study population. Of the fifty-six phenotypes, six were excluded from our current assessment as the ascertainment of these phenotypes required specialised tools or in vitro protocols. They were ‘wrinkles detected by shaded lines on the face’, ‘facial pigmented spots detected by polarised light capturing the skin surface’, ‘facial pigmented spots detected by UV light capturing the inside of the epidermis’, ‘skin reflectance’, ‘skin type – skin sensitivity to the Sun’, and ‘different gene expression in young skin compared to aged skin’.

While ‘fine lines on the cheek’ and ‘coarse wrinkles on the cheek’ were reported as two distinct phenotypes in the literature [19], we collapsed them into ‘cheek folds’ as they appeared to be describing the spectrum of the same phenotype. Similarly, ‘superficial Crow’s Feet wrinkles’ and ‘deep Crow’s Feet wrinkles’, which were previously reported in the literature as two distinct phenotypes [20, 21], were also collapsed into ‘Crow’s Feet wrinkles’.

Whether the skin elicits a youthful look was measured in different ways in the literature, either as ‘facial skin that is more youthful than normal’ or ‘a younger perceived age’. As both descriptors appeared similar, we collapsed them into ‘age perception’. Similarly, we collapsed ‘Fitzpatrick skin type’ and ‘perceived skin colour’ into ‘skin darkness’.

Five skin ageing phenotypes (‘senile purpura’, ‘basal cell carcinoma’, ‘melanoma’, ‘non-melanoma skin cancer’, and ‘squamous cell carcinoma’) were absent in our current study population.

### Evaluating stress using the Perceived Stress Scale (PSS)

Perceived stress was studied using the Perceived Stress Scale (PSS) [22]. This scale has been validated for use in Chinese populations [23]. In the PSS, participants answered ten questions on their perceived stress levels and were scored from 0 to 40. Perceived stress levels were graded using the instructions of this classic stress assessment instrument. 0 to 13: low stress, 14 to 26: moderate stress, 27 to 40: high stress.

### Statistical analysis

All analyses were performed using Version 25 of the IBM Statistical Package for Social Scientists (SPSS/PC).

Two-tailed bivariate correlations for Spearman’s Rank Correlation (ρ), Pearson’s Correlation Coefficient (r), and point biserial correlation coefficient (r_pb_) were calculated. Qualitative interpretations of the correlation values follow the naming practices for the strength of correlation coefficients used in healthcare and related fields [24, 25] 0.00: no correlation, 0.01–0.20: weak, 0.21–0.50: fair, 0.51–0.70: moderate, 0.71–0.90: very strong, 0.91–1.00: perfect.

**Fig. 1:**
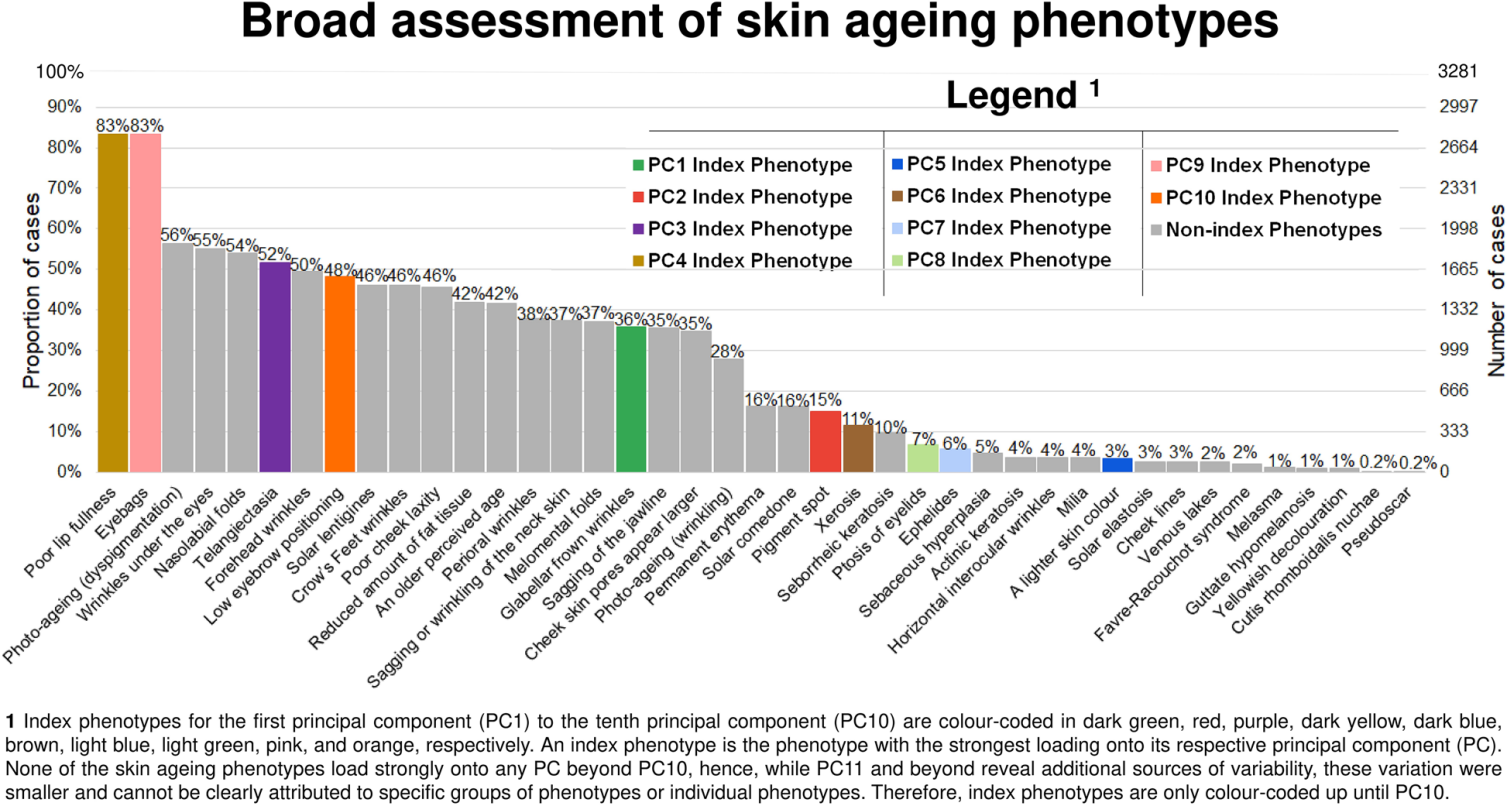
Prevalence of forty-one skin ageing phenotypes from ethnic Chinese participants from the Singapore/Malaysia Cross-sectional Genetics Epidemiology Study (SMCGES) (*n* = 3281)

Raw phenotype scores were standardised and normalised using z-score transformation and cube root transformation respectively. This ensured that each skin ageing phenotype contributed equally to the PCA. PCA was performed using the dimension reduction function in SPSS (direct oblimin with a delta of 0). Phenotypes which were evaluated as binary traits (i.e., presence or absence) were excluded from the PCA if there were 5% or fewer cases (Fig. 1), alike what was done by Eriksson et al*.*, 2010 [26]. In decreasing order of prevalence, the binary traits excluded from the PCA were ‘sebaceous hyperplasia’, ‘actinic keratosis’, ‘horizontal interocular wrinkles’, ‘milia’, ‘solar elastosis’, ‘cheek lines’, ‘venous lakes’, ‘Favre-Racouchot syndrome’, ‘melasma’, ‘guttate hypomelanosis’, ‘yellowish decolouration’, ‘cutis rhomboidalis nuchae’, and ‘pseudoscar’. ‘A lighter skin colour’ is retained as skin colour was not evaluated as a binary trait. Altogether, twenty-nine phenotypes were analysed by PCA.

Rotated factor loading scores were interpreted as follows – (i) Between −1.0 and −0.7: Phenotype(s) are very strongly correlated with the PC. (ii) Between −0.7 and −0.5: Phenotype(s) are strongly correlated with the PC. (iii) Between −0.5 and 0.5: Phenotype(s) are weakly correlated with the PC. (iv) Between 0.5 and 0.7: Phenotype(s) are strongly correlated with the PC. (v) Between 0.7 and 1.0: Phenotype(s) are very strongly correlated with the PC.

Additionally, the phenotype with the strongest correlation to the PC is termed as the index phenotype. Index phenotypes will be discussed in greater detail in Sect. 11.5 (Characterisation of index phenotypes from the first ten PCs).

Using the dimension reduction function in SPSS, we discovered that the first ten Principal Components (PCs) explained 46.88% of the phenotypic variance in our study population (Additional File 2).

PCA was carried out using matrix correlations on complete cases and using direct oblimin rotation. PCs with eigenvalues > 1 were retained based on the Kaiser criterion [27].

**Fig. 2:**
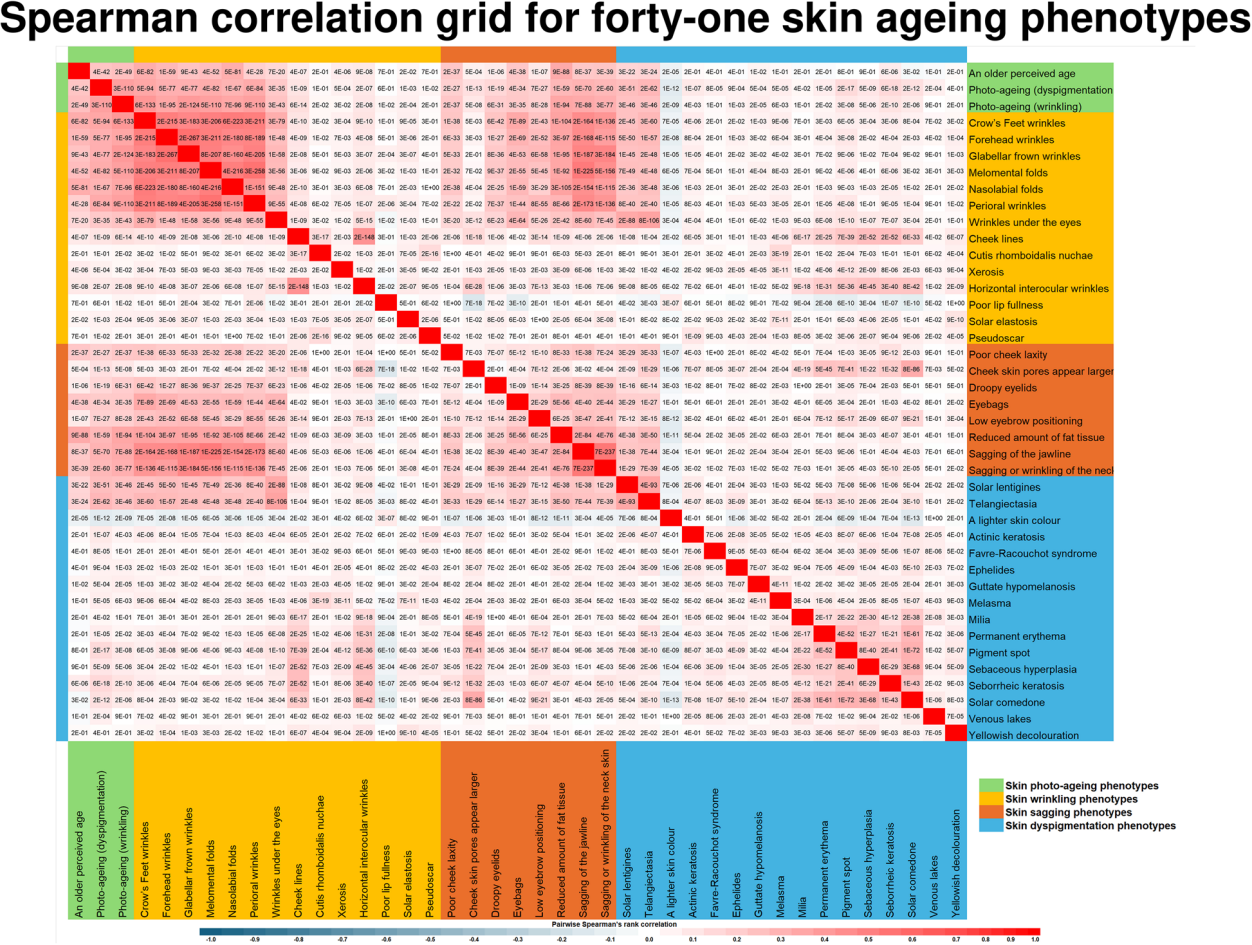
Pairwise Spearman correlation values among forty-one skin ageing phenotypes from the Singapore/Malaysia Cross-sectional Genetics Epidemiology Study (SMCGES) (*n* = 3281)

Correlation analysis among the phenotypes is a separate analysis from the PCA. Spearman Correlation analysis was computed using the bivariate correlation function in SPSS. For completeness in the presentation of data, the correlations among all 41 phenotypes were displayed, including those phenotypes excluded from the PCA as described earlier. Correlations were visualised on Microsoft Excel with two colour gradients (a blue gradient for correlation values from −1 to 0 and a red gradient for correlation values from 0 to +1) (Fig. 2). The values shown in each cell of the correlation table are Spearman Correlation p-values. For example, the p-value of the correlation between ‘an older perceived age’ and ‘photo-ageing (dyspigmentation)’ is 4 × 10^−42^. Scatter plots were drawn between age and PCA values using the ggplot2 package in R Studio Version 4.3.1.

Box-and-whisker plots were drawn between (a) sex and PCA values, (b) self-reported total monthly family income per capita and PCA values, and (c) PSS and PCA values. All box-and-whisker plots were drawn using the ggplot2 package in R Studio Version 4.3.1.

## Results

### Participant demographics

3876 participants completed the study. Participants who terminated the study midway (e.g., due to personal reasons) were classified as non-respondents and their data were not retained. To account for potential bias, the demographics of the volunteer respondents were compared against the non-respondents and was previously described [17].

As most of the participants were Chinese (*n* = 3281/3876, 84.6%), we selected only the Chinese population (*n* = 3281) for our final analysis. This ensured minimal ascertainment bias in our study and improved the statistical empowerment in our analyses.

**Fig. 3:**
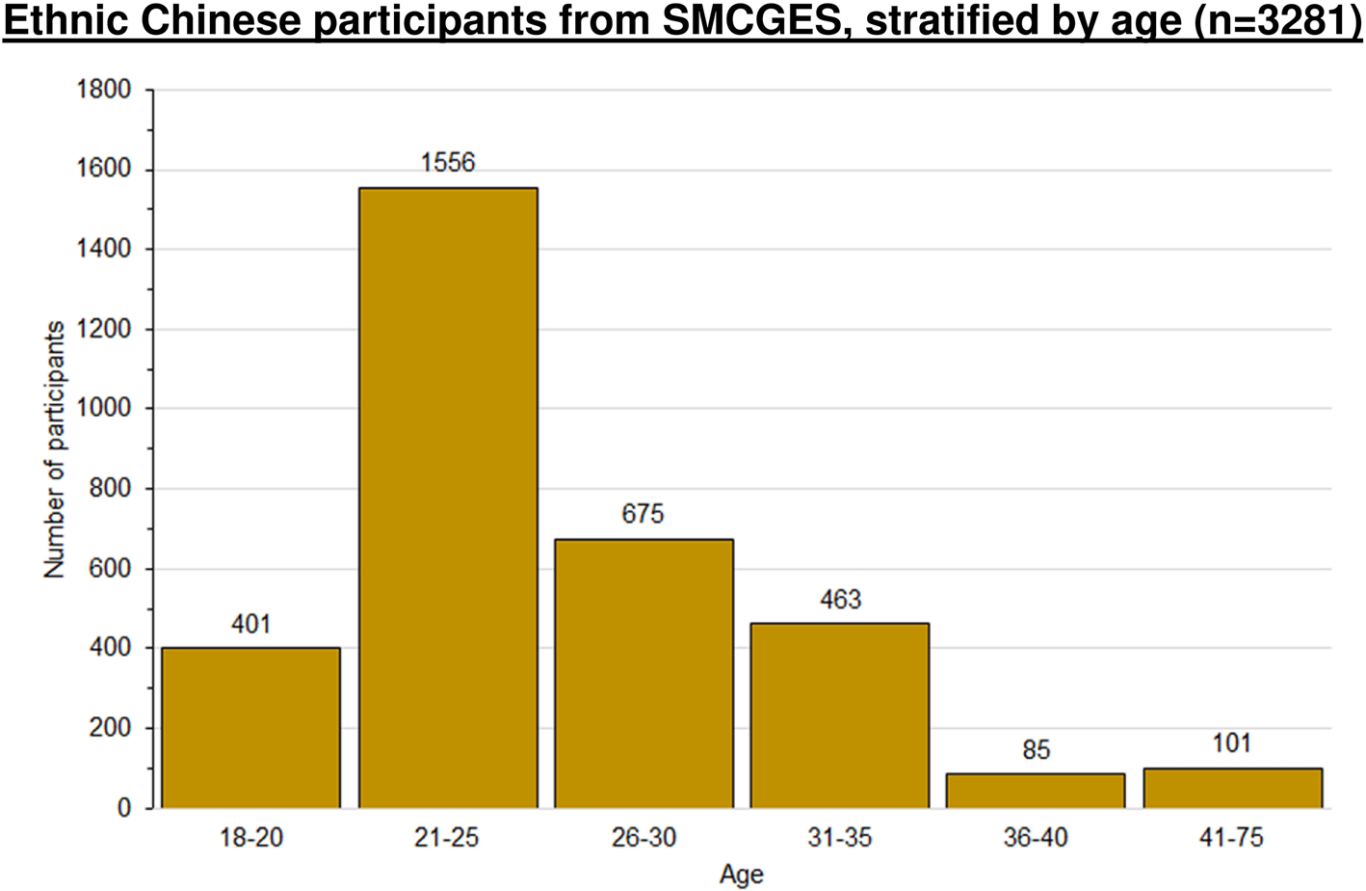
Age distribution of 3281 ethnic Chinese participants from the Singapore and Malaysia ethnic Chinese participants recruited from the Singapore/Malaysia Cross-sectional Genetics Epidemiology Study (SMCGES)

The final analysis consisted of more females (*n* = 2135/3281, 65.1%) than males. The mean age of the participants was 25.7 years old with a standard deviation (SD) of 6.9 years. Our study population consisted predominantly of young adults aged 21 to 30. The youngest participant was 18 years old, and the oldest participant was 73 years old (Table 1 and Fig. 3). Participants aged below 40 (i.e., younger participants) make up the bulk of our study population (*n* = 3180/3281, 96.9%). There were 101 participants aged 40 and above. In this work, we will also discuss the similarities and differences in prevalence rates between younger and older participants in Sect. 12.3 (Comparing younger participants with older participants) and in Additional Files 3–4.

### Prevalence of skin ageing in our study population

Overall, we identified forty-one different skin ageing phenotypes present in our study population in this current assessment (Fig. 1).

A poor lip fullness (*n* = 2735/3281, 83%) and eyebags (*n* = 2730/3281, 83%) were the most prevalent forms of skin ageing in our study population.

More than half of our study population (*n* = 1849/3281, 56%) had some extent of dyspigmentation, a key constituent of photo-ageing.

Facial wrinkles: wrinkles under the eyes (*n* = 1804/3281, 55%), nasolabial folds (*n* = 1770/3281, 54%), forehead wrinkles (*n* = 1625/3281, 50%), and Crow’s Feet wrinkles (*n* = 1514/3281, 46%) were present in nearly every one in two participants.

Telangiectasia (*n* = 1698/3281, 52%), low eyebrow positioning (*n* = 1585/3281, 48%), solar lentigines (*n* = 1515/3281, 46%), poor cheek laxity (*n* = 1496/3281, 46%), and reduced amount of fat tissue (*n* = 1377/3281, 42%) were present in almost half of our participants.

### Concordance among skin ageing phenotypes

We explored the relationship between different skin ageing phenotypes and found that many skin ageing phenotypes were intercorrelated (Fig. 2). The morphology of our phenotypes evaluated in the current assessment can be broadly classified into four categories: wrinkles, sagging skin, dyspigmentation changes, and photo-ageing. Phenotypes that exhibited characteristics of multiple categories (e.g., solar elastosis, Favre-Racouchot syndrome, and telangiectasia) (Additional File 1), were classified in Fig. 2 based on their predominant characteristic.

We found that morphology does not appear to influence the strength of the correlation among different skin ageing phenotypes.

The strongest correlation (Spearman’s Correlation ρ between 0.38–0.56) can be found among wrinkling and sagging phenotypes (Crow’s Feet wrinkles, forehead wrinkles, glabellar frown wrinkles, Melomental folds, nasolabial folds, perioral wrinkles, wrinkles under the eyes, sagging of the jawline, and sagging or wrinkling of the neck skin) (Fig. 2).

Skin wrinkling and sagging phenotypes (reduced amount of fat tissue, sagging of the jawline, and sagging or wrinkling of the neck skin) were also weak/fairly correlated with dyspigmentation changes (e.g., solar lentigines), and atrophic changes (e.g., telangiectasia) (Spearman’s Correlation ρ between 0.20–0.26) (Fig. 2).

We made three key observations. Firstly, skin ageing phenotypes do not occur independently from one another. While wrinkles and sagging skin were morphologically distinct phenotypes, wrinkly skin and saggy skin were concordant (Spearman’s Correlation ρ between 0.38–0.56). This suggested that wrinkles and sagging skin may share similar etiology (e.g., genetic origins).

Secondly, perceived age/skin youthfulness had been studied by several publications but the exact spectrum of phenotypes which contributed to an older perceived age have not been reported or discussed in detail. Here, using our repertoire of forty-one phenotypes, we found that an older perceived age was correlated with many diverse morphological changes to the skin. The primary influences were sagging skin (reduced amount of fat tissue, Spearman’s Correlation ρ: 0.34), wrinkles (Crow’s Feet wrinkles, Spearman’s Correlation ρ: 0.33; nasolabial folds, Spearman’s Correlation ρ: 0.32), atrophic changes (telangiectasia, Spearman’s Correlation ρ: 0.18), and dyspigmentation changes (solar lentigines, Spearman’s Correlation ρ: 0.17) (Fig. 2).

Lastly, all skin ageing phenotypes were positively correlated with one another except for ‘poor lip fullness’ and ‘a lighter skin colour’, which showed weak negative correlations. These will be discussed in detail in Sect. 12.2 (Negative correlations between ‘poor lip fullness’, ‘a lighter skin colour’, and other skin ageing phenotypes).

### PCA of skin ageing phenotypes

**Fig. 4:**
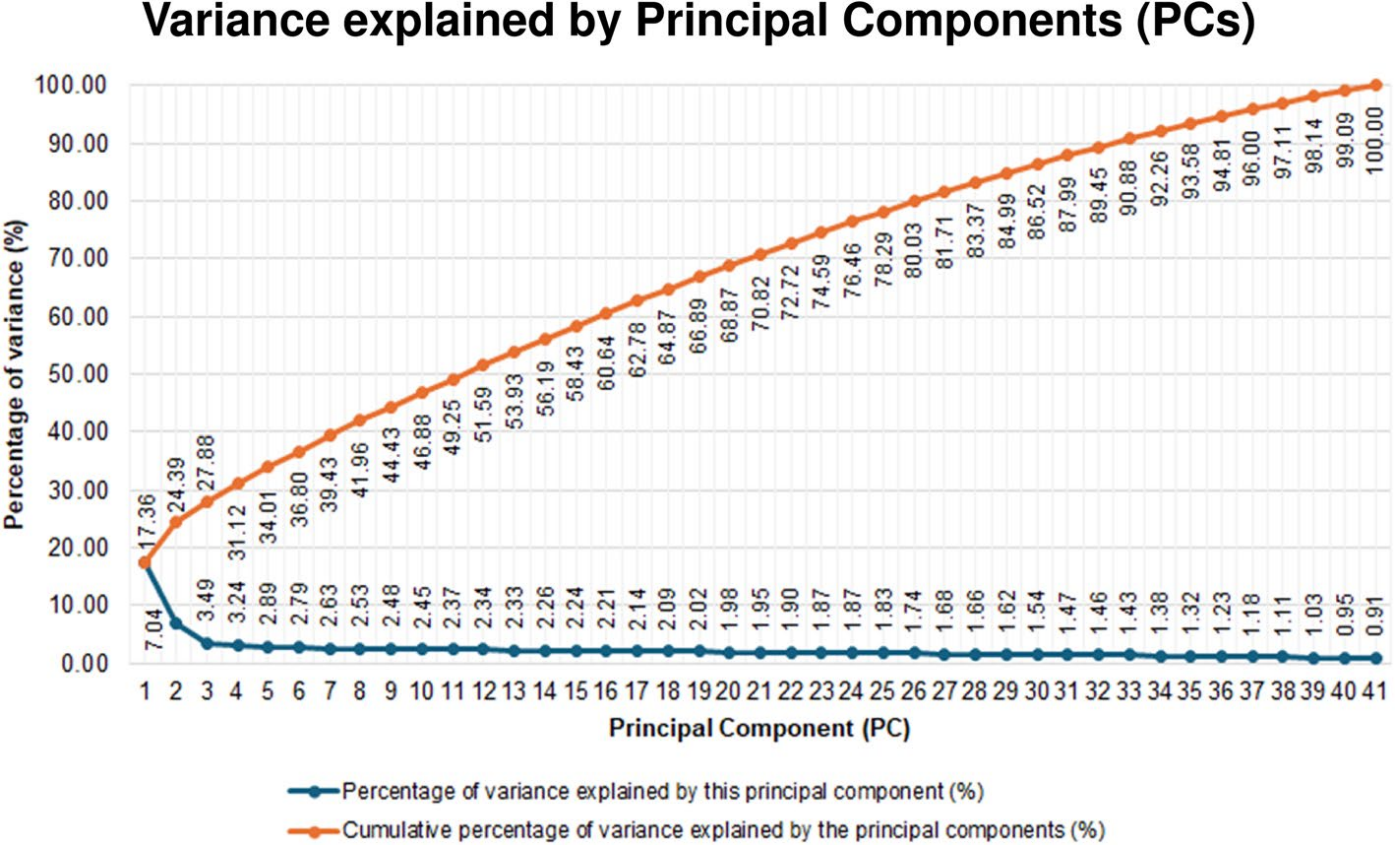
Principal Component Analysis (PCA) of forty-one skin ageing phenotypes

**Fig. 5:**
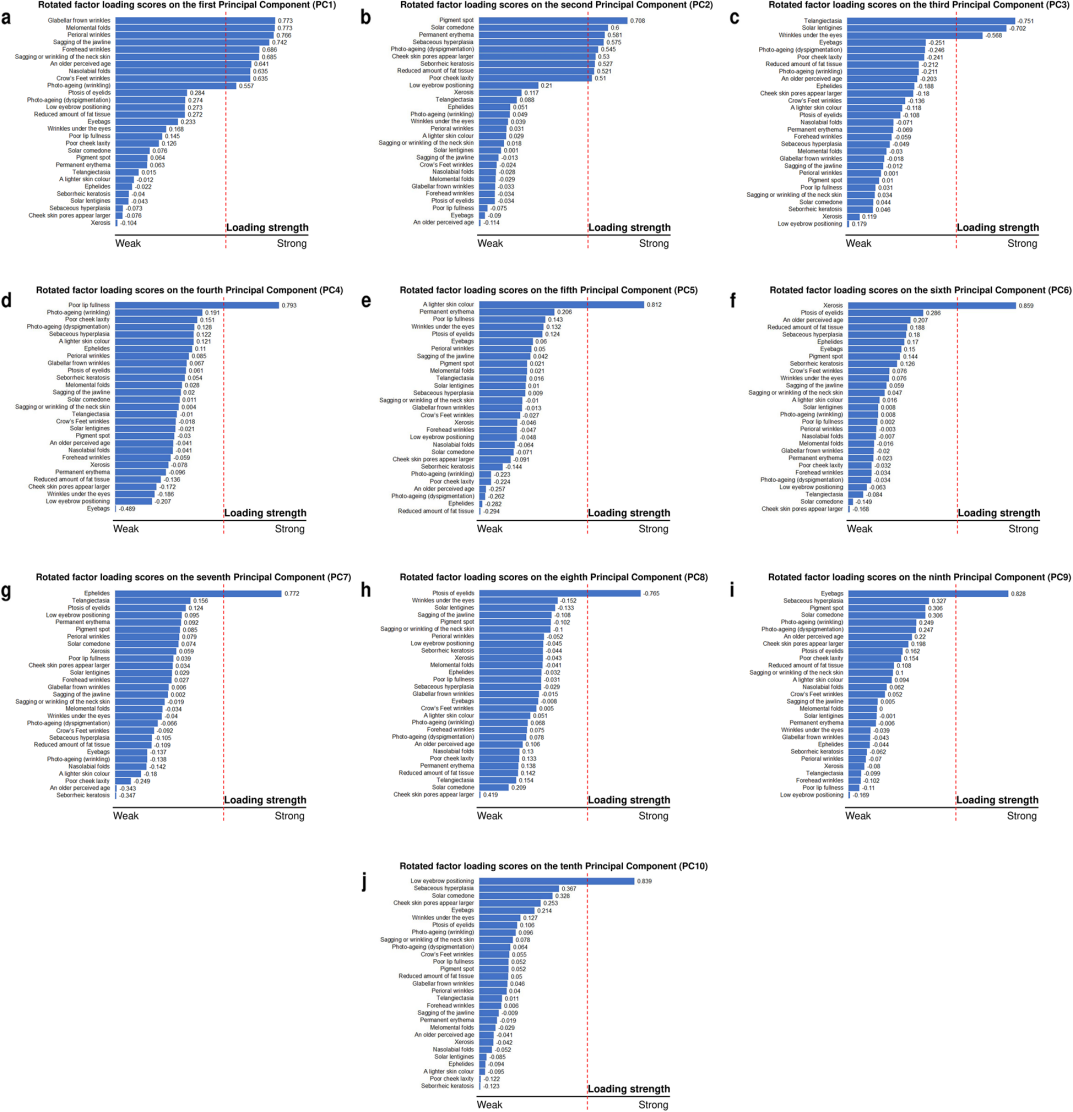
**a–j** Rotated factor loading scores of skin aging phenotypes in our study population onto the first ten principal components (PCs): **a** PC1, **b** PC2, **c** PC3, **d** PC4, **e** PC5, **f** PC6, **g** PC7, **h** PC8, **i** PC9, and **j** PC10. Binary phenotypes (i.e., skin ageing phenotypes assessed as either present or absent) with 5% or fewer cases were excluded from the PCA and do not have rotated factor loading scores. Rotated factor loading scores are reported in this figure as loading strengths. The vertical red dotted line refers to a loading strength with a magnitude of 0.50. Phenotypes with bars extending to the right of the vertical red dotted line have loading strengths with magnitudes exceeding 0.50 and are said to be strongly loaded onto the PC. Phenotypes with bars that end before the vertical red dotted line have loading strengths with magnitudes below 0.50 and are said to be weakly loaded onto the same PC

As multiple skin ageing phenotypes appear to be intercorrelated, we sought to characterise these relationships further through a PCA (Fig. 4). A phenotype is strongly loaded onto a given PC if the magnitude of the rotated factor loading score on that PC is above 0.50, as defined in Sect. 10.4 (Statistical Analysis). For example, as the rotated factor loading score of glabellar frown wrinkles (i.e., a skin ageing phenotype) on the first Principal Component (PC1) is +0.773, glabellar frown wrinkles are strongly correlated with PC1 (i.e., glabellar frown wrinkles are strongly loaded onto PC1) (Fig. 5a). In contrast, ptosis of eyelids has a rotated factor loading score of +0.284 on PC1. Therefore, ptosis of eyelids is only weakly correlated with PC1 (Fig. 5a).

Each component represented a new and independent source of variability in the data beyond what was already captured in the preceding components (Fig. 5a–j). Building on the previous example, just because the ptosis of eyelids is weakly correlated with PC1 does not mean that this skin ageing phenotype is unimportant. Indeed, we found that ptosis of eyelids is strongly correlated with another PC – PC8 (rotated factor loading score: −0.765) (Fig. 5h). The first ten PCs cumulatively explain 46.88% of the variance in the dataset (Additional File 2).

The first principal component (PC1) explained 17.36% of the variance. Glabellar frown wrinkles, perioral wrinkles, Melomental folds, sagging of the jawline, forehead wrinkles, sagging or wrinkling of the neck skin, an older perceived age, nasolabial folds, Crow’s Feet wrinkles, and the wrinkling aspect of photo-ageing were strongly loaded onto PC1.

The second principal component (PC2) explained a further 7.04% of the variance. The phenotypes strongly loaded onto PC2 were pigment spot, solar comedone, permanent erythema, sebaceous hyperplasia, the dyspigmentation aspect of photo-ageing, cheek skin pores appear larger, seborrheic keratosis, a reduced amount of fat tissue, and a poor cheek laxity.

A further 3.49% of the variance in the dataset was explained by PC3, predominantly by telangiectasia, solar lentigines, and wrinkles under the eyes.

PC4 explains 3.24% of the variance and a poor lip fullness was strongly loaded onto this component.

PC5 explained a further 2.89% of the variance. A lighter skin colour was strongly loaded onto PC5.

The next five principal components each had a single phenotype strongly loaded onto them. Xerosis, ephelides, ptosis of eyelids, eyebags, and a low eyebrow positioning were strongly loaded onto PC6 to PC10 respectively. The percentage of variance explained by PC6 to PC10 were 2.79%, 2.63%, 2.53%, 2.48%, and 2.45% respectively (Additional File 2).

We also questioned how skin ageing phenotypes load beyond PC10. We found that the phenotype ‘venous lakes’ loaded most strongly onto PC11. However, the loading magnitude was not strong (loading factor = 0.52). Similarly, for PC12, the loading factor of ‘guttate hypomelanosis’ on PC12 was 0.53, below the 0.70 threshold earlier defined. We interpreted these results as that the first ten PCs captured the main sources of variability in the data. Although PC11 and beyond reveal additional sources of variability, these were smaller and cannot be clearly attributed to specific groups of phenotypes or individual phenotypes.

### Characterisation of index phenotypes from the first ten PCs

As skin ageing is a complex process, a representative overview of this complex topic requires the discussion of many distinct forms of skin ageing. We define an index phenotype as the phenotype with the strongest loading onto each PC. The index phenotypes for PC1 to PC10 are glabellar frown wrinkles, pigment spots, telangiectasia, poor lip fullness, lighter skin colour, xerosis, ephelides, ptosis of eyelids, eyebags, and low eyebrow positioning respectively.

**Fig. 6:**
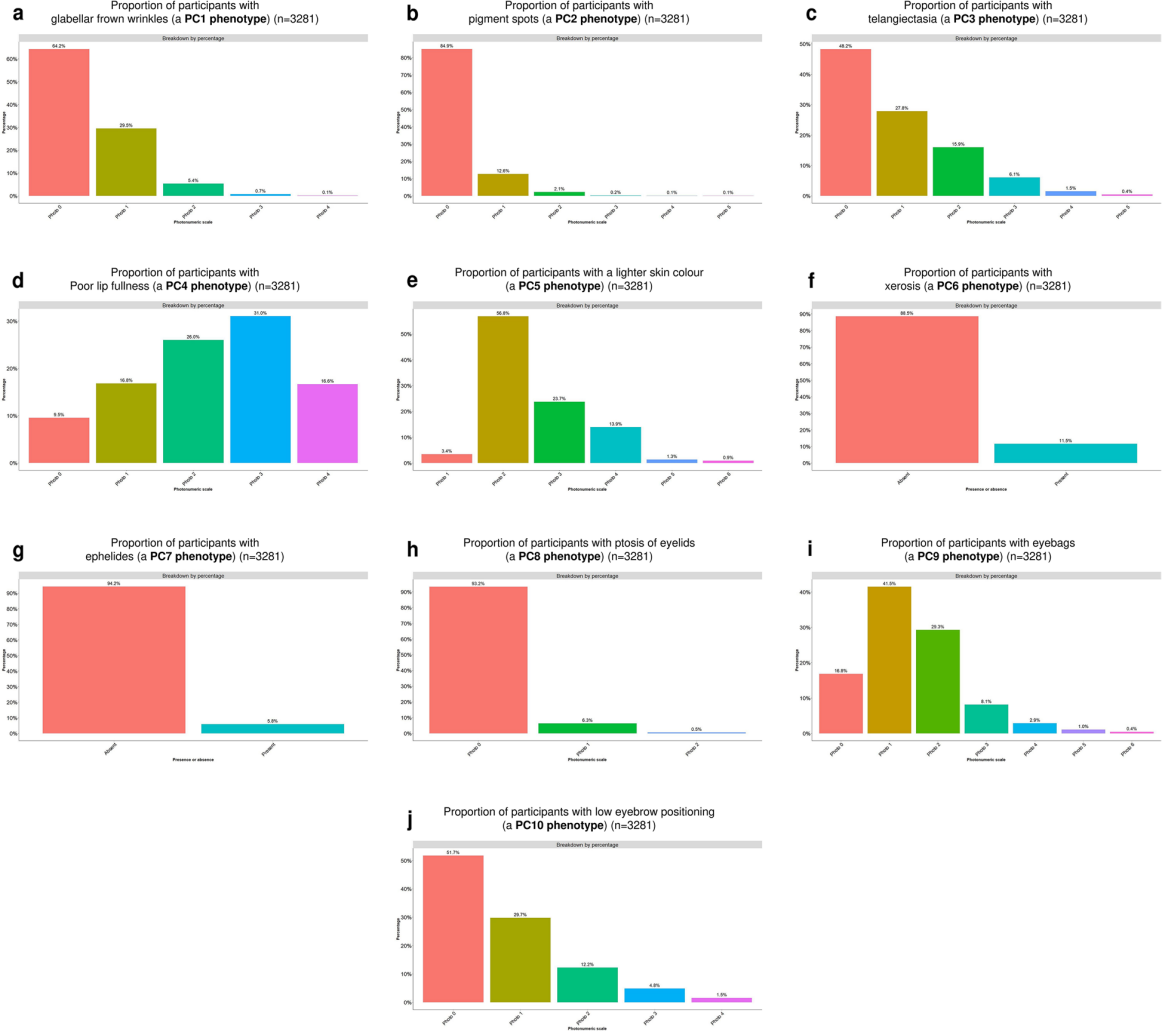
**a–j** A detailed characterisation of the proportion of ethnic Chinese participants from the Singapore/Malaysia Cross-sectional Genetics Epidemiology Study (SMCGES) (*n* = 3,281) with **(a)** glabellar frown wrinkles, **b** pigment spots, **c** telangiectasia, **d** a poor lip fullness, **e** a lighter skin colour, **f** xerosis, **g** ephelides, **h** ptosis of eyelids, **i** eyebags, and **j** low eyebrow positioning. Larger numbers on the photo-numeric scales indicate higher severity. Binary traits are evaluated as either present or absent

We will characterise the ten index phenotypes in greater detail (Fig. 6a–j).

Glabellar frown wrinkles were present in a third (*n* = 1173/3281, 35.8%) of our study population. Glabellar frown wrinkles can be mild (i.e., photo 1), moderate, or severe (i.e., photos 2 and above). Nearly three in ten participants (*n* = 969/3281, 29.5%) had mild glabellar frown lines. We observed moderate and severe glabellar frown wrinkles in 6.2% (*n* = 204/3281) participants.

Pigment spots were seen in 494 participants (*n* = 494/3281, 15.1%). The spots were mostly small and few (i.e., photo 1) (*n* = 413/3281, 12.6%).

Telangiectasia was present in more than half (51.8%) (*n* = 1698/3281) of our study population. Telangiectasia was classified as mild (i.e., photo 1) or severe (i.e., photos 2 and above). A quarter (*n* = 913/3281, 27.8%) of our study population had mild telangiectasia and another quarter (*n* = 785/3281, 23.9%) had severe telangiectasia.

Lip fullness can be evaluated as full (i.e., photo 4) or poor (i.e., photos 0–3). 16.6% (*n* = 546/3281) of our participants had full lip fullness. One in ten (*n* = 313/3281, 9.5%) participants had the poorest lip fullness.

Next, we studied skin colour. 3.4% (*n* = 111/3281) of our participants had the lightest shade of skin colour. Photo 2, a slightly darker shade of the skin, was the skin colour for more than half of our participants (*n* = 1864/3281, 56.8%). Four in ten participants (*n* = 1306/3281, 39.8%) had even darker skin.

It had been previously reported that older people may experience a severe form of skin dryness (i.e., xerosis) as epidermal barrier permeability decreases and trans-epidermal water loss (TEWL) increases with age [28, 29]. Xerosis was reported in one-tenth (*n* = 377/3281, 11.5%) of our participants.

Ephelides (i.e., freckles) is a skin ageing phenotype and the presence of freckles is an indicator of photo-damage from the Sun [30]. 191 participants (*n* = 191/3281, 5.8%) had ephelides.

Ptosis of eyelids (i.e., droopy eyelids) was defined as absent (i.e., photo 0), mild (i.e., photo 1), or severe (i.e., photo 2). 206 participants (*n* = 206/3281, 6.3%) and 17 participants (*n* = 17/3281, 0.5%) had mild and severe ptosis of the eyelids respectively.

Eyebags were quantified as absent (i.e., photo 0), mild (i.e., photos 1–2), moderate (i.e., photos 3–4), or severe (i.e., photos 5–6). Eyebags were reported in 2730 study participants (*n* = 2730/3281, 83%). The majority (*n* = 2321/3281, 70.8%) of participants had mild eyebags. Moderate and severe eyebags were reported in 361 participants (*n* = 361/3281, 11.0%) and 48 participants (*n* = 48/3281, 1.4%) respectively.

Lastly, we quantified sagging of the eyebrows. Eyebrow positioning was quantified as high (i.e., photo 0), or low (i.e., photos 1 and above). One in two participants (*n* = 1585/3281, 48.3%) reported low eyebrow positioning.

## Discussion

### Skin ageing is more than just wrinkles, pigmentation, and photo-ageing

In this broad assessment of skin ageing phenotypes, we found that nearly three-quarters of the evaluated skin ageing phenotypes had a prevalence of ≥ 5% in our study population (Fig. 1). This finding was consistent with our previous discoveries that many skin ageing phenotypes have early onsets and that participants as young as 18 years old already experience multiple forms of skin ageing [17, 18].

Using PCA on a broad spectrum of skin ageing phenotypes, we discovered that the commonly discussed forms of skin ageing (e.g., wrinkles, pigmentation, photo-ageing) only accounted for a small portion of the variance of all skin ageing phenotypes in our study population. We interpreted this result as evidence that skin ageing phenotypes comprise more than just wrinkles, pigmentation and photo-ageing. By focusing on only a small handful of skin ageing phenotypes, the current discourse on skin ageing does not sufficiently recognise the true extent of how complex skin ageing really is.

We sought to understand which phenotypes were more closely interrelated than others, and therefore, may share similar origins. The first ten PCs cumulatively explained nearly half of the variance of skin ageing phenotypes in our study population, and we speculate that each PC has its own distinct underlying mechanism. For instance, a mechanistic explanation for wrinkles describes how two things change with age: first, the facial skin envelope deteriorates, and second, facial mimetic muscles pull on the skin envelope with weaker strengths. Together, they result in hyperdynamic expressions [31]. Our current study examined at least nine types of wrinkles, and we found evidence that six of them (glabellar frown wrinkles, perioral wrinkles, Melomental folds, forehead wrinkles, nasolabial folds, Crow’s Feet wrinkles) were interrelated with sagging skin and photo-ageing (i.e., PC1). Through our findings, we speculate that the earlier described mechanism may influence the phenotypic presentation of sagging skin and photo-ageing as well.

### Negative correlations between ‘poor lip fullness’, ‘a lighter skin colour’, and other skin ageing phenotypes

Earlier in Sect. 11.3 (Concordance among skin ageing phenotypes), we observed that all skin ageing phenotypes were positively correlated with one another except for ‘poor lip fullness’ and ‘a lighter skin colour’. ‘Poor lip fullness’ is the index phenotype of PC4 and ‘a lighter skin colour’ is the index phenotype of PC5.

Positive correlations between a pair of phenotypes indicate that when one phenotype increases in severity, the other phenotype also increases in severity. For example, since glabellar frown wrinkles and forehead wrinkles are positively correlated, more severe glabellar frown wrinkles usually occur alongside more severe forehead wrinkles.

‘Poor lip fullness’ was (i) negatively correlated with most skin ageing phenotypes, though (ii) this correlation is weak. These two points will be discussed separately. (i) Being negatively correlated means that while participants (including younger participants) have wrinkles (i.e., PC1), these participants tend not to have a poor lip fullness (i.e., PC4). This is consistent with our hypothesis that each PC has its own distinct underlying mechanism, with one mechanism affecting wrinkles and another mechanism bringing about a poor lip fullness. (ii) It is known that lips become thinner with age due to a gradual loss of collagen. Collagen depletion occurs as early as 20 years old [32]. About 1%−2% of collagen is lost from the lips yearly. This depletion of collagen causes lips to change from their natural full shape to a thinner appearance as a result [32]. The thinning of the lips is an inevitable part of ageing just like wrinkles are. Therefore, we hypothesise that even if negative correlations exist between ‘poor lip fullness’ and other skin ageing phenotypes, these correlations were unlikely to be strong ones. Our findings that the negative correlation was weak, not strong, support our hypothesis.

‘A lighter skin colour’ was (i) negatively correlated with most skin ageing phenotypes, though (ii) this correlation is weak. These two points will be discussed separately. (i) It is well-known that an increased exposure to UV light both darkens and ages the skin [33] through the production of melanin. Melanin is a well-known photoprotective factor as it is a broadband UV absorbent, is an antioxidant, and can scavenge free radicals [34]. Seen from this perspective, a darker skin colour is protective against skin ageing and the reverse promotes skin ageing. Many ethnic Chinese participants, however, do not have dark skin. The presence of many participants with skin ageing phenotypes (e.g., wrinkles) and light skin colour in the dataset could contribute to the negative correlation between ‘a lighter skin colour’ and most skin ageing phenotypes. (ii) As mentioned above, cumulative lifetime UV exposure darkens the skin. Likewise, it is intuitive to understand that the skin ages with the passage of time. Therefore, we hypothesise that even if any negative correlations exist between ‘a lighter skin colour’ and other skin ageing phenotypes, they were unlikely to be strong ones. Our findings that the negative correlation was weak, not strong, support our hypothesis.

In summary, ‘poor lip fullness’ and ‘a lighter skin colour’ were negatively correlated with all other skin ageing phenotypes for different reasons. ‘Poor lip fullness’ and ‘a lighter skin colour’ were the only two phenotypes showing negative correlations with all other skin ageing phenotypes. As expected, the correlation between ‘poor lip fullness’ and ‘a lighter skin colour’ was a positive one.

### Comparing younger participants with older participants

One study limitation was that older participants aged 40 and above were not sufficiently represented in the current assessment, making up about 3% of our total sample size. We analysed the skin ageing phenotypic profile of 101 participants aged 40 and above and contrasted it against younger participants (Additional Files 3–4). This revealed three key observations.

Firstly, the prevalence rates of various skin ageing phenotypes in the older participants can be broadly divided into two starkly different groups – (i) phenotypes from ‘low eyebrow positioning’ to ‘eyebags’ have prevalence rates between 76% and 95%) while (ii) phenotypes from ‘pseudoscar’ to ‘ptosis of eyelids’ were only seen in 1% to 34% of older participants (Additional File 3). In contrast, the phenotypes exhibited by younger participants (i.e., participants aged below 40) can also be divided into two groups but the difference between the two groups was more gradual – (iii) phenotypes from ‘photo-ageing (wrinkling)’ to ‘poor lip fullness’ have prevalence rates between 26% and 83% while (iv) phenotypes from ‘pseudoscar’ to ‘solar comedone’ were seen in 0.2% to 16% of younger participants (Additional File 4).

Next, we observed that the index phenotypes of PC1, PC3, PC4, PC9, and PC10 were highly prevalent in young people. The same five index phenotypes were also highly prevalent in older people. The reverse was also true; the index phenotypes of PC2, and PC5 through PC8 were less prevalent in our participants regardless of age.

The final observation that we found was that while the prevalence rate of many skin ageing phenotypes ranges between 46% and 56% in the younger participants, the prevalence rate increased sharply to between 76% and 95% in older participants. This finding corroborated with our earlier works which reported trends showing that skin ageing phenotypes persistently deteriorated with age from 18 to 40 [17, 18]. We predict that our identified trends between younger and older participants will persist. With progressive annual recruitment drives to expand the SMCGES, we plan to explore whether the trends for older participants identified in this study will stabilise and to identify additional trends that may emerge.

### Correlation between PCs and demographic factors (age, sex)

We first studied the correlation between non-modifiable risk factors (age and sex) and the PCs.

An older chronological age had the strongest positive correlation with PC1 (Pearson’s correlation coefficient = 0.430, *p*-value = 5.17 × 10^−148^). Wrinkles are the key characteristic of PC1 and the index phenotype of PC1 is ‘glabellar frown wrinkles’. It is also intuitive to understand that wrinkles become more severe with increasing chronological age. Aside from PC1, chronological age had fairly strong correlations with PC2, PC3, PC6, PC7, and PC9 (Table 2 and Additional Files 5–14).

**Table 2:** Correlation between the PCs extracted from skin ageing traits and age and sex

Principal Component	Age		Sex	
	**Pearson’s Correlation Coefficient**	***p*-value**	**Pearson’s Correlation Coefficient**	***p*-value**
PC1	0.430	5.17 × 10^−148^	− 0.133	1.73 × 10^−14^
PC2	− 0.384	6.39 × 10^−116^	0.071	4.48 × 10^−5^
PC3	− 0.336	1.39 × 10^−87^	− 0.025	1.55 × 10^−1^
PC4	− 0.053	2.53 × 10^−3^	− 0.104	2.70 × 10^−9^
PC5	− 0.174	1.24 × 10^−23^	0.075	1.75 × 10^−5^
PC6	0.310	5.33 × 10^−74^	0.180	2.07 × 10^−25^
PC7	− 0.229	3.63 × 10^−40^	− 0.014	4.33 × 10^−1^
PC8	0.061	5.07 × 10^−4^	− 0.048	5.95 × 10^−3^
PC9	0.244	7.50 × 10^−46^	− 0.034	5.01 × 10^−2^
PC10	0.014	4.15 × 10^−1^	− 0.066	1.58 × 10^−4^

Sex was only weakly correlated with PC6 (*r*_*pb*_ = 0.180, *p*-value = 2.07 × 10^−25^) and no other PCs (Table 2). However, two-tailed t-tests show that males and females have significantly different PC values except for ephelides (PC7), ptosis of eyelids (PC8), and eyebags (PC9) (Additional Files 15–24). While the relationship between sex and the pathophysiology of many skin ageing phenotypes is not clear, the role of sex hormones has been implicated and reviewed previously [35]. We identified two possible reasons related to the female sex: menopausal status and hormone replacement therapy (HRT) use. Both factors have been found and reported in our earlier meta-analysis and systematic review to interact with increased chronological age, influencing the manifestation of skin ageing phenotypes [1].

### Correlation between PCs and income and stress

To study the effects of income, we stratified the PCA values by the self-reported total monthly family income per capita of the participants. Income levels are significantly associated with differences in PCA values for PC2 (index phenotype: pigment spots), PC6 (index phenotype: xerosis), PC7 (index phenotype: ephelides), and PC9 (index phenotype: eyebags) (Additional Files 25–34).

Stress can take several forms including financial stress, psychological stress, or stress in general. Studies by Agrigoroaei et al*.* (2017) [36] and Lee et al*.* (2020) [37] respectively found that financial stress and psychological stress were significantly associated with older perceived ages. A different study on African Americans also found an association between high lifetime stress exposure and premature ageing [38]. We therefore wondered whether the PCs reflect general ageing conditions or the amount of stress an individual has.

To study the effects of stress, we stratified the PCA values by perceived stress levels measured by the PSS. Perceived stress was significantly associated with differences in PCA values for PC2 (index phenotype: pigment spots), PC5 (index phenotype: a lighter skin colour), and PC10 (index phenotype: low eyebrow positioning) (Additional Files 35–44).

Importantly, PC2 values were significantly different across income levels and stress levels. The second PC mainly consists of age-related pigmentary changes (e.g., pigment spots, solar comedone, permanent erythema, sebaceous hyperplasia, and the dyspigmentation aspect of photo-ageing). A more detailed analysis needs to be done to explore whether PC2 is related to financial stress. This could involve examining additional aspects of financial stress, such as participants’ average sleep duration and quality, as well as their occupations.

## Conclusion

While researchers agree that skin ageing is complex, no study quantified this complexity in detail. We explored the interrelationship among skin ageing phenotypes and characterised them. Oversimplifying skin ageing to wrinkles, pigmentation, and photo-ageing neglects other phenotypes which are just as important in addressing the gaps in our knowledge to create a comprehensive understanding of skin ageing. The addition of these other phenotypes adds more dimensions to skin ageing phenotype presentations.

Key questions remain. Could highly correlated phenotypes share similar underlying genetics? Future genetic studies may shed light on other mechanisms underlying skin ageing phenotypes from different PCs.

## Supplementary Information

Additional file 1. Identification of skin ageing phenotypes and how they were evaluated.

Additional file 2. Total variance explained by each of the forty-one principal components (PCs).

Additional file 3. Prevalence of forty-one skin ageing phenotypes from ethnic Chinese participants aged 40 years and above from the Singapore/Malaysia Cross-sectional Genetics Epidemiology Study (SMCGES) (*n*=101).

Additional file 4. Prevalence of forty-one skin ageing phenotypes from ethnic Chinese participants aged below 40 from the Singapore/Malaysia Cross-sectional Genetics Epidemiology Study (SMCGES) (*n*=3180).

Additional file 5. Correlation between chronological age and PC1 values. Pearson’s correlation coefficients are computed for each plot. *p*-values reported are two-tailed Pearson’s correlation *p*-values. A line of goodness of fit is included, based on a linear regression model, with the R² coefficient of determination displayed for each plot.

Additional file 6. Correlation between chronological age and PC2 values. Pearson’s correlation coefficients are computed for each plot. *p*-values reported are two-tailed Pearson’s correlation *p*-values. A line of goodness of fit is included, based on a linear regression model, with the R² coefficient of determination displayed for each plot.

Additional file 7. Correlation between chronological age and PC3 values. Pearson’s correlation coefficients are computed for each plot. *p*-values reported are two-tailed Pearson’s correlation *p*-values. A line of goodness of fit is included, based on a linear regression model, with the R² coefficient of determination displayed for each plot.

Additional file 8. Correlation between chronological age and PC4 values. Pearson’s correlation coefficients are computed for each plot. *p*-values reported are two-tailed Pearson’s correlation *p*-values. A line of goodness of fit is included, based on a linear regression model, with the R² coefficient of determination displayed for each plot.

Additional file 9. Correlation between chronological age and PC5 values. Pearson’s correlation coefficients are computed for each plot. *p*-values reported are two-tailed Pearson’s correlation *p*-values. A line of goodness of fit is included, based on a linear regression model, with the R² coefficient of determination displayed for each plot.

Additional file 10. Correlation between chronological age and PC6 values. Pearson’s correlation coefficients are computed for each plot. *p*-values reported are two-tailed Pearson’s correlation *p*-values. A line of goodness of fit is included, based on a linear regression model, with the R² coefficient of determination displayed for each plot.

Additional file 11. Correlation between chronological age and PC7 values. Pearson’s correlation coefficients are computed for each plot. *p*-values reported are two-tailed Pearson’s correlation *p*-values. A line of goodness of fit is included, based on a linear regression model, with the R² coefficient of determination displayed for each plot.

Additional file 12. Correlation between chronological age and PC8 values. Pearson’s correlation coefficients are computed for each plot. *p*-values reported are two-tailed Pearson’s correlation *p*-values. A line of goodness of fit is included, based on a linear regression model, with the R² coefficient of determination displayed for each plot.

Additional file 13. Correlation between chronological age and PC9 values. Pearson’s correlation coefficients are computed for each plot. *p*-values reported are two-tailed Pearson’s correlation *p*-values. A line of goodness of fit is included, based on a linear regression model, with the R² coefficient of determination displayed for each plot.

Additional file 14. Correlation between chronological age and PC10 values. Pearson’s correlation coefficients are computed for each plot. *p*-values reported are two-tailed Pearson’s correlation *p*-values. A line of goodness of fit is included, based on a linear regression model, with the R² coefficient of determination displayed for each plot.

Additional file 15. PC1 values stratified by sex. Two-tailed t-test p-values are computed for each plot. The median PC values for both sexes are displayed in each plot. *p*-values reported are two-tailed t-test *p*-values, with * indicating *p* < 0.05, ** *p* < 0.01, and *** *p* < 0.001. *p* > was considered statistically non-significant (ns).

Additional file 16. PC2 values stratified by sex. Two-tailed t-test *p*-values are computed for each plot. The median PC values for both sexes are displayed in each plot. *p*-values reported are two-tailed t-test *p*-values, with * indicating *p* < 0.05, ** *p* < 0.01, and *** *p* < 0.001. *p* > was considered statistically non-significant (ns).

Additional file 17. PC3 values stratified by sex. Two-tailed t-test *p*-values are computed for each plot. The median PC values for both sexes are displayed in each plot. *p*-values reported are two-tailed t-test *p*-values, with * indicating *p*  < 0.05, ** *p* < 0.01, and *** *p* < 0.001. *p* > was considered statistically non-significant (ns).

Additional file 18. PC4 values stratified by sex. Two-tailed t-test *p*-values are computed for each plot. The median PC values for both sexes are displayed in each plot. *p* -values reported are two-tailed t-test *p* -values, with * indicating *p*  < 0.05, ** *p* < 0.01, and *** *p* < 0.001. *p* > was considered statistically non-significant (ns).

Additional file 19. PC5 values stratified by sex. Two-tailed t-test *p*-values are computed for each plot. The median PC values for both sexes are displayed in each plot. *p*-values reported are two-tailed t-test *p*-values, with * indicating *p* < 0.05, ** *p* < 0.01, and *** *p* < 0.001. *p* > was considered statistically non-significant (ns).

Additional file 20. PC6 values stratified by sex. Two-tailed t-test *p*-values are computed for each plot. The median PC values for both sexes are displayed in each plot. *p*-values reported are two-tailed t-test *p*-values, with * indicating *p* < 0.05, ** *p* < 0.01, and *** *p* < 0.001. *p* > was considered statistically non-significant (ns).

Additional file 21. PC7 values stratified by sex. Two-tailed t-test *p*-values are computed for each plot. The median PC values for both sexes are displayed in each plot. *p*-values reported are two-tailed t-test *p*-values, with * indicating *p* < 0.05, ** *p* < 0.01, and *** *p* < 0.001. *p* > was considered statistically non-significant (ns).

Additional file 22.  PC8 values stratified by sex. Two-tailed t-test *p*-values are computed for each plot. The median PC values for both sexes are displayed in each plot. *p*-values reported are two-tailed t-test *p*-values, with * indicating *p* < 0.05, ** *p* < 0.01, and *** *p* < 0.001. *p* > was considered statistically non-significant (ns).

Additional file 23. PC9 values stratified by sex. Two-tailed t-test *p*-values are computed for each plot. The median PC values for both sexes are displayed in each plot. *p*-values reported are two-tailed t-test *p*-values, with * indicating *p* < 0.05, ** *p* < 0.01, and *** *p* < 0.001. *p* > was considered statistically non-significant (ns).

Additional file 24. PC10 values stratified by sex. Two-tailed t-test *p*-values are computed for each plot. The median PC values for both sexes are displayed in each plot. *p*-values reported are two-tailed t-test *p*-values, with * indicating *p* < 0.05, ** *p* < 0.01, and *** *p* < 0.001. *p* > was considered statistically non-significant (ns).

Additional file 25. PC1 values grouped by income level. Two-tailed t-test *p*-values are provided for each plot. Income levels are based on self-reported total monthly family income per capita. In Singapore, income levels are classified as follows: low (< SGD 2000), moderate (SGD 2000–3999), high (SGD 4000–5999), and very high (> SGD 6000). In Malaysia, the classifications are low (< RM 3000), middle (RM 3000–5999), high (RM 6000–12,999), and very high (> RM 13,000). Each plot displays the median PC values for low, middle, high, and very high income groups.*p*-values reported are two-tailed t-test *p*-values, with * indicating *p* < 0.05, ** *p* < 0.01, and *** *p* < 0.001. *p* > 0.05 was considered statistically non-significant (ns).

Additional file 26. PC2 values grouped by income level. Two-tailed t-test *p*-values are provided for each plot. Income levels are based on self-reported total monthly family income per capita. In Singapore, income levels are classified as follows: low (< SGD 2000), moderate (SGD 2000–3999), high (SGD 4000–5999), and very high (> SGD 6000). In Malaysia, the classifications are low (< RM 3000), middle (RM 3000–5999), high (RM 6000–12,999), and very high (> RM 13,000). Each plot displays the median PC values for low, middle, high, and very high income groups.*p*-values reported are two-tailed t-test *p*-values, with * indicating *p* < 0.05, ** *p* < 0.01, and *** *p* < 0.001. *p* > 0.05 was considered statistically non-significant (ns).

Additional file 27. PC3 values grouped by income level. Two-tailed t-test *p*-values are provided for each plot. Income levels are based on self-reported total monthly family income per capita. In Singapore, income levels are classified as follows: low (< SGD 2000), moderate (SGD 2000–3999), high (SGD 4000–5999), and very high (> SGD 6000). In Malaysia, the classifications are low (< RM 3000), middle (RM 3000–5999), high (RM 6000–12,999), and very high (> RM 13,000). Each plot displays the median PC values for low, middle, high, and very high income groups.*p*-values reported are two-tailed t-test *p*-values, with * indicating *p* < 0.05, ** *p* < 0.01, and *** *p* < 0.001. *p* > 0.05 was considered statistically non-significant (ns).

Additional file 28. PC4 values grouped by income level. Two-tailed t-test *p*-values are provided for each plot. Income levels are based on self-reported total monthly family income per capita. In Singapore, income levels are classified as follows: low (< SGD 2000), moderate (SGD 2000–3999), high (SGD 4000–5999), and very high (> SGD 6000). In Malaysia, the classifications are low (< RM 3000), middle (RM 3000–5999), high (RM 6000–12,999), and very high (> RM 13,000). Each plot displays the median PC values for low, middle, high, and very high income groups.*p*-values reported are two-tailed t-test *p*-values, with * indicating *p* < 0.05, ** *p* < 0.01, and *** *p* < 0.001. *p* > 0.05 was considered statistically non-significant (ns).

Additional file 29. PC5 values grouped by income level. Two-tailed t-test *p*-values are provided for each plot. Income levels are based on self-reported total monthly family income per capita. In Singapore, income levels are classified as follows: low (< SGD 2000), moderate (SGD 2000–3999), high (SGD 4000–5999), and very high (> SGD 6000). In Malaysia, the classifications are low (< RM 3000), middle (RM 3000–5999), high (RM 6000–12,999), and very high (> RM 13,000). Each plot displays the median PC values for low, middle, high, and very high income groups.*p*-values reported are two-tailed t-test *p*-values, with * indicating *p* < 0.05, ** *p* < 0.01, and *** *p* < 0.001. *p* > 0.05 was considered statistically non-significant (ns).

Additional file 30. PC6 values grouped by income level. Two-tailed t-test *p*-values are provided for each plot. Income levels are based on self-reported total monthly family income per capita. In Singapore, income levels are classified as follows: low (< SGD 2000), moderate (SGD 2000–3999), high (SGD 4000–5999), and very high (> SGD 6000). In Malaysia, the classifications are low (< RM 3000), middle (RM 3000–5999), high (RM 6000–12,999), and very high (> RM 13,000). Each plot displays the median PC values for low, middle, high, and very high income groups.*p*-values reported are two-tailed t-test *p*-values, with * indicating *p* < 0.05, ** *p* < 0.01, and *** *p* < 0.001. *p* > 0.05 was considered statistically non-significant (ns).

Additional file 31. PC7 values grouped by income level. Two-tailed t-test *p*-values are provided for each plot. Income levels are based on self-reported total monthly family income per capita. In Singapore, income levels are classified as follows: low (< SGD 2000), moderate (SGD 2000–3999), high (SGD 4000–5999), and very high (> SGD 6000). In Malaysia, the classifications are low (< RM 3000), middle (RM 3000–5999), high (RM 6000–12,999), and very high (> RM 13,000). Each plot displays the median PC values for low, middle, high, and very high income groups.*p*-values reported are two-tailed t-test p-values, with * indicating *p* < 0.05, ** *p* < 0.01, and *** *p* < 0.001. *p* > 0.05 was considered statistically non-significant (ns).

Additional file 32. PC8 values grouped by income level. Two-tailed t-test *p*-values are provided for each plot. Income levels are based on self-reported total monthly family income per capita. In Singapore, income levels are classified as follows: low (< SGD 2000), moderate (SGD 2000–3999), high (SGD 4000–5999), and very high (> SGD 6000). In Malaysia, the classifications are low (< RM 3000), middle (RM 3000–5999), high (RM 6000–12,999), and very high (> RM 13,000). Each plot displays the median PC values for low, middle, high, and very high income groups.*p*-values reported are two-tailed t-test *p*-values, with * indicating *p* < 0.05, ** *p* < 0.01, and *** *p* < 0.001. *p* > 0.05 was considered statistically non-significant (ns).

Additional file 33. PC9 values grouped by income level. Two-tailed t-test *p*-values are provided for each plot. Income levels are based on self-reported total monthly family income per capita. In Singapore, income levels are classified as follows: low (< SGD 2000), moderate (SGD 2000–3999), high (SGD 4000–5999), and very high (> SGD 6000). In Malaysia, the classifications are low (< RM 3000), middle (RM 3000–5999), high (RM 6000–12,999), and very high (> RM 13,000). Each plot displays the median PC values for low, middle, high, and very high income groups.*p*-values reported are two-tailed t-test *p*-values, with * indicating *p* < 0.05, ** *p* < 0.01, and *** *p* < 0.001. *p* > 0.05 was considered statistically non-significant (ns).

Additional file 34. PC10 values grouped by income level. Two-tailed t-test *p*-values are provided for each plot. Income levels are based on self-reported total monthly family income per capita. In Singapore, income levels are classified as follows: low (< SGD 2000), moderate (SGD 2000–3999), high (SGD 4000–5999), and very high (> SGD 6000). In Malaysia, the classifications are low (< RM 3000), middle (RM 3000–5999), high (RM 6000–12,999), and very high (> RM 13,000). Each plot displays the median PC values for low, middle, high, and very high income groups.*p*-values reported are two-tailed t-test *p*-values, with * indicating *p* < 0.05, ** *p* < 0.01, and *** *p* < 0.001. *p* > 0.05 was considered statistically non-significant (ns).

Additional file 35. PC1 values stratified by perceived stress level. Two-tailed t-test *p*-values are computed for each plot. Perceived stress levels are based on self-reported scores from the Perceived Stress Scale (PSS): low stress (0–13), moderate stress (14–26), and high stress (27–40). The median PC values for low stress, moderate stress, and high stress levels are displayed in each plot. *p*-values reported are two-tailed t-test *p*-values, with * indicating *p*< 0.05, ** *p* < 0.01, and *** *p* < 0.001. *p* > 0.05 was considered statistically non-significant (ns).

Additional file 36. PC2 values stratified by perceived stress level. Two-tailed t-test *p*-values are computed for each plot. Perceived stress levels are based on self-reported scores from the Perceived Stress Scale (PSS): low stress (0–13), moderate stress (14–26), and high stress (27–40). The median PC values for low stress, moderate stress, and high stress levels are displayed in each plot. *p*-values reported are two-tailed t-test *p*-values, with * indicating *p*< 0.05, ** *p* < 0.01, and *** *p* < 0.001. *p* > 0.05 was considered statistically non-significant (ns).

Additional file 37. PC3 values stratified by perceived stress level. Two-tailed t-test *p*-values are computed for each plot. Perceived stress levels are based on self-reported scores from the Perceived Stress Scale (PSS): low stress (0–13), moderate stress (14–26), and high stress (27–40). The median PC values for low stress, moderate stress, and high stress levels are displayed in each plot. *p*-values reported are two-tailed t-test *p*-values, with * indicating *p*< 0.05, ** *p* < 0.01, and *** *p* < 0.001. *p* > 0.05 was considered statistically non-significant (ns).

Additional file 38. PC4 values stratified by perceived stress level. Two-tailed t-test *p*-values are computed for each plot. Perceived stress levels are based on self-reported scores from the Perceived Stress Scale (PSS): low stress (0–13), moderate stress (14–26), and high stress (27–40). The median PC values for low stress, moderate stress, and high stress levels are displayed in each plot. *p*-values reported are two-tailed t-test *p*-values, with * indicating *p*< 0.05, ** *p* < 0.01, and *** *p* < 0.001. *p* > 0.05 was considered statistically non-significant (ns).

Additional file 39. PC5 values stratified by perceived stress level. Two-tailed t-test *p*-values are computed for each plot. Perceived stress levels are based on self-reported scores from the Perceived Stress Scale (PSS): low stress (0–13), moderate stress (14–26), and high stress (27–40). The median PC values for low stress, moderate stress, and high stress levels are displayed in each plot. *p*-values reported are two-tailed t-test *p*-values, with * indicating *p*< 0.05, ** *p* < 0.01, and *** *p* < 0.001. *p* > 0.05 was considered statistically non-significant (ns).

Additional file 40. PC6 values stratified by perceived stress level. Two-tailed t-test *p*-values are computed for each plot. Perceived stress levels are based on self-reported scores from the Perceived Stress Scale (PSS): low stress (0–13), moderate stress (14–26), and high stress (27–40). The median PC values for low stress, moderate stress, and high stress levels are displayed in each plot. *p*-values reported are two-tailed t-test *p*-values, with * indicating *p*< 0.05, ** *p* < 0.01, and *** *p* < 0.001. *p* > 0.05 was considered statistically non-significant (ns).

Additional file 41. PC7 values stratified by perceived stress level. Two-tailed t-test *p*-values are computed for each plot. Perceived stress levels are based on self-reported scores from the Perceived Stress Scale (PSS): low stress (0–13), moderate stress (14–26), and high stress (27–40). The median PC values for low stress, moderate stress, and high stress levels are displayed in each plot. *p*-values reported are two-tailed t-test *p*-values, with * indicating *p*< 0.05, ** *p* < 0.01, and *** *p* < 0.001. *p* > 0.05 was considered statistically non-significant (ns).

Additional file 42. PC8 values stratified by perceived stress level. Two-tailed t-test *p*-values are computed for each plot. Perceived stress levels are based on self-reported scores from the Perceived Stress Scale (PSS): low stress (0–13), moderate stress (14–26), and high stress (27–40). The median PC values for low stress, moderate stress, and high stress levels are displayed in each plot. *p*-values reported are two-tailed t-test *p*-values, with * indicating *p*< 0.05, ** *p* < 0.01, and *** *p* < 0.001. *p* > 0.05 was considered statistically non-significant (ns).

Additional file 43. PC9 values stratified by perceived stress level. Two-tailed t-test *p*-values are computed for each plot. Perceived stress levels are based on self-reported scores from the Perceived Stress Scale (PSS): low stress (0–13), moderate stress (14–26), and high stress (27–40). The median PC values for low stress, moderate stress, and high stress levels are displayed in each plot. *p*-values reported are two-tailed t-test *p*-values, with * indicating *p*< 0.05, ** *p* < 0.01, and *** *p* < 0.001. *p* > 0.05 was considered statistically non-significant (ns).

Additional file 44. PC10 values stratified by perceived stress level. Two-tailed t-test *p*-values are computed for each plot. Perceived stress levels are based on self-reported scores from the Perceived Stress Scale (PSS): low stress (0–13), moderate stress (14–26), and high stress (27–40). The median PC values for low stress, moderate stress, and high stress levels are displayed in each plot. *p*-values reported are two-tailed t-test *p*-values, with * indicating *p*< 0.05, ** *p* < 0.01, and *** *p* < 0.001. *p* > 0.05 was considered statistically non-significant (ns).

## Data Availability

All data generated or analysed during this study are included in this published article and its supplementary information files. Further inquiries about the datasets used and/or analysed during the current study can be directed to the corresponding author (F.T.C.) on reasonable request.

## Abbreviations

BMI: Body mass index
cm: Centimetres
IBM SPSS/PC: International Business Machines Corporation Statistical Package for Social Scientists Personal Computer (SPSS/PC)
kg: Kilograms
kg/m^2^: Kilograms per square metre
PC: Principal Component
PCA: Principal Component Analysis
SD: Standard deviation
SMCGES: Singapore/Malaysia Cross-sectional Genetics Epidemiology Study
y: Years
PSS: Perceived Stress Scale
UV light: Ultraviolet light
SGD: Singapore dollars
RM: Malaysian Ringgit
