# Supplementary material for: A broad assessment of forty-one skin phenotypes reveals complex dimensions of skin ageing
Source: J Physiol Anthropol. 2025 Feb 8;44:3. doi: 10.1186/s40101-024-00383-2 (PMC11806859; doi:10.1186/s40101-024-00383-2)
Supplement: Supplementary file 1 — Additional file 1. Identification of skin ageing phenotypes and how they were evaluated. [file 40101_2024_383_MOESM1_ESM.docx]

**Additional file 1:** Identification of skin ageing phenotypes and how they were evaluated.

| **Phenotype** | **Our current assessment used the phenotype description(s) from:** |
| --- | --- |
| Actinic keratosis | Goldsmith *et al.* (2011) ^28^, Habif *et al.* (2018) ^29^, and Rattner (1962) ^30^ |
| Basal cell carcinoma | Norman (2008) ^31^, Goldsmith *et al.* (2011) ^28^, and Habif *et al.* (2018) ^29^ |
| Cheek skin pores appear larger | Flament, Frederic & Bazin (2010) ^32^ |
| Cheek laxity | Chen *et al.* (2021) ^33^ |
| Cheek folds | Flament, Frederic & Bazin (2010) ^32^ |
| Cutis rhomboidalis nuchae | Bolognia *et al.* (2012) ^34^ |
| Crow’s Feet wrinkles | Tsukahara *et al.* (2000) ^35^ |
| Ephelides | Ferri *et al.* (2011) ^36^ |
| Eyebags | Ezure *et al.* (2011) ^37^ |
| Favre-Racouchot syndrome | Bolognia *et al.* (2012) ^34^ and Goldsmith *et al.* (2011) ^28^ |
| Forehead wrinkles | Flynn *et al.* (2012) ^38^ |
| Glabellar frown wrinkles | Honeck *et al.* (2003) ^39^ |
| Guttate hypomelanosis | Habif *et al.* (2018) ^29^ |
| Horizontal interocular wrinkles | Flament, Frederic & Bazin (2010) ^32^ |
| Lighter skin colour | Nakashima *et al.* (2022) ^40^ |
| Low eyebrow positioning | Carruthers *et al.* (2008) ^41^ |
| Melanoma | Goldsmith *et al.* (2011) ^28^ and Wolff *et al.* (2017) ^42^ |
| Melasma | Norman (2008) ^31^ and Wolff *et al.* (2017) ^42^ |
| Melomental folds | Narins *et al.* (2012) ^43^ |
| Milia | Habif *et al.* (2018) ^29^ |
| Nasolabial folds | Narins *et al.* (2012) ^43^ |
| Perioral wrinkles | Narins *et al.* (2012) ^43^ |
| Permanent erythema | Bolognia *et al.* (2012) ^34^ |
| Photo-ageing | Larnier *et al.* (1994) ^44^ and Chung *et al.* (2001) ^45^ |
| Pigment spots | Liu *et al.* (2019) ^46^ |
| Poor cheek laxity | Goldsmith *et al.* (2011) ^28^ |
| Poor lip fullness | Narins *et al.* (2012) ^43^ |
| Pseudoscar | Goldsmith *et al.* (2011) ^28^ |
| Ptosis of eyelids | Goldberg & Lew (2011) ^47^ |
| Reduced amount of fat tissue | Lorenc *et al.* (2012) ^48^ |
| Sagging of the jawline | Narins *et al.* (2012) ^43^ |
| Sagging or wrinkling of the neck skin | Sattler *et al.* (2012) ^49^ |
| Sebaceous hyperplasia | Norman (2008) ^31^, Marks (2003) ^50^, and Habif *et al.* (2018) ^29^ |
| Seborrheic keratosis | Bolognia *et al.* (2012) ^34^, Rattner (1962) ^30^, and Habif *et al.* (2018) ^29^ |
| Senile comedone | Marks (2003) ^50^ and Habif *et al.* (2018) ^29^ |
| Senile purpura | Norman (2008) ^31^ and Habif *et al.* (2018) ^29^ |
| Solar elastosis | Bolognia *et al.* (2012) ^34^ and Habif *et al.* (2018) ^29^ |
| Solar lentigines | Chen *et al.* (2021) ^33^ |
| Squamous cell carcinoma | Norman (2008) ^31^, Goldsmith *et al.* (2011) ^28^, and Habif *et al.* (2018) ^29^ |
| Telangiectasia | Chen *et al.* (2021) ^33^ |
| Venous lakes | Norman (2008) ^31^ and Wolff *et al.* (2017) ^42^ |
| Xerosis | Norman (2008) ^31^ and Habif *et al.* (2018) ^29^ |
| Yellowish decolouration of the skin | Habif *et al.* (2018) ^29^ |
| Wrinkles under the eyes | Chen *et al.* (2021) ^33^ |
| Wrinkles | Facial wrinkles were distinguished based on the anatomical site of the face in which they manifested:   - Forehead wrinkles (on the forehead) ^38^, - Glabellar frown wrinkles (vertical wrinkles between the eyebrows) ^38^, - Horizontal interocular wrinkles (horizontal wrinkles between the eyebrows) ^32^, - Crow’s Feet wrinkles (lateral canthal lines spreading out from the corner of the eyes) ^38^, - Wrinkles under the eyes ^23^, - Cheek folds (lines on the cheek) ^32^, - Nasolabial folds (lines running down the ala of the nose to the corner of the mouth) ^43^, - Perioral wrinkles (around the mouth) ^43^, - Melomental folds (drooping of labial commissures in which the corner of the mouth is turned down and creases run down the cheek towards the chin) ^43^. |
| Sagging skin | Sagging phenotypes were characterised by:   - Skin that was no longer firm or tight (e.g., poor cheek laxity) ^28^, - Skin lacking in volume (e.g., sagging of the neck skin) ^49^, - Skin lacking plumpness and elasticity (e.g., reduced amount of fat tissue) ^48^, - Sagging of upper eyelid skin such that it touched the eyelashes, hung over the eyelashes, or hung over the eye (e.g., ptosis of eyelids) ^51^. |
| Pigmentary changes | - Solar lentigines are a notable outward manifestation of skin ageing ^52^. They are defined as benign ^53,54^, single or multiple, discrete, regular or irregular ^55^, round to polycyclic ^56^, brown to black–brown, macular or slightly raised lesions up to 1 cm in diameter. Solar lentigines can be sparse and scattered ^56^, or manifest as a diffused distribution of pigmentation on the skin ^55^. This skin ageing phenotype appears on the face or the back of the hands ^57^. - On the other hand, pigment spots are hyperpigmented areas relative to the surrounding skin ^58^. - Ephelides are a skin ageing phenotype characterised by multiple, small (1–3 mm) ^53,56^ light brown macular lesions which are irregular in shape yet retain distinct borders ^59^. These spots are distinctly pigmented all year round ^59^ and manifests on the neck, shoulder, and the back of the hands ^52^. - Melasma, unlike the others above, is a distinctly brown or dark-coloured patch on the cheeks or the forehead ^31^. Age-related pigmentary changes are not necessarily brown or dark-coloured. - Sebaceous hyperplasia manifests itself as multiple red or flesh-coloured bumps on the forehead which dent inwards ^28,31,50^. - Guttate hypomelanosis appears as single or multiple flat white spots on the skin that resemble teardrops ^28,29^. - Milia are multiple, small (2–3 mm) ^29^, round little flesh-coloured bumps or papules composed of laminated, keratinous material, situated as a solid cyst in a pilosebaceous follicle ^60^ on the cheeks or below the eyes ^29^. |
| Other phenotypes | Several phenotypes, including photo-ageing, solar elastosis, Favre-Racouchot syndrome, and telangiectasia, cannot be neatly categorised as they are more than just wrinkly skin, saggy skin, or skin with age-related pigmentary changes.   - Photo-ageing is cutaneous photodamage to the skin arising from a combination of wrinkling, pigmentary lesions, and coarseness ^44,61,62^. To better capture different facets of photo-ageing, we divided photo-ageing into its wrinkling constituent and its dyspigmentation constituent, as done by Chung *et al.* (2001) ^45^. - Solar elastosis appears as thickened elastic fibres and large amounts of granular and amorphous material, ending in a definite band of elastotic material ^63^. Favre-Racouchot syndrome manifested itself as yellowish and leathery skin with several flesh-coloured, white, or black cysts around the eyes ^28^. - Telangiectasia manifests itself as small, dilated, linear or branched-like, red or blue blood vessels visible on the skin of the nose or cheeks ^64^. |
| **Referenced Studies**  1. Wong QYA, Chew FT. Defining skin ageing and its risk factors: a systematic review and meta-analysis. *Sci Rep*. 2021;11(1):1-13. doi:10.1038/s41598-021-01573-z  2. Ng JY, Chew FT. A systematic review of skin ageing genes : gene pleiotropy and genes on the chromosomal band 16q24 . 3 may drive skin ageing. *Sci Rep*. Published online 2022:1-23. doi:10.1038/s41598-022-17443-1  3. Krutmann J, Bouloc A, Sore G, Bernard BA, Passeron T. The skin ageing exposome. *J Dermatol Sci*. 2017;85(3):152-161. doi:10.1016/j.jdermsci.2016.09.015  4. Hamer MA, Pardo LM, Jacobs LC, et al. Facial Wrinkles in Europeans: A Genome-Wide Association Study. *J Invest Dermatol*. 2018;138(8):1877-1880. doi:10.1016/j.jid.2017.12.037  5. Jacobs LC, Hamer MA, Gunn DA, et al. A Genome-Wide Association Study Identifies the Skin Color Genes IRF4 , MC1R , ASIP , and BNC2 Influencing Facial Pigmented Spots. *J Invest Dermatol*. 2015;135(7):1735-1742. doi:10.1038/jid.2015.62  6. Le Clerc S, Taing L, Ezzedine K, et al. A genome-wide association study in caucasian women points out a putative role of the STXBP5L gene in facial photoageing. *J Invest Dermatol*. 2013;133(4):929-935. doi:10.1038/jid.2012.458  7. Lim JJ, Lim YYE, Ng JY, et al. An update on the prevalence, chronicity, and severity of atopic dermatitis and the associated epidemiological risk factors in the Singapore/Malaysia Chinese young adult population: A detailed description of the Singapore/Malaysia Cross-Sectional Genetics . *World Allergy Organ J*. 2022;15(12):100722. doi:10.1016/j.waojou.2022.100722  8. Wong QYA, Lim JJ, Ng JY, et al. Allergic rhinitis in Chinese young adults from the Singapore/Malaysia cross-sectional genetics epidemiology study (SMCGES) cohort: Prevalence, patterns, and epidemiology of allergic rhinitis. *World Allergy Organ J*. 2022;15(10):100704. doi:10.1016/j.waojou.2022.100704  9. Wong QYA, Lim JJ, Ng JY, et al. An updated prevalence of asthma, its phenotypes, and the identification of the potential asthma risk factors among young Chinese adults recruited in Singapore. *World Allergy Organ J*. 2023;16(3):100757. doi:10.1016/j.waojou.2023.100757  10. Teo WY, Lim YYE, Sio YY, Say YH, Reginald K, Chew FT. Atopic dermatitis-associated genetic variants regulate LOC100294145 expression implicating interleukin-27 production and type 1 interferon signaling. *World Allergy Organ J*. 2024;17(2):100869. doi:10.1016/j.waojou.2023.100869  11. Sio YY, Gan WL, Ng WS, et al. The ERBB2 Exonic Variant Pro1170Ala Modulates Mitogen-Activated Protein Kinase Signaling Cascades and Associates with Allergic Asthma. *Int Arch Allergy Immunol*. 2023;184(10):1010-1021. doi:10.1159/000530960  12. Sio YY, Matta SA, Ng YT, Chew FT. Epistasis between phenylethanolamine N-methyltransferase and β2-adrenergic receptor influences extracellular epinephrine level and associates with the susceptibility to allergic asthma. *Clin Exp Allergy*. 2020;50(3):352-363. doi:10.1111/cea.13552  13. Sio YY, Shi P, Matta SA, et al. Functional Polymorphisms of the Arachidonic Acid Pathway Associate with Risks and Clinical Outcomes of Allergic Diseases. *Int Arch Allergy Immunol*. 2023;184(6):609-623. doi:10.1159/000530393  14. Sio YY, Shi P, Say YH, Chew FT. Functional variants in the chromosome 4q21 locus contribute to allergic rhinitis risk by modulating the expression of N-acylethanolamine acid amidase. *Clin Exp Allergy*. 2022;52(1):127-136. doi:10.1111/cea.13883  15. Ng JY, Zhou H, Li T, Chew FT. Comparisons between wrinkles and photo-ageing detected and self-reported by the participant or identified by trained assessors reveal insights from Chinese individuals in the Singapore/Malaysia Cross-sectional Genetics Epidemiology Study (SMCGES) cohort. *J Physiol Anthropol*. 2024;43(1):1-22. doi:10.1186/s40101-024-00361-8  16. Ng JY, Chew FT. Comparisons between Caucasian-validated photo-numeric scales and Korean-validated photo-numeric scales for photo-ageing. Insights from the Singapore/Malaysia cross-sectional genetics epidemiology study (SMCGES) cohort. *Ski Res Technol*. 2024;30(2):1-11. doi:10.1111/srt.13637  17. Ng JY, Chew FT. Comparisons between eyebags, droopy eyelids, and eyebrow positioning identified by photo-numeric scales or identified by written descriptive scales: Insights from the Singapore/Malaysia cross-sectional genetics epidemiology study (SMCGES) cohort. *Ski Res Technol*. 2024;30(2):1-12. doi:10.1111/srt.13620  18. Akoglu H. Turkish Journal of Emergency Medicine User ’ s guide to correlation coe ffi cients. 2018;18(August):91-93. doi:10.1016/j.tjem.2018.08.001  19. Chan YH. Correlation Analysis. *Singapore Med J*. 2003;44(12):614-619.  20. Braeken J, Van Assen MALM. An empirical Kaiser criterion. *Psychol Methods*. 2017;22(3):450-466. doi:10.1037/met0000074  21. Guinot C, Malvy DJM, Ambroisine L, et al. Relative Contribution of Intrinsic vs Extrinsic Factors to Skin Ageing as Determined by a Validated Skin Age Score. *Arch Dermatol*. 2002;138(11). doi:10.1001/archderm.138.11.1454  22. Buranasirin P, Pongpirul K, Meephansan J. Development of a Global Subjective Skin Ageing Assessment score from the perspective of dermatologists. *BMC Res Notes*. 2019;12(1). doi:10.1186/s13104-019-4404-z  23. Vierkötter A, Ranft U, Krämer U, Sugiri D, Reimann V, Krutmann J. The SCINEXA: A novel, validated score to simultaneously assess and differentiate between intrinsic and extrinsic skin ageing. *J Dermatol Sci*. 2009;53(3):207-211. doi:10.1016/j.jdermsci.2008.10.001  24. Sextius P, Marionnet C, Tacheau C, et al. Analysis of gene expression dynamics revealed delayed and abnormal epidermal repair process in aged compared to young skin. *Arch Dermatol Res*. 2015;307(4):351-364. doi:10.1007/s00403-015-1551-5  25. Zhang M, Li B, Wu S, et al. A Genome-Wide Association Study of Basal Transepidermal Water Loss Finds that Variants at 9q34.3 Are Associated with Skin Barrier Function. *J Invest Dermatol*. 2017;137(4):979-982. doi:10.1016/j.jid.2016.11.030  26. Azizi E, Lusky A, Kushelevsky AP, Schewach-Millet M. Skin type, hair colour, and freckles are predictors of decreased minimal erythema ultraviolet radiation dose. *J Am Acad Dermatol*. 1988;19(1):32-38. doi:10.1016/S0190-9622(88)70148-6  27. Swift A, Liew S, Weinkle S, Garcia JK, Silberberg MB. The Facial Ageing Process from the “Inside Out.” *Aesthetic Surg J*. 2021;41(10):1107-1119. doi:10.1093/asj/sjaa339  28. Goldsmith LA, Katz SI, Gilchrest BA, Paller AS, Leffell DJ, Wolff K. *Fitzpatrick’s Dermatology in General Medicine, 8th Edition*. 8th ed. The McGraw-Hill Companies, Inc; 2011.  29. Habif TP, Dinulos JGH, Chapman MS, Zug KA. *Skin Disease Diagnosis and Treatment*. 3rd ed. Elsevier; 2018.  30. Rattner H. *Color Atlas of Skin Diseases*.; 1962.  31. Norman RA. *Diagnosis of Ageing Skin Diseases*. Springer-Verlag London Limited; 2008.  32. Flament, Frederic & Bazin R. *Skin Ageing Atlas Volume 2: Asian Type*.; 2010.  33. Chen Y, André M, Adhikari K, et al. A genome-wide association study identifies novel gene associations with facial skin wrinkling and mole count in Latin Americans. *Br J Dermatol*. 2021;185(5):988-998. doi:10.1111/bjd.20436  34. Bolognia J, Jorizzo J, Schaffer J, et al. *Dermatology, 3rd Edition*. Elsevier; 2012.  35. Tsukahara K, Takema Y, Kazama H, et al. A photographic scale for the assessment of human facial wrinkles. *J Cosmet Sci*. 2000;51(2):127-139.  36. Ferri FF, Studdiford JS, Tully A. *Ferri’s Fast Facts in Dermatology*. Saunders Elsevier; 2011.  37. Ezure T, Yagi E, Kunizawa N, Hirao T, Amano S. Comparison of sagging at the cheek and lower eyelid between male and female faces. 2011;17(4):510-515. doi:10.1111/j.1600-0846.2011.00526.x  38. Flynn TC, Carruthers A, Carruthers J, et al. Validated Assessment Scales for the Upper Face. *Dermatologic Surg*. 2012;38(2ptII):309-319. doi:10.1111/j.1524-4725.2011.02248.x  39. Honeck P, Weiss C, Sterry W, Rzany B. Reproducibility of a four-point clinical severity score for glabellar frown lines. *Br J Dermatol*. 2003;149(2):306-310. doi:10.1046/j.1365-2133.2003.05436.x  40. Nakashima Y, Wada K, Yamakawa M, Nagata C. Validity of self‐reported skin colour by using skin colour evaluation scale. *Ski Res Technol*. 2022;28(6):827-832. doi:10.1111/srt.13207  41. Carruthers A, Carruthers J, Hardas B, et al. A Validated Brow Positioning Grading Scale. *Am Soc Dermatologic Surg*. 2008;34:S150-S154. doi:10.1111/j.1524-4725.2008.34363.x  42. Wolff K, Johnson RA, Saavedra AP, Roh EK. *Fitzpatrick’s Color Atlas and Synopsis of Clinical Dermatology*. 8th ed. McGraw-Hill Education; 2017.  43. Narins RS, Carruthers J, Flynn TC, et al. Validated Assessment Scales for the Lower Face. *Dermatologic Surg*. 2012;38(2ptII):333-342. doi:10.1111/j.1524-4725.2011.02247.x  44. Larnier C, Ortonne J ‐P, Venot A, et al. Evaluation of cutaneous photodamage using a photographic scale. *Br J Dermatol*. 1994;130(2):167-173. doi:10.1111/j.1365-2133.1994.tb02895.x  45. Chung JH, Lee SH, Youn CS, et al. Cutaneous photodamage in Koreans. *J Dermatol*. 2001;28(11):614-616. doi:10.1111/j.1346-8138.2001.tb00045.x  46. Liu Y, Gao W, Koellmann C, et al. Genome-wide scan identified genetic variants associated with skin ageing in a Chinese female population. *J Dermatol Sci*. 2019;96(1):42-49. doi:10.1016/j.jdermsci.2019.08.010  47. Goldberg RA, Lew H. Cosmetic outcome of posterior approach ptosis surgery. *Trans Am Ophthalmol Soc*. 2011;109:157-167. Accessed September 8, 2020. https://www.ncbi.nlm.nih.gov/pmc/articles/PMC3259674/  48. Lorenc ZP, Bank D, Kane M, Lin X, Smith S. Validation of a Four-Point Photographic Scale for the Assessment of Midface Volume Loss and/or Contour Deficiency. *Plast Reconstr Surg*. 2012;130(6):1330-1336. doi:10.1097/prs.0b013e31826d9fa6  49. Sattler G, Carruthers A, Carruthers J, et al. Validated Assessment Scale for Neck Volume. *Dermatologic Surg*. 2012;38(2ptII):343-350. doi:10.1111/j.1524-4725.2011.02253.x  50. Marks R. *Roxburgh’s Common Skin Diseases*. 17th ed. Arnold Publishers; 2003.  51. Jacobs LC, Liu F, Bleyen I, et al. Intrinsic and extrinsic risk factors for sagging eyelids. *JAMA Dermatology*. 2014;150(8):836-843. doi:10.1001/jamadermatol.2014.27  52. Endo C, Johnson TA, Morino R, et al. Genome-wide association study in Japanese females identifies fifteen novel skin-related trait associations. *Sci Rep*. 2018;8(8974). doi:10.1038/s41598-018-27145-2  53. Motokawa T, Kato T, Hashimoto Y, Takimoto H, Yamamoto H, Katagiri T. Polymorphism patterns in the promoter region of the MC1R gene are associated with development of freckles and solar lentigines. *J Invest Dermatol*. 2008;128(6):1588-1591. doi:10.1038/sj.jid.5701192  54. Xu X, Thörnwall M, Lundin LG, Chhajlani V. Val92met variant of the melanocyte stimulating hormone receptor gene. *Nat Genet*. 1996;14(4):384. doi:10.1038/ng1296-384  55. Courtney H. Senile lentigo. *Arch Dermatol*. Published online 1963.  56. Bastiaens M, Jeanette ter H, Nelleke G, et al. The melanocortin-1-receptor gene is the major freckle gene. *Hum Mol Genet*. 2001;10(16):1701-1708. doi:10.1093/hmg/10.16.1701  57. Laville V, Clerc S Le, Ezzedine K, et al. A Genome-wide association study in Caucasian women suggests the involvement of HLA genes in the severity of facial solar lentigines. *Pigment Cell Melanoma Res*. 2016;29(5):550-558. doi:10.1111/pcmr.12502  58. Shin JG, Leem S, Kim B, et al. GWAS Analysis of 17,019 Korean Women Identifies the Variants Associated with Facial Pigmented Spots. *J Invest Dermatol*. 2021;141(3):555-562. doi:10.1016/j.jid.2020.08.007  59. Ezzedine K, Mauger E, Latreille J, et al. Freckles and solar lentigines have different risk factors in Caucasian women. *J Eur Acad Dermatology Venereol*. 2012;27(3):e345-e356. doi:10.1111/j.1468-3083.2012.04685.x  60. Prose NS, Kristal L. *Weinberg’s Color Atlas of Pediatric Dermatology*. 5th ed. (Edmonson KG, Pancotti R, eds.). McGraw-Hill Education; 2017.  61. Jin Ho C, Seong Hun L, Choon Shik Y, et al. Cutaneous photodamage in Koreans. *J Dermatol*. 2001;28(11):614-616. doi:10.1111/j.1346-8138.2001.tb00045.x  62. Griffiths CEM, Wang TS, Hamilton TA, Voorhees JJ, Ellis CN. A Photonumeric Scale for the Assessment of Cutaneous Photodamage. *Arch Dermatol*. 1992;128(10):1406. doi:10.1001/archderm.1992.01680200118026  63. Law MH, Medland SE, Zhu G, et al. Genome-Wide Association Shows that Pigmentation Genes Play a Role in Skin Ageing. *J Invest Dermatol*. 2017;137(9):1887-1894. doi:10.1016/j.jid.2017.04.026  64. Mekić S, Wigmann C, Gunn DA, et al. Genetics of facial telangiectasia in the Rotterdam Study: a genome-wide association study and candidate gene approach. *J Eur Acad Dermatology Venereol*. 2021;35(3):749-754. doi:10.1111/jdv.17014 | |
