# Supplementary material for: A broad assessment of forty-one skin phenotypes reveals complex dimensions of skin ageing
Source: J Physiol Anthropol. 2025 Feb 8;44:3. doi: 10.1186/s40101-024-00383-2 (PMC11806859; doi:10.1186/s40101-024-00383-2)
Supplement: Supplementary file 2 — Additional file 2. Total variance explained by each of the forty-one principal components (PCs). [file 40101_2024_383_MOESM2_ESM.docx]

**Additional file 2:** Total variance explained by each of the forty-one principal components (PCs).

| **Component** | **Initial eigenvalues** | | |
| --- | --- | --- | --- |
|  | **Total** | **Percentage of variance** | **Cumulative Percentage** |
| 1 | 7.12 | 17.36 | 17.36 |
| 2 | 2.88 | 7.04 | 24.39 |
| 3 | 1.43 | 3.49 | 27.88 |
| 4 | 1.33 | 3.24 | 31.12 |
| 5 | 1.18 | 2.89 | 34.01 |
| 6 | 1.15 | 2.79 | 36.80 |
| 7 | 1.08 | 2.63 | 39.43 |
| 8 | 1.04 | 2.53 | 41.96 |
| 9 | 1.02 | 2.48 | 44.43 |
| 10 | 1.00 | 2.45 | 46.88 |
| 11 | 0.97 | 2.37 | 49.25 |
| 12 | 0.96 | 2.34 | 51.59 |
| 13 | 0.96 | 2.33 | 53.93 |
| 14 | 0.93 | 2.26 | 56.19 |
| 15 | 0.92 | 2.24 | 58.43 |
| 16 | 0.91 | 2.21 | 60.64 |
| 17 | 0.88 | 2.14 | 62.78 |
| 18 | 0.86 | 2.09 | 64.87 |
| 19 | 0.83 | 2.02 | 66.89 |
| 20 | 0.81 | 1.98 | 68.87 |
| 21 | 0.80 | 1.95 | 70.82 |
| 22 | 0.78 | 1.90 | 72.72 |
| 23 | 0.77 | 1.87 | 74.59 |
| 24 | 0.77 | 1.87 | 76.46 |
| 25 | 0.75 | 1.83 | 78.29 |
| 26 | 0.71 | 1.74 | 80.03 |
| 27 | 0.69 | 1.68 | 81.71 |
| 28 | 0.68 | 1.66 | 83.37 |
| 29 | 0.66 | 1.62 | 84.99 |
| 30 | 0.63 | 1.54 | 86.52 |
| 31 | 0.60 | 1.47 | 87.99 |
| 32 | 0.60 | 1.46 | 89.45 |
| 33 | 0.59 | 1.43 | 90.88 |
| 34 | 0.57 | 1.38 | 92.26 |
| 35 | 0.54 | 1.32 | 93.58 |
| 36 | 0.51 | 1.23 | 94.81 |
| 37 | 0.49 | 1.18 | 96.00 |
| 38 | 0.45 | 1.11 | 97.11 |
| 39 | 0.42 | 1.03 | 98.14 |
| 40 | 0.39 | 0.95 | 99.09 |
| 41 | 0.37 | 0.91 | 100.00 |
