# Supplementary figures and images for: A broad assessment of forty-one skin phenotypes reveals complex dimensions of skin ageing
Source: J Physiol Anthropol. 2025 Feb 8;44:3. doi: 10.1186/s40101-024-00383-2 (PMC11806859; doi:10.1186/s40101-024-00383-2)

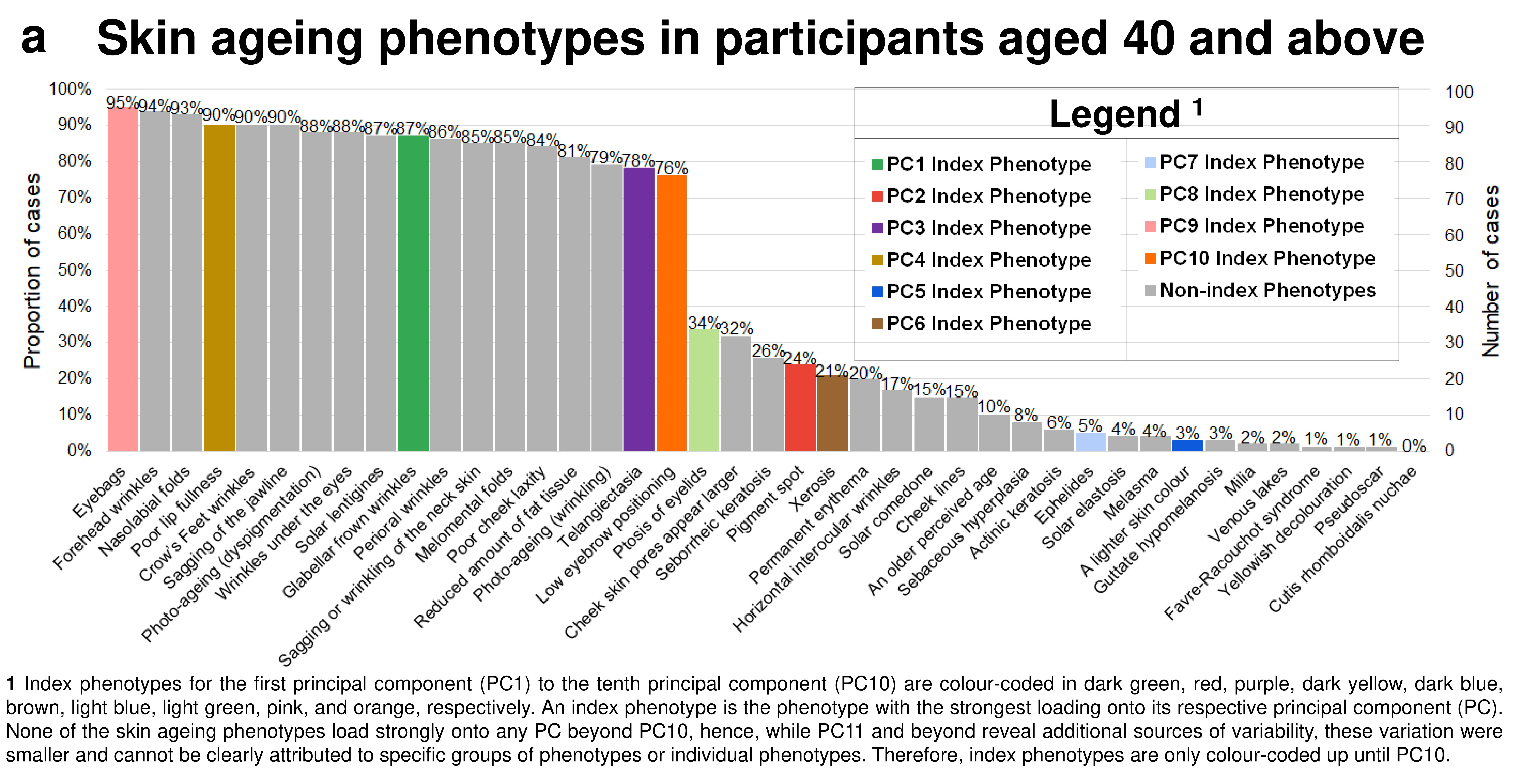

Supplement: Supplementary file 3 — Additional file 3. Prevalence of forty-one skin ageing phenotypes from ethnic Chinese participants aged 40 years and above from the Singapore/Malaysia Cross-sectional Genetics Epidemiology Study (SMCGES) (n=101). [file 40101_2024_383_MOESM3_ESM.png]

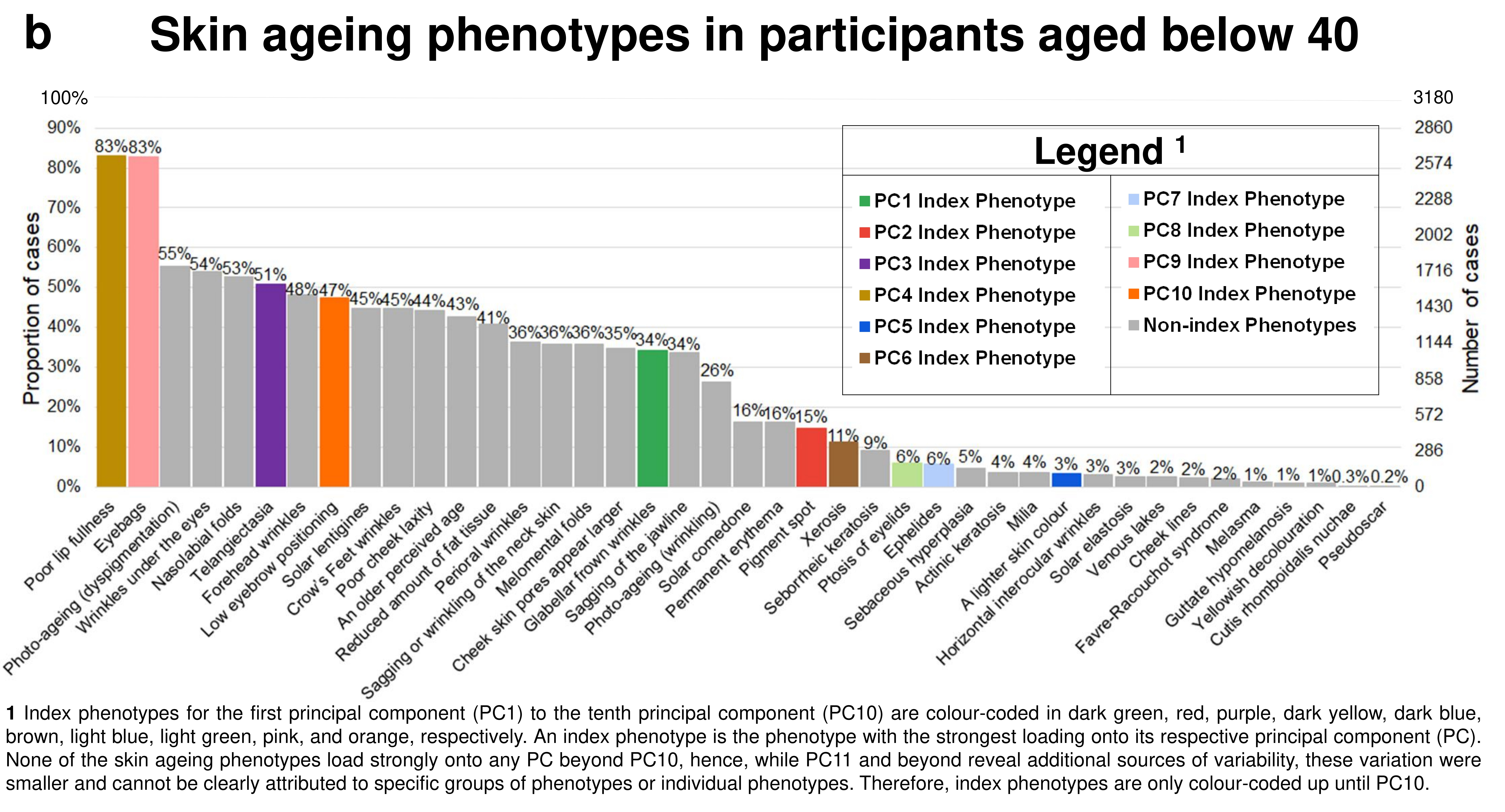

Supplement: Supplementary file 4 — Additional file 4. Prevalence of forty-one skin ageing phenotypes from ethnic Chinese participants aged below 40 from the Singapore/Malaysia Cross-sectional Genetics Epidemiology Study (SMCGES) (n=3180). [file 40101_2024_383_MOESM4_ESM.png]

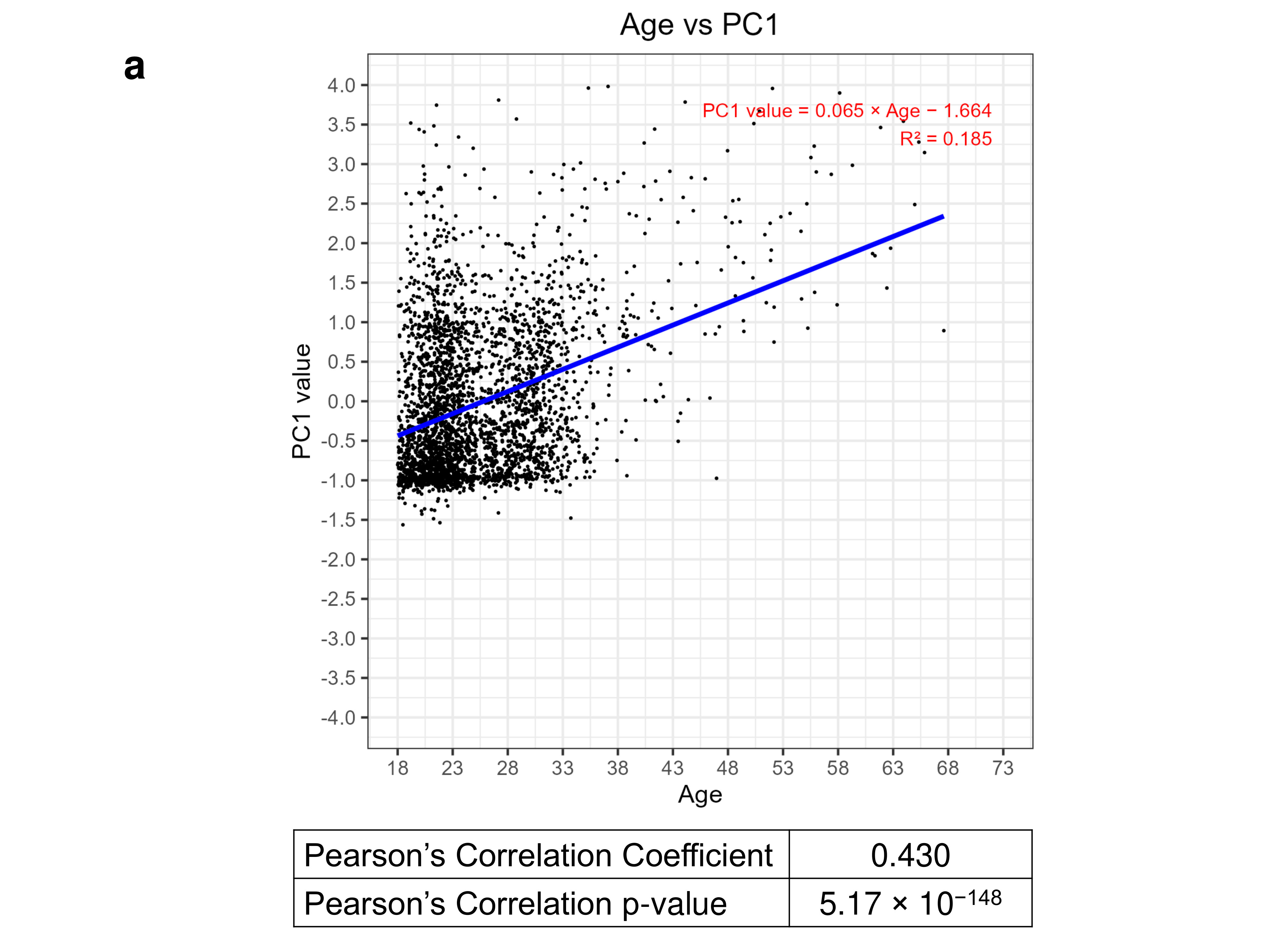

Supplement: Supplementary file 5 — Additional file 5. Correlation between chronological age and PC1 values. Pearson’s correlation coefficients are computed for each plot. p-values reported are two-tailed Pearson’s correlation p-values. A line of goodness of fit is included, based on a linear regression model, with the R² coefficient of determination displayed for each plot. [file 40101_2024_383_MOESM5_ESM.png]

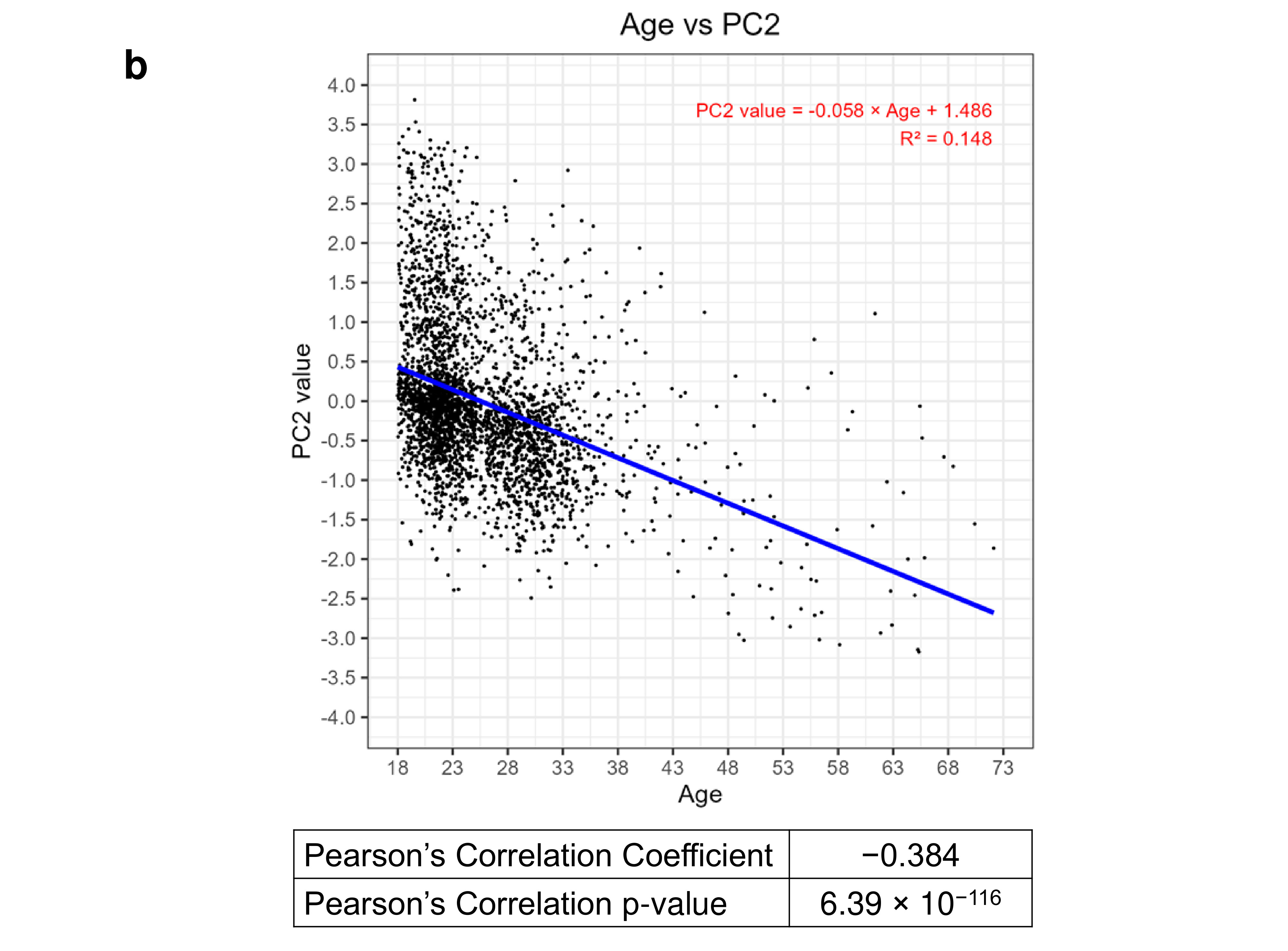

Supplement: Supplementary file 6 — Additional file 6. Correlation between chronological age and PC2 values. Pearson’s correlation coefficients are computed for each plot. p-values reported are two-tailed Pearson’s correlation p-values. A line of goodness of fit is included, based on a linear regression model, with the R² coefficient of determination displayed for each plot. [file 40101_2024_383_MOESM6_ESM.png]

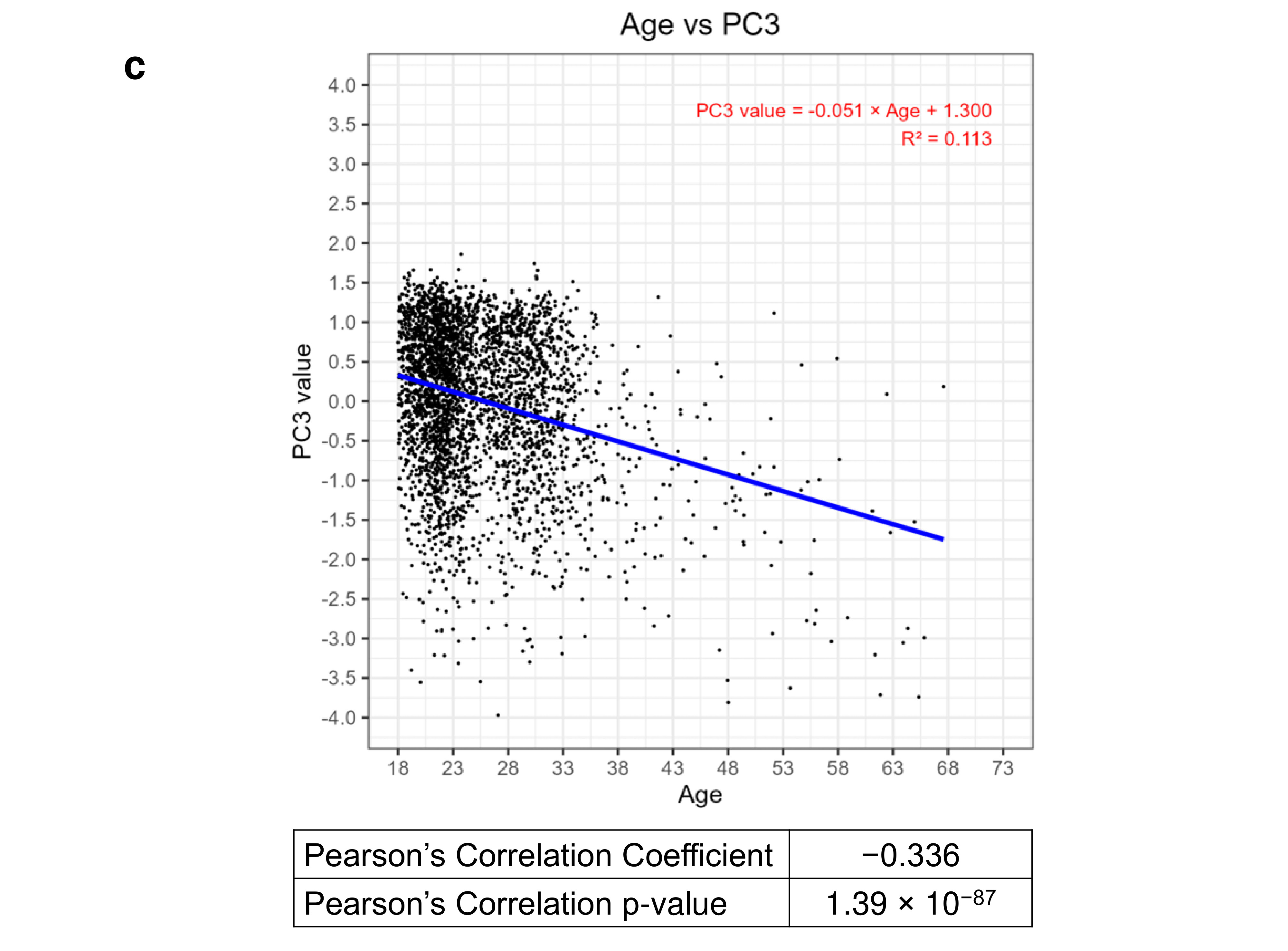

Supplement: Supplementary file 7 — Additional file 7. Correlation between chronological age and PC3 values. Pearson’s correlation coefficients are computed for each plot. p-values reported are two-tailed Pearson’s correlation p-values. A line of goodness of fit is included, based on a linear regression model, with the R² coefficient of determination displayed for each plot. [file 40101_2024_383_MOESM7_ESM.png]

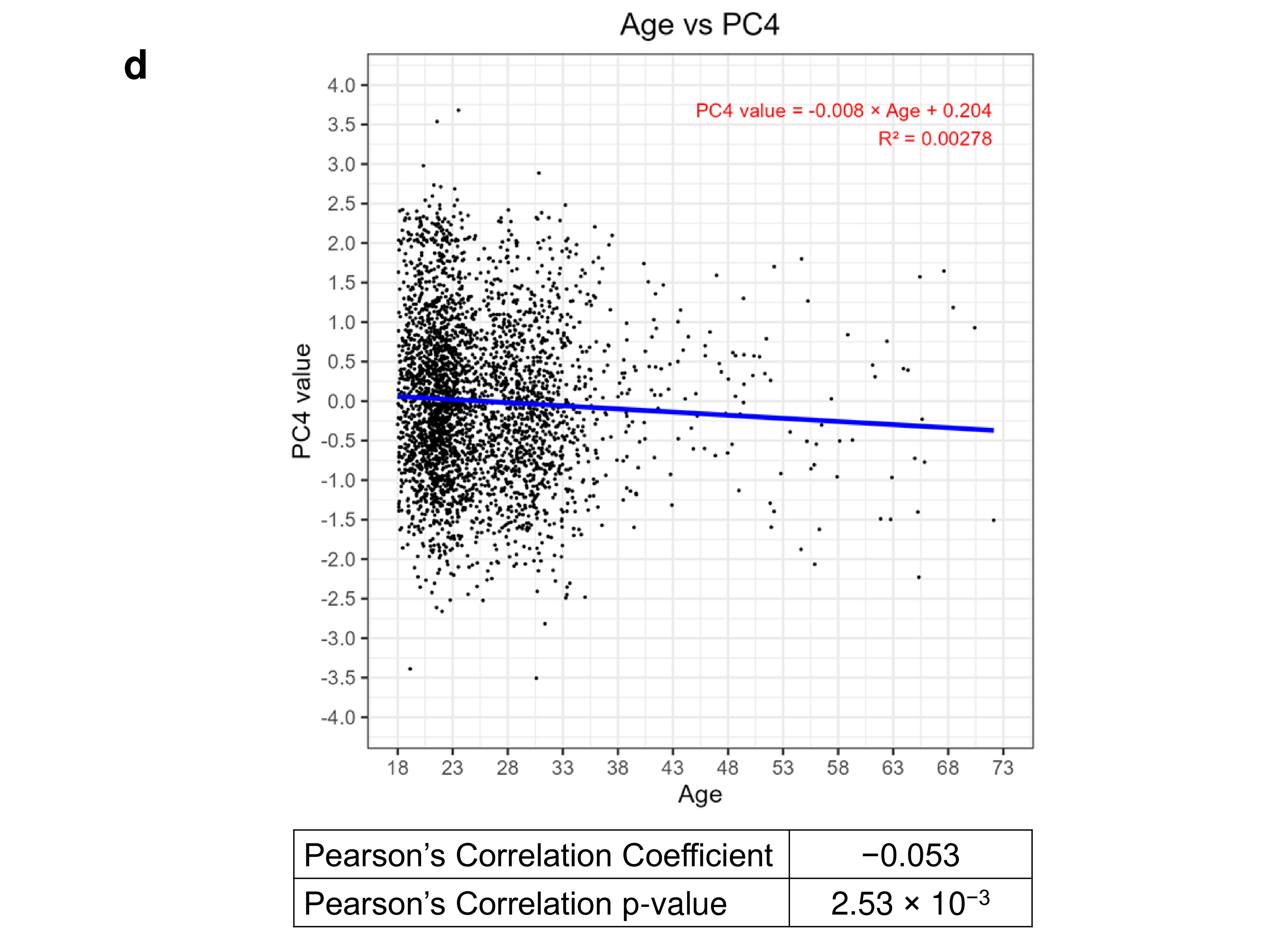

Supplement: Supplementary file 8 — Additional file 8. Correlation between chronological age and PC4 values. Pearson’s correlation coefficients are computed for each plot. p-values reported are two-tailed Pearson’s correlation p-values. A line of goodness of fit is included, based on a linear regression model, with the R² coefficient of determination displayed for each plot. [file 40101_2024_383_MOESM8_ESM.png]

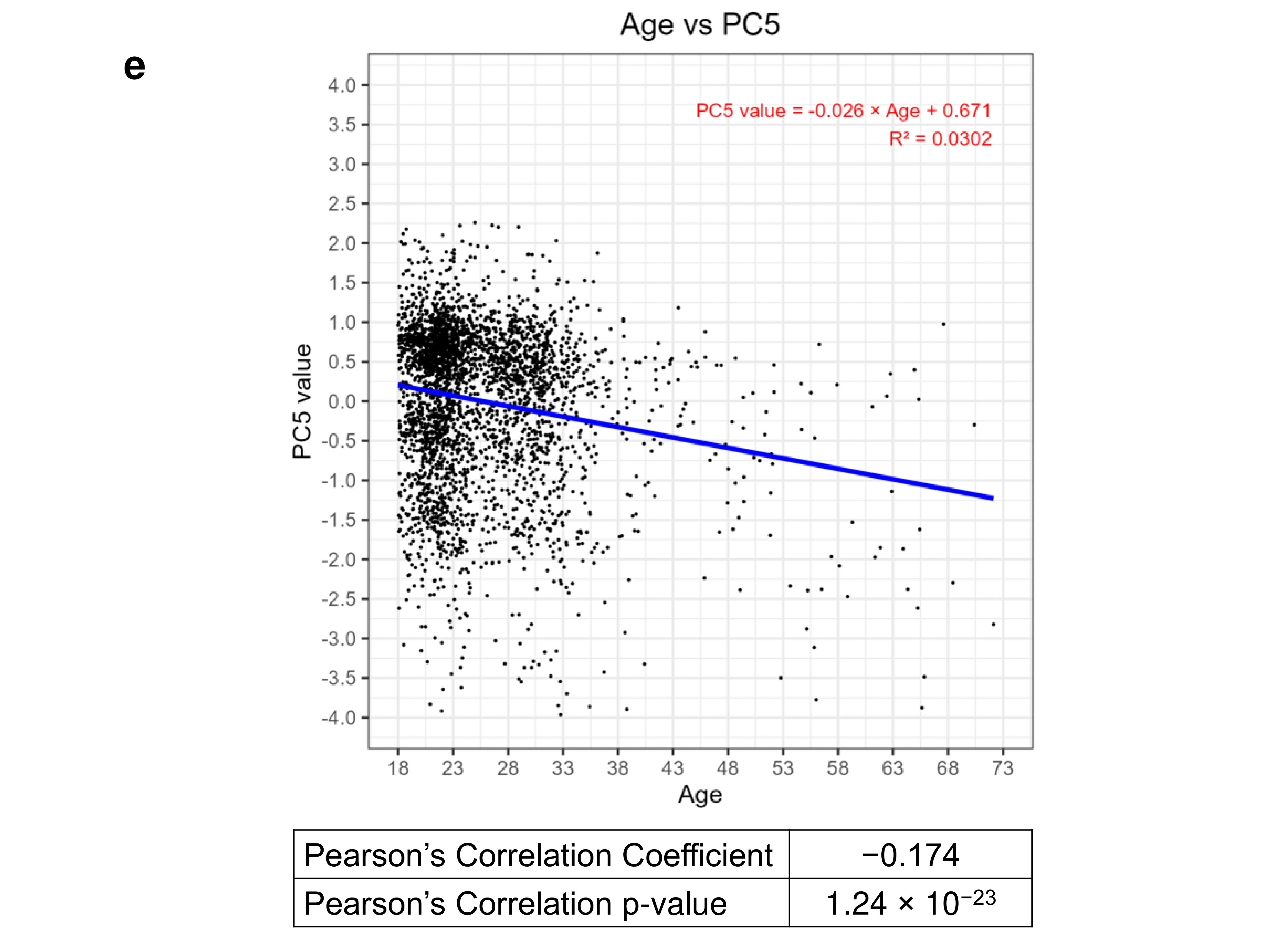

Supplement: Supplementary file 9 — Additional file 9. Correlation between chronological age and PC5 values. Pearson’s correlation coefficients are computed for each plot. p-values reported are two-tailed Pearson’s correlation p-values. A line of goodness of fit is included, based on a linear regression model, with the R² coefficient of determination displayed for each plot. [file 40101_2024_383_MOESM9_ESM.png]

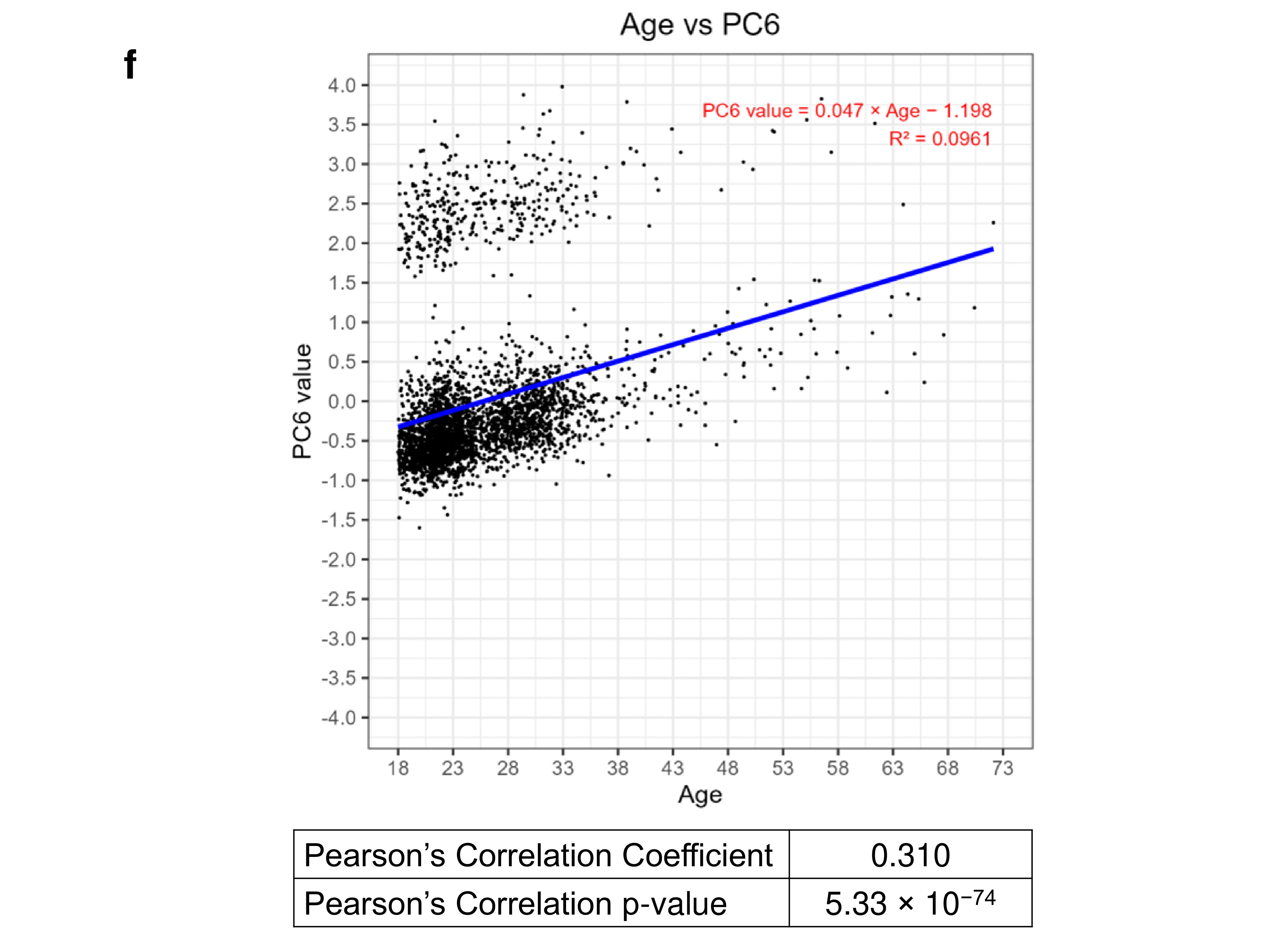

Supplement: Supplementary file 10 — Additional file 10. Correlation between chronological age and PC6 values. Pearson’s correlation coefficients are computed for each plot. p-values reported are two-tailed Pearson’s correlation p-values. A line of goodness of fit is included, based on a linear regression model, with the R² coefficient of determination displayed for each plot. [file 40101_2024_383_MOESM10_ESM.png]

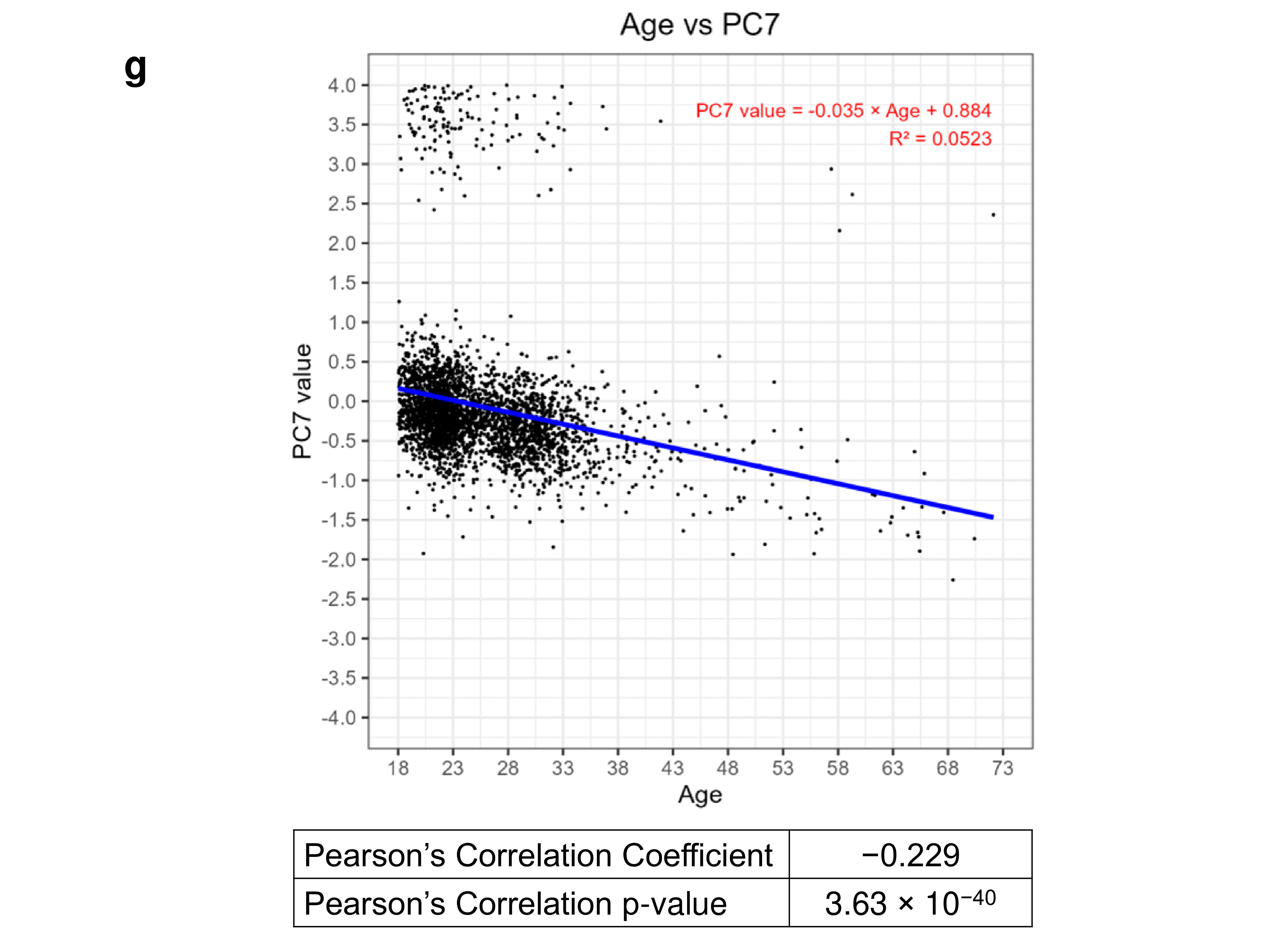

Supplement: Supplementary file 11 — Additional file 11. Correlation between chronological age and PC7 values. Pearson’s correlation coefficients are computed for each plot. p-values reported are two-tailed Pearson’s correlation p-values. A line of goodness of fit is included, based on a linear regression model, with the R² coefficient of determination displayed for each plot. [file 40101_2024_383_MOESM11_ESM.png]

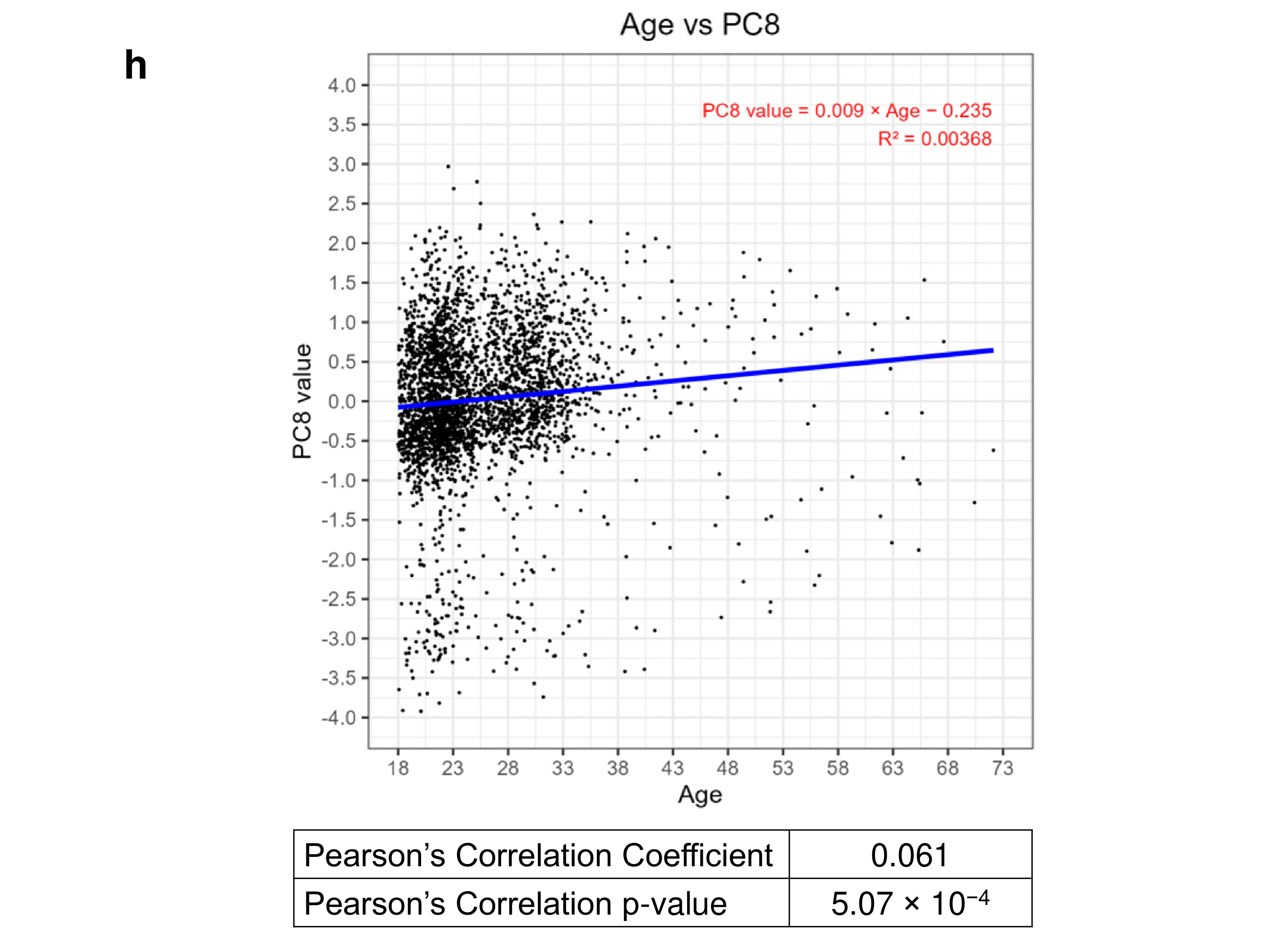

Supplement: Supplementary file 12 — Additional file 12. Correlation between chronological age and PC8 values. Pearson’s correlation coefficients are computed for each plot. p-values reported are two-tailed Pearson’s correlation p-values. A line of goodness of fit is included, based on a linear regression model, with the R² coefficient of determination displayed for each plot. [file 40101_2024_383_MOESM12_ESM.png]

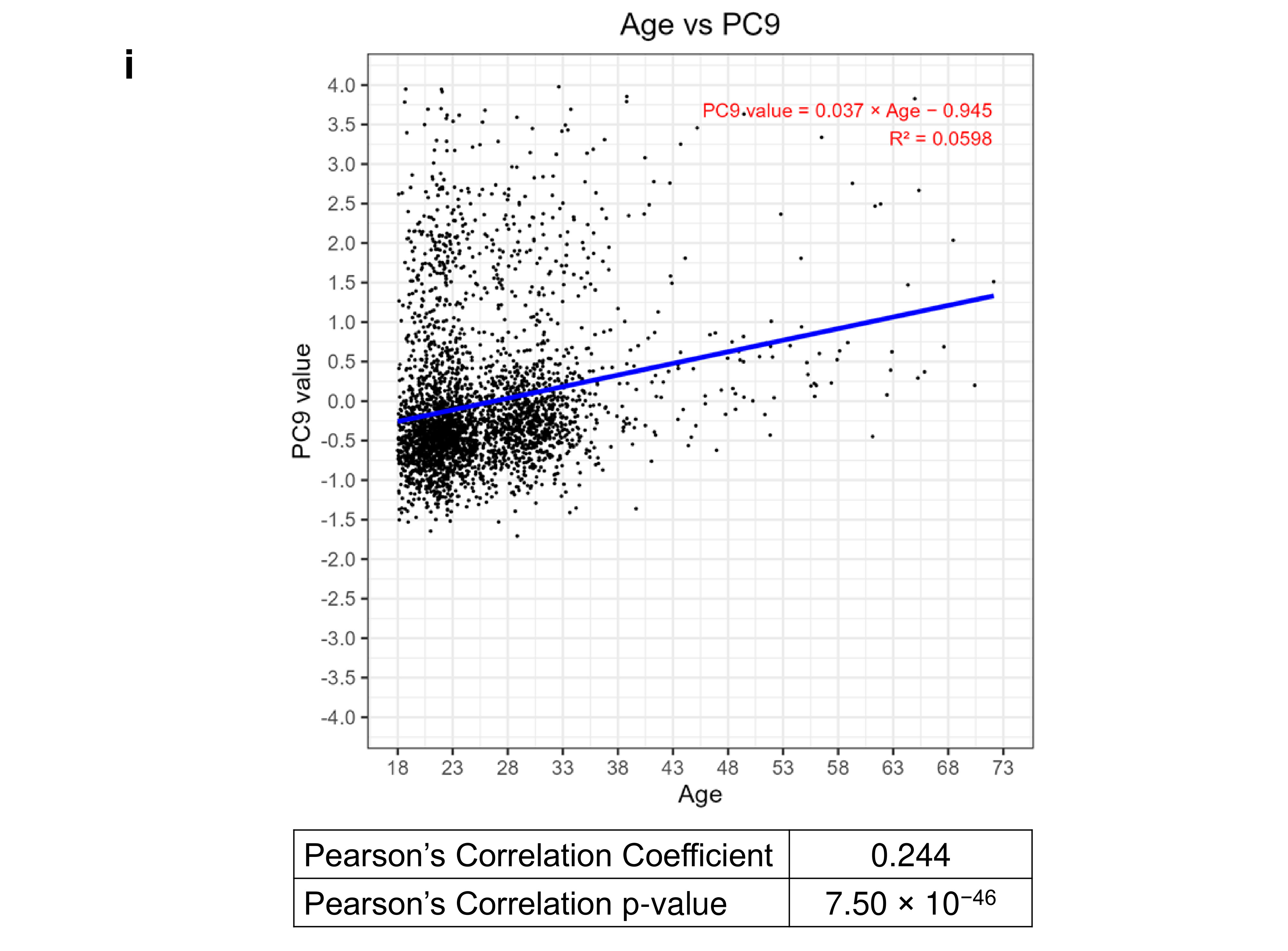

Supplement: Supplementary file 13 — Additional file 13. Correlation between chronological age and PC9 values. Pearson’s correlation coefficients are computed for each plot. p-values reported are two-tailed Pearson’s correlation p-values. A line of goodness of fit is included, based on a linear regression model, with the R² coefficient of determination displayed for each plot. [file 40101_2024_383_MOESM13_ESM.png]

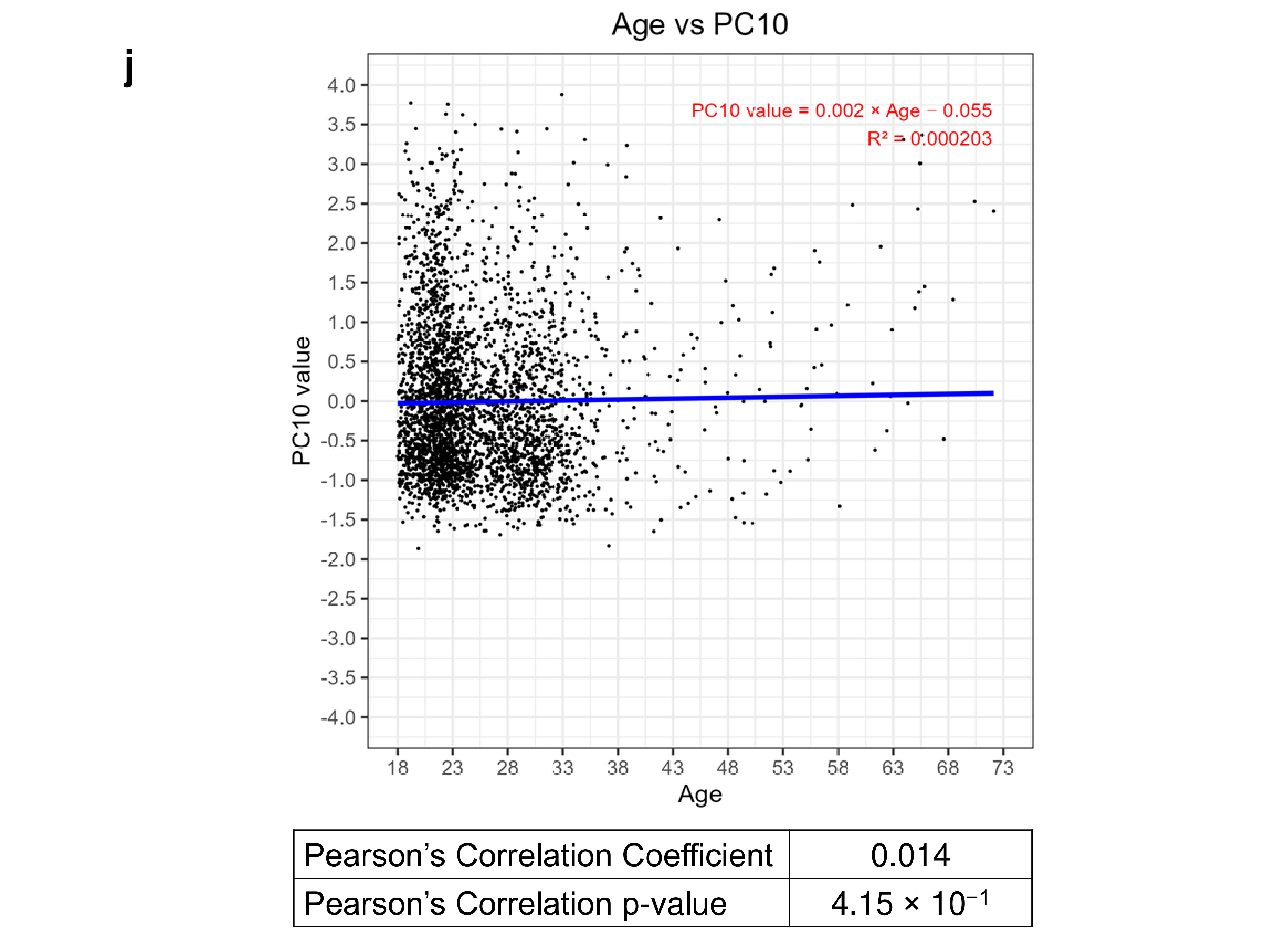

Supplement: Supplementary file 14 — Additional file 14. Correlation between chronological age and PC10 values. Pearson’s correlation coefficients are computed for each plot. p-values reported are two-tailed Pearson’s correlation p-values. A line of goodness of fit is included, based on a linear regression model, with the R² coefficient of determination displayed for each plot. [file 40101_2024_383_MOESM14_ESM.png]

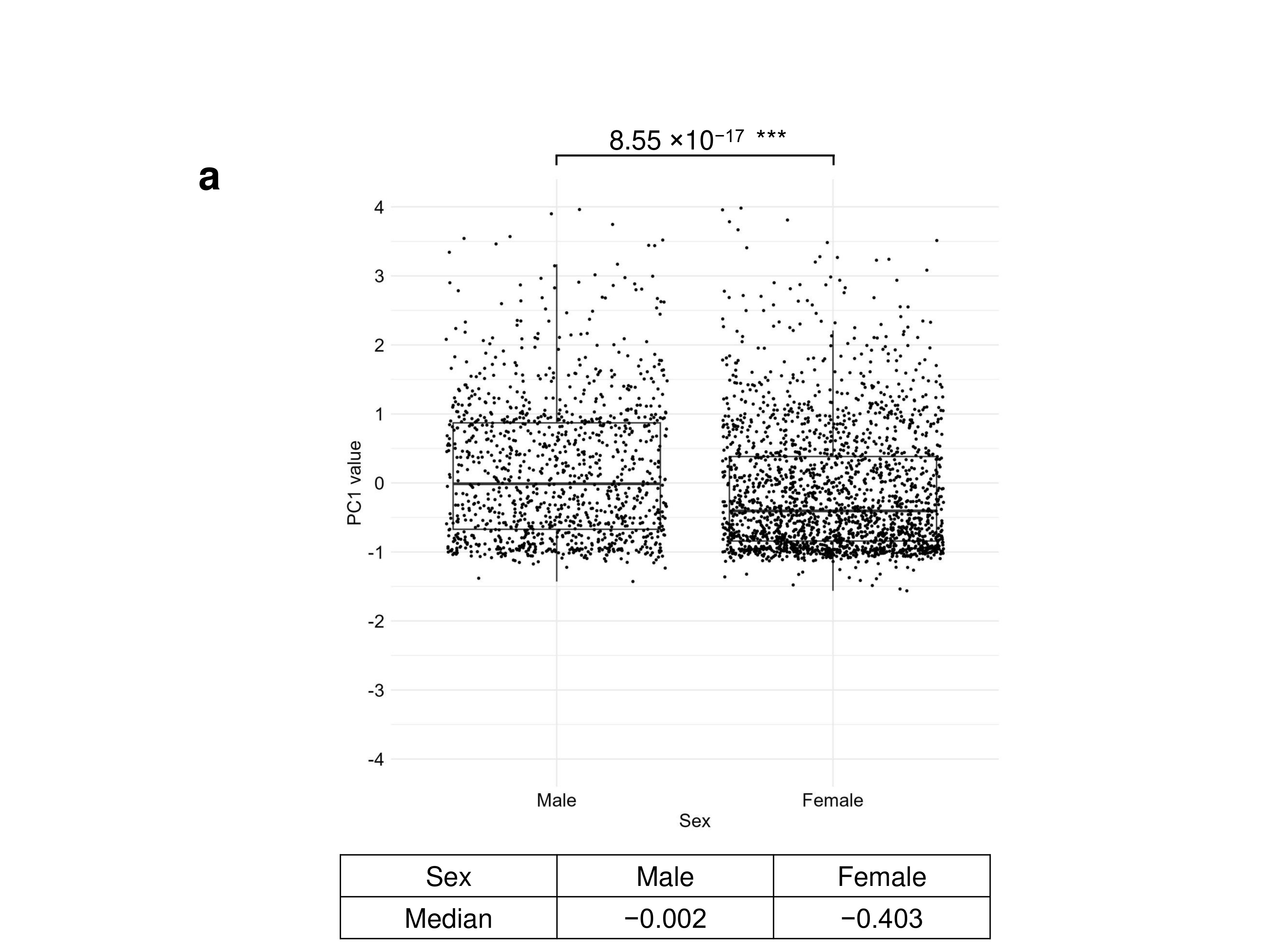

Supplement: Supplementary file 15 — Additional file 15. PC1 values stratified by sex. Two-tailed t-test p-values are computed for each plot. The median PC values for both sexes are displayed in each plot. p-values reported are two-tailed t-test p-values, with * indicating p < 0.05, ** p < 0.01, and *** p < 0.001. p > was considered statistically non-significant (ns). [file 40101_2024_383_MOESM15_ESM.png]

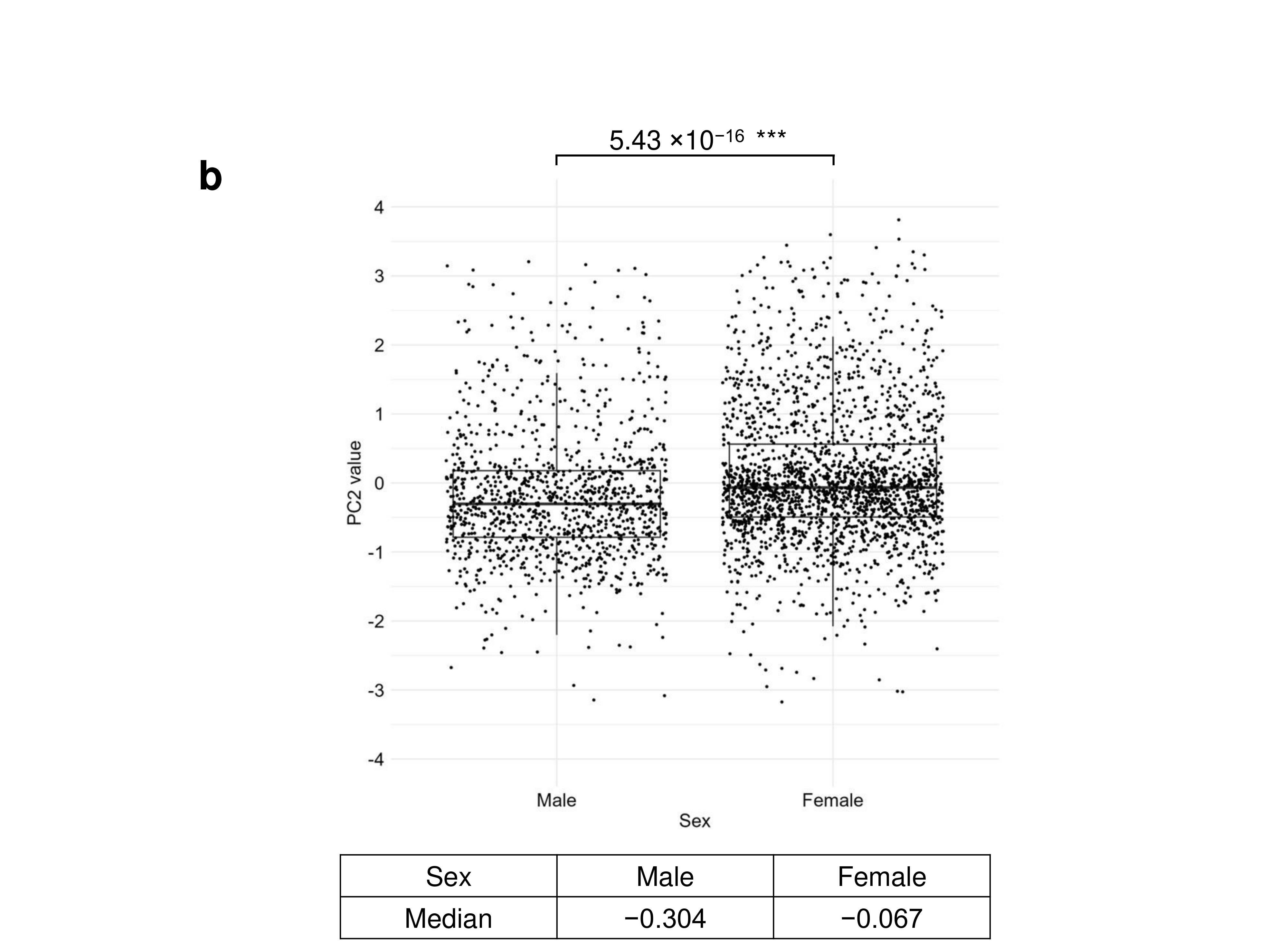

Supplement: Supplementary file 16 — Additional file 16. PC2 values stratified by sex. Two-tailed t-test p-values are computed for each plot. The median PC values for both sexes are displayed in each plot. p-values reported are two-tailed t-test p-values, with * indicating p < 0.05, ** p < 0.01, and *** p < 0.001. p > was considered statistically non-significant (ns). [file 40101_2024_383_MOESM16_ESM.png]

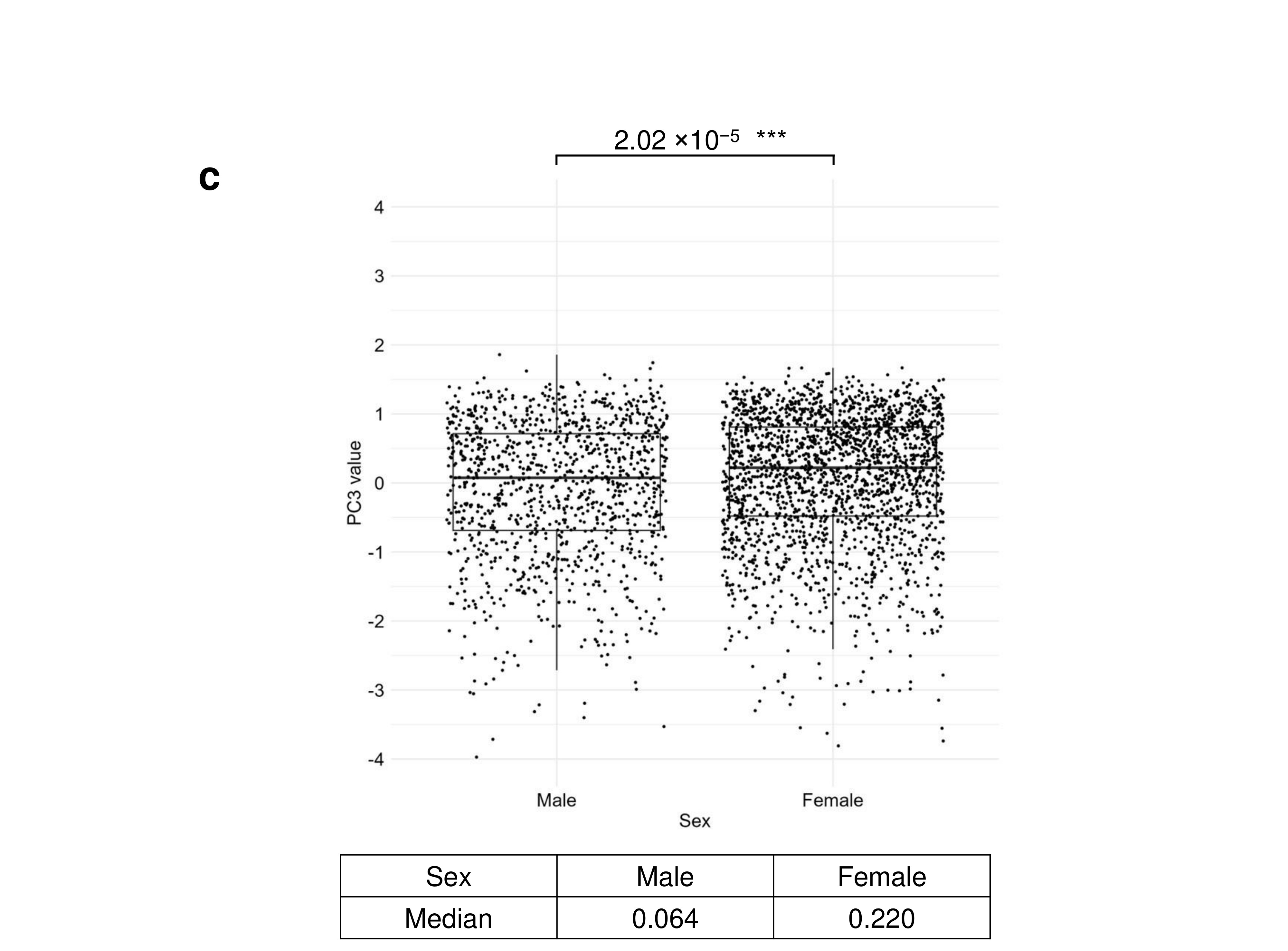

Supplement: Supplementary file 17 — Additional file 17. PC3 values stratified by sex. Two-tailed t-test p-values are computed for each plot. The median PC values for both sexes are displayed in each plot. p-values reported are two-tailed t-test p-values, with * indicating p < 0.05, ** p < 0.01, and *** p < 0.001. p > was considered statistically non-significant (ns). [file 40101_2024_383_MOESM17_ESM.png]

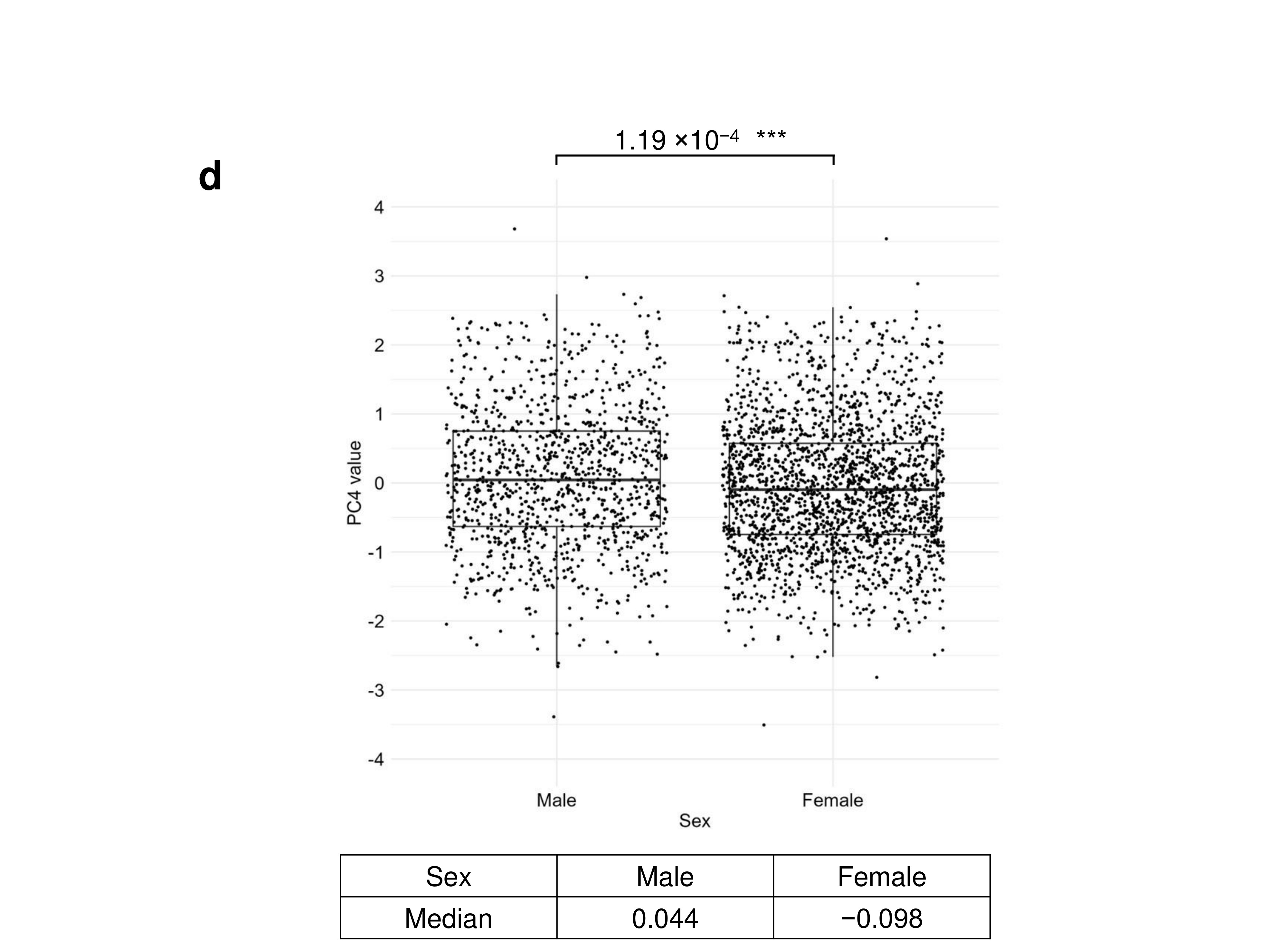

Supplement: Supplementary file 18 — Additional file 18. PC4 values stratified by sex. Two-tailed t-test p-values are computed for each plot. The median PC values for both sexes are displayed in each plot. p -values reported are two-tailed t-test p -values, with * indicating p < 0.05, ** p < 0.01, and *** p < 0.001. p > was considered statistically non-significant (ns). [file 40101_2024_383_MOESM18_ESM.png]

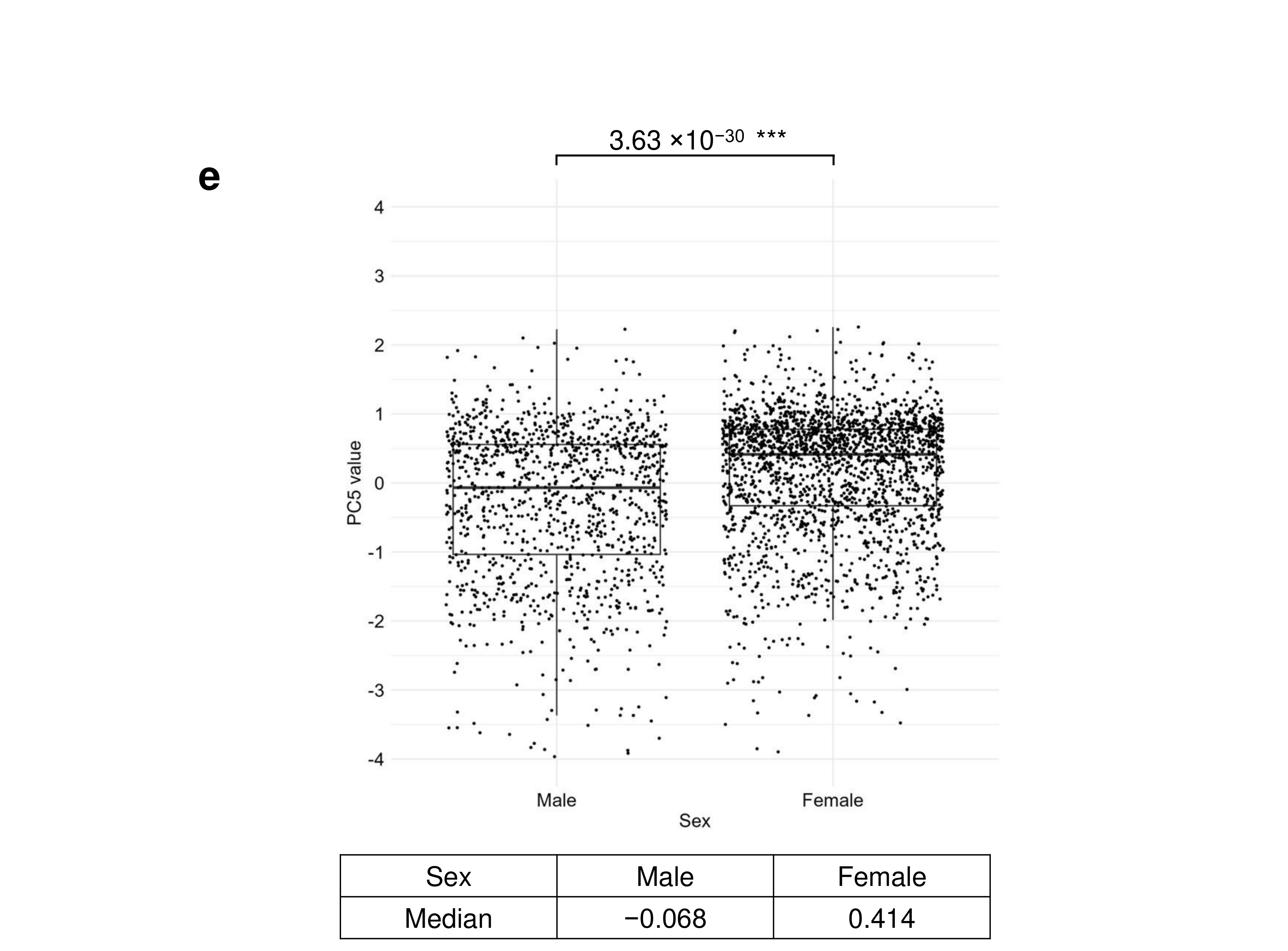

Supplement: Supplementary file 19 — Additional file 19. PC5 values stratified by sex. Two-tailed t-test p-values are computed for each plot. The median PC values for both sexes are displayed in each plot. p-values reported are two-tailed t-test p-values, with * indicating p < 0.05, ** p < 0.01, and *** p < 0.001. p > was considered statistically non-significant (ns). [file 40101_2024_383_MOESM19_ESM.png]

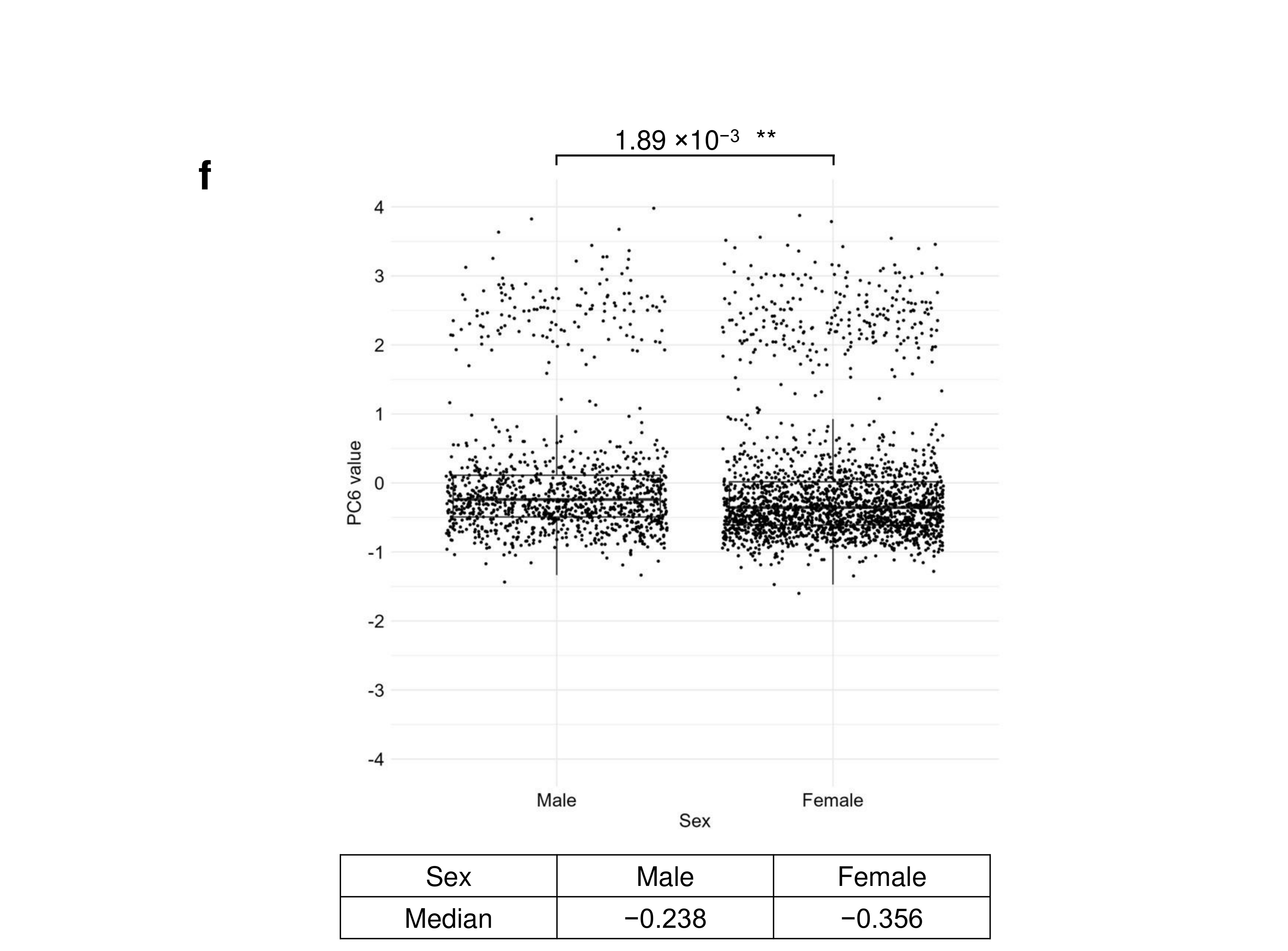

Supplement: Supplementary file 20 — Additional file 20. PC6 values stratified by sex. Two-tailed t-test p-values are computed for each plot. The median PC values for both sexes are displayed in each plot. p-values reported are two-tailed t-test p-values, with * indicating p < 0.05, ** p < 0.01, and *** p < 0.001. p > was considered statistically non-significant (ns). [file 40101_2024_383_MOESM20_ESM.png]

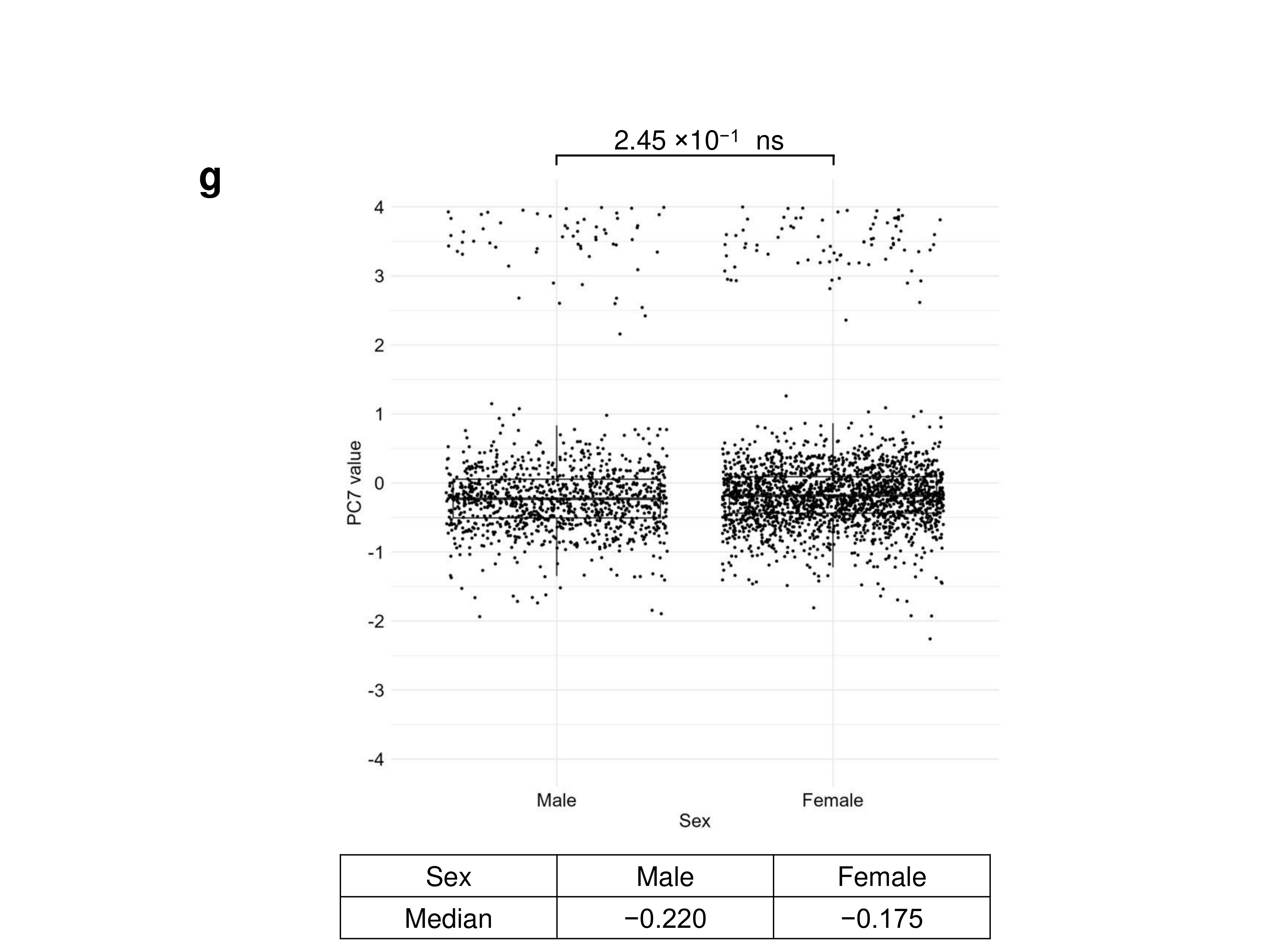

Supplement: Supplementary file 21 — Additional file 21. PC7 values stratified by sex. Two-tailed t-test p-values are computed for each plot. The median PC values for both sexes are displayed in each plot. p-values reported are two-tailed t-test p-values, with * indicating p < 0.05, ** p < 0.01, and *** p < 0.001. p > was considered statistically non-significant (ns). [file 40101_2024_383_MOESM21_ESM.png]

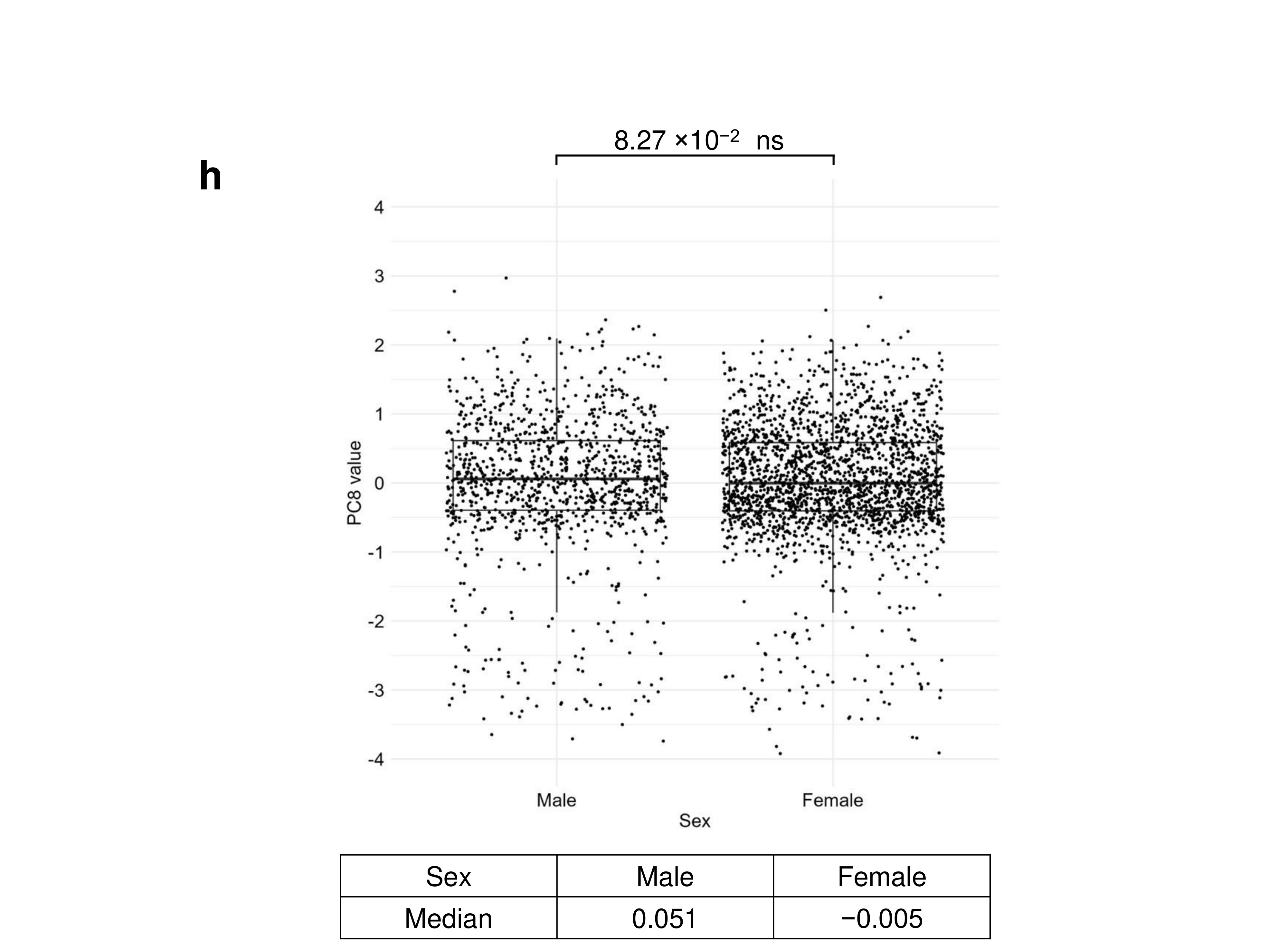

Supplement: Supplementary file 22 — Additional file 22. PC8 values stratified by sex. Two-tailed t-test p-values are computed for each plot. The median PC values for both sexes are displayed in each plot. p-values reported are two-tailed t-test p-values, with * indicating p < 0.05, ** p < 0.01, and *** p < 0.001. p > was considered statistically non-significant (ns). [file 40101_2024_383_MOESM22_ESM.png]

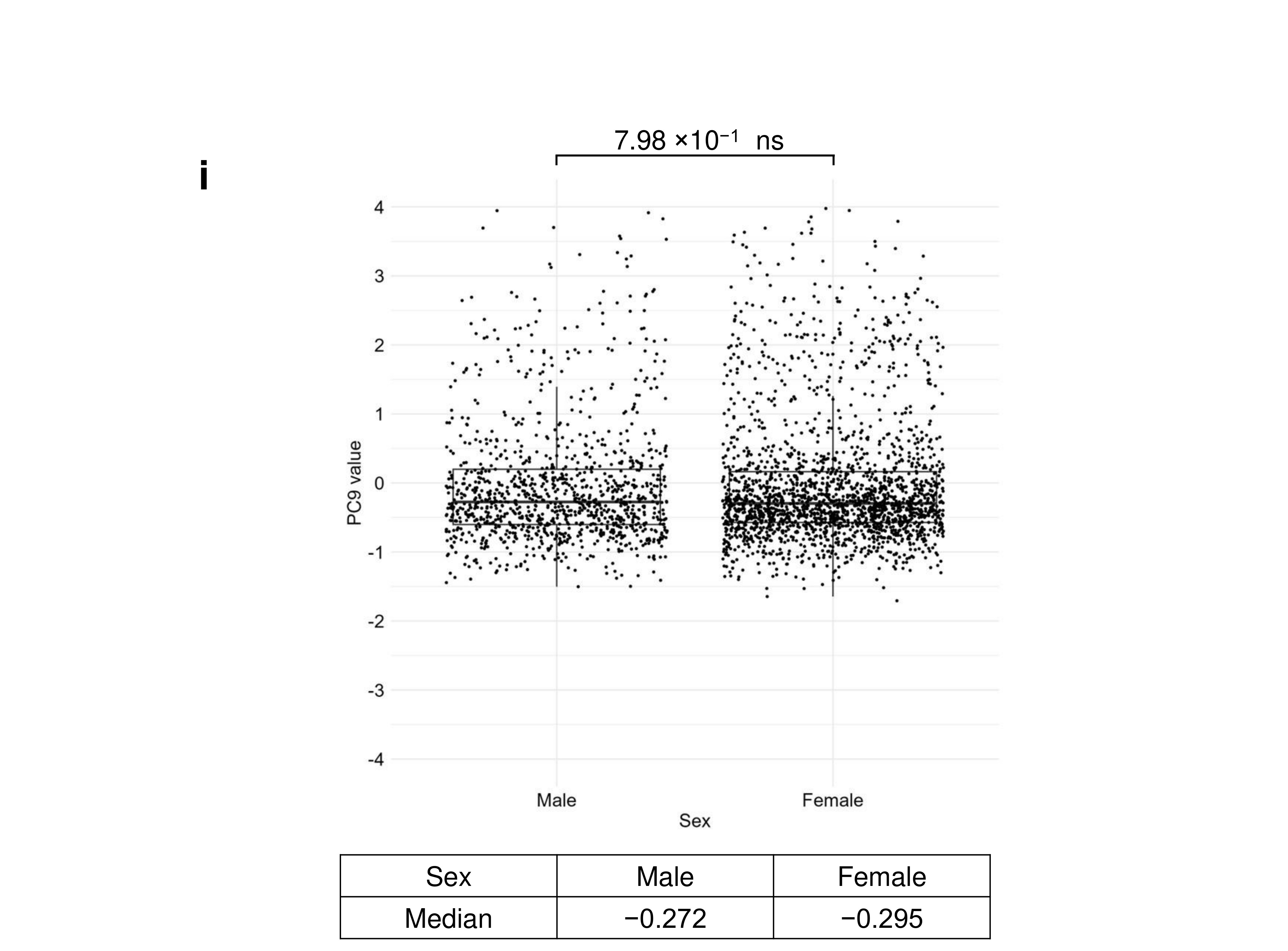

Supplement: Supplementary file 23 — Additional file 23. PC9 values stratified by sex. Two-tailed t-test p-values are computed for each plot. The median PC values for both sexes are displayed in each plot. p-values reported are two-tailed t-test p-values, with * indicating p < 0.05, ** p < 0.01, and *** p < 0.001. p > was considered statistically non-significant (ns). [file 40101_2024_383_MOESM23_ESM.png]

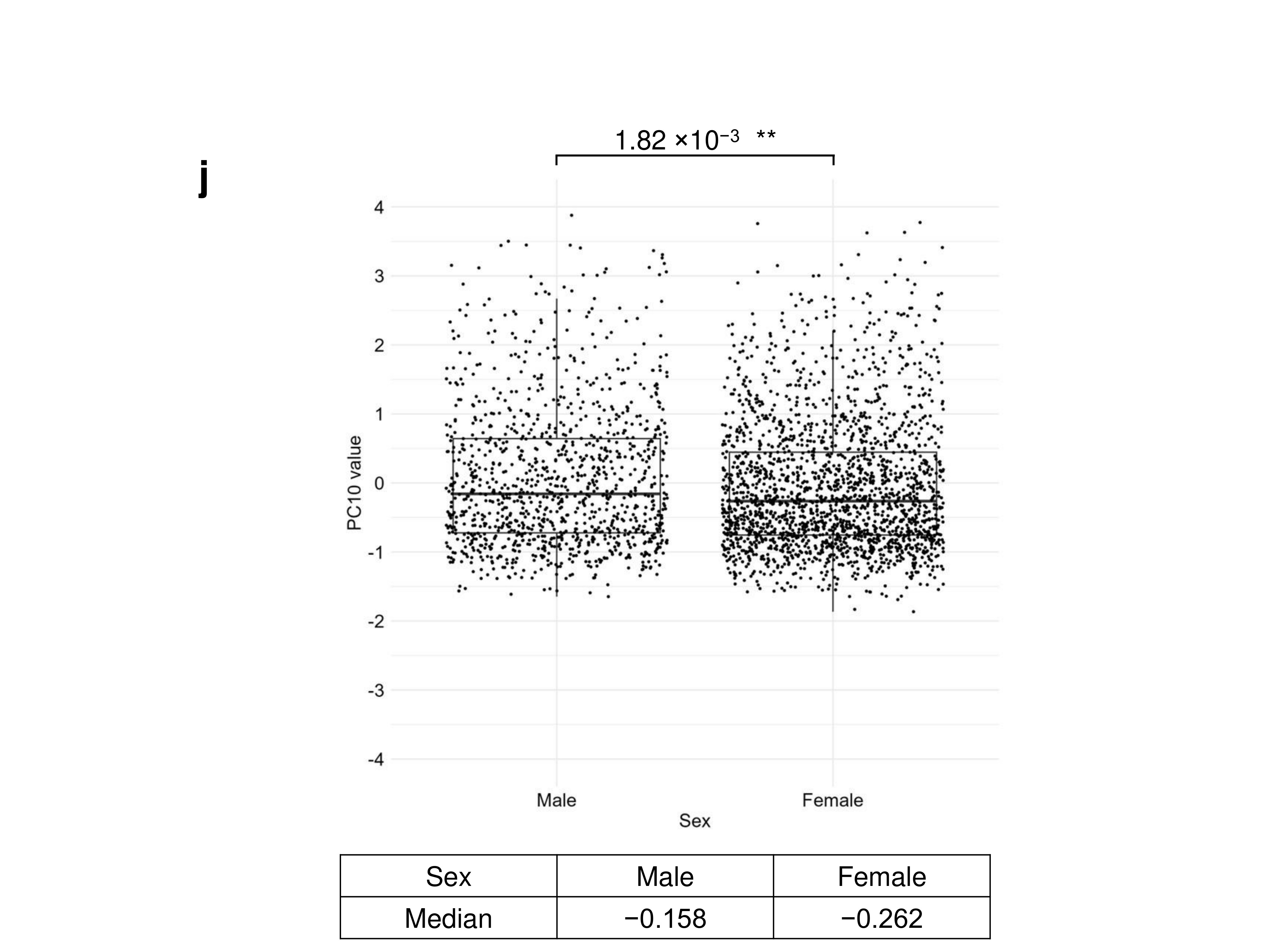

Supplement: Supplementary file 24 — Additional file 24. PC10 values stratified by sex. Two-tailed t-test p-values are computed for each plot. The median PC values for both sexes are displayed in each plot. p-values reported are two-tailed t-test p-values, with * indicating p < 0.05, ** p < 0.01, and *** p < 0.001. p > was considered statistically non-significant (ns). [file 40101_2024_383_MOESM24_ESM.png]

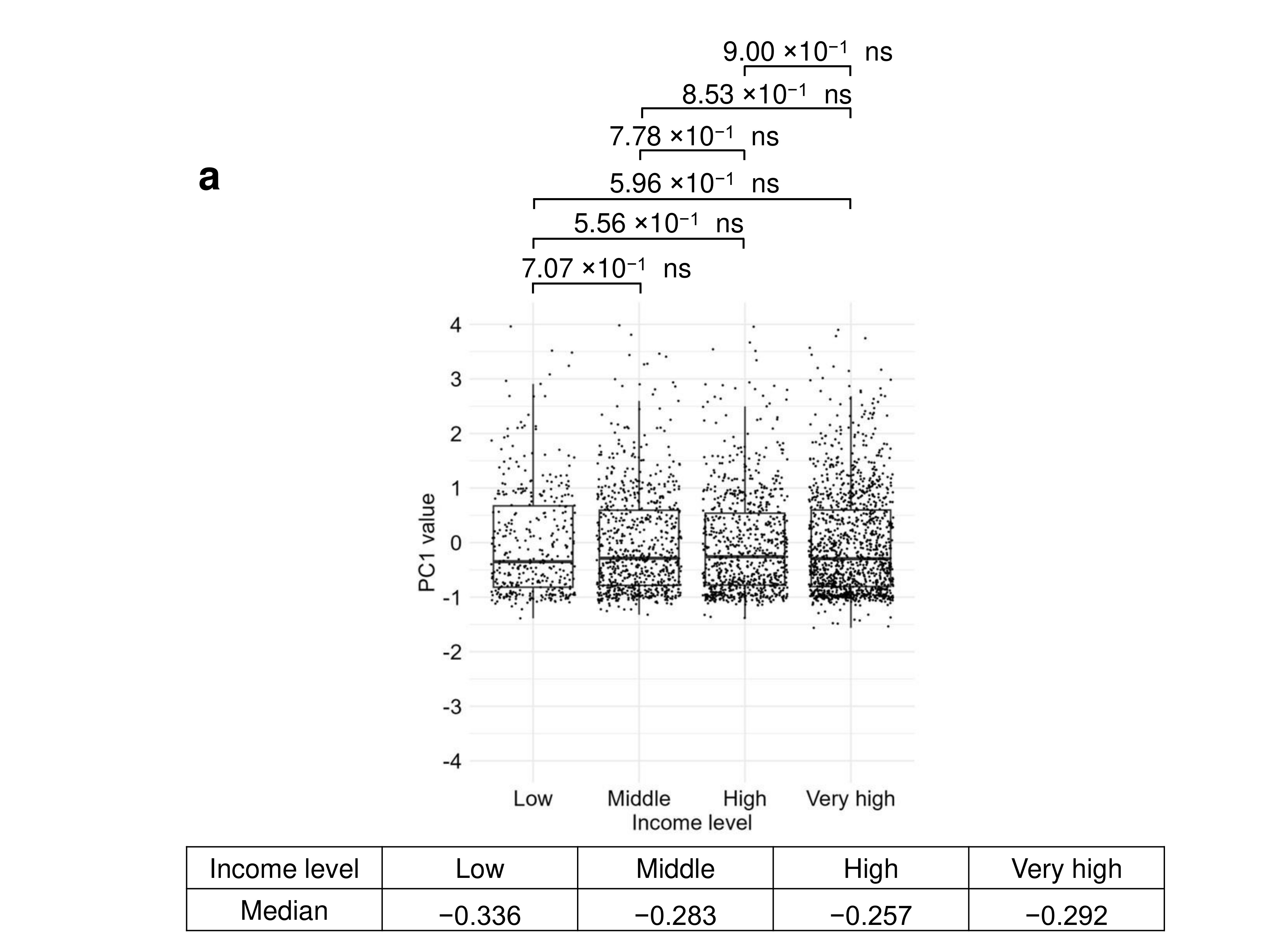

Supplement: Supplementary file 25 — Additional file 25. PC1 values grouped by income level. Two-tailed t-test p-values are provided for each plot. Income levels are based on self-reported total monthly family income per capita. In Singapore, income levels are classified as follows: low (< SGD 2000), moderate (SGD 2000–3999), high (SGD 4000–5999), and very high (> SGD 6000). In Malaysia, the classifications are low (< RM 3000), middle (RM 3000–5999), high (RM 6000–12,999), and very high (> RM 13,000). Each plot displays the median PC values for low, middle, high, and very high income groups.p-values reported are two-tailed t-test p-values, with * indicating p < 0.05, ** p < 0.01, and *** p < 0.001. p > 0.05 was considered statistically non-significant (ns). [file 40101_2024_383_MOESM25_ESM.png]

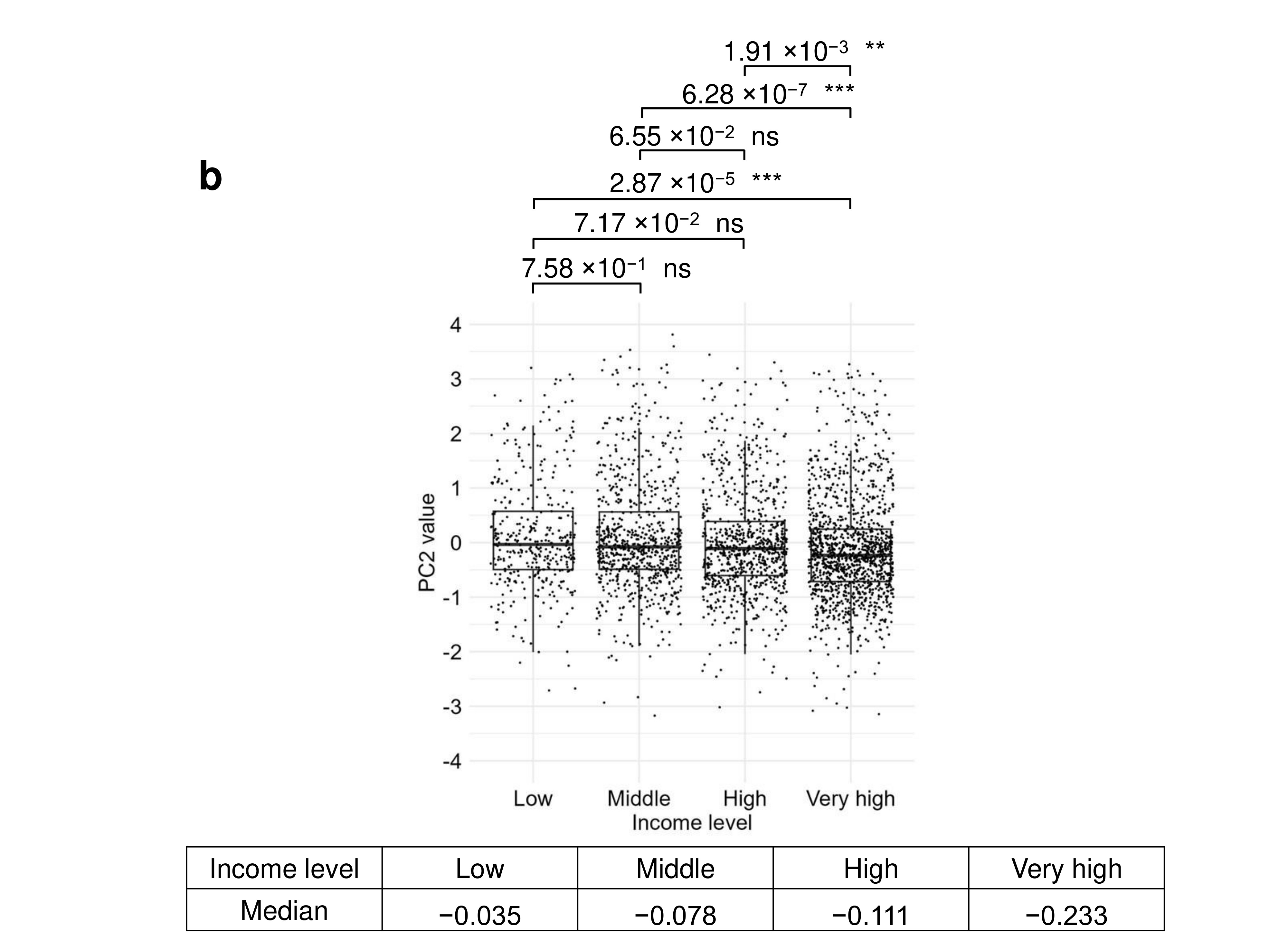

Supplement: Supplementary file 26 — Additional file 26. PC2 values grouped by income level. Two-tailed t-test p-values are provided for each plot. Income levels are based on self-reported total monthly family income per capita. In Singapore, income levels are classified as follows: low (< SGD 2000), moderate (SGD 2000–3999), high (SGD 4000–5999), and very high (> SGD 6000). In Malaysia, the classifications are low (< RM 3000), middle (RM 3000–5999), high (RM 6000–12,999), and very high (> RM 13,000). Each plot displays the median PC values for low, middle, high, and very high income groups.p-values reported are two-tailed t-test p-values, with * indicating p < 0.05, ** p < 0.01, and *** p < 0.001. p > 0.05 was considered statistically non-significant (ns). [file 40101_2024_383_MOESM26_ESM.png]

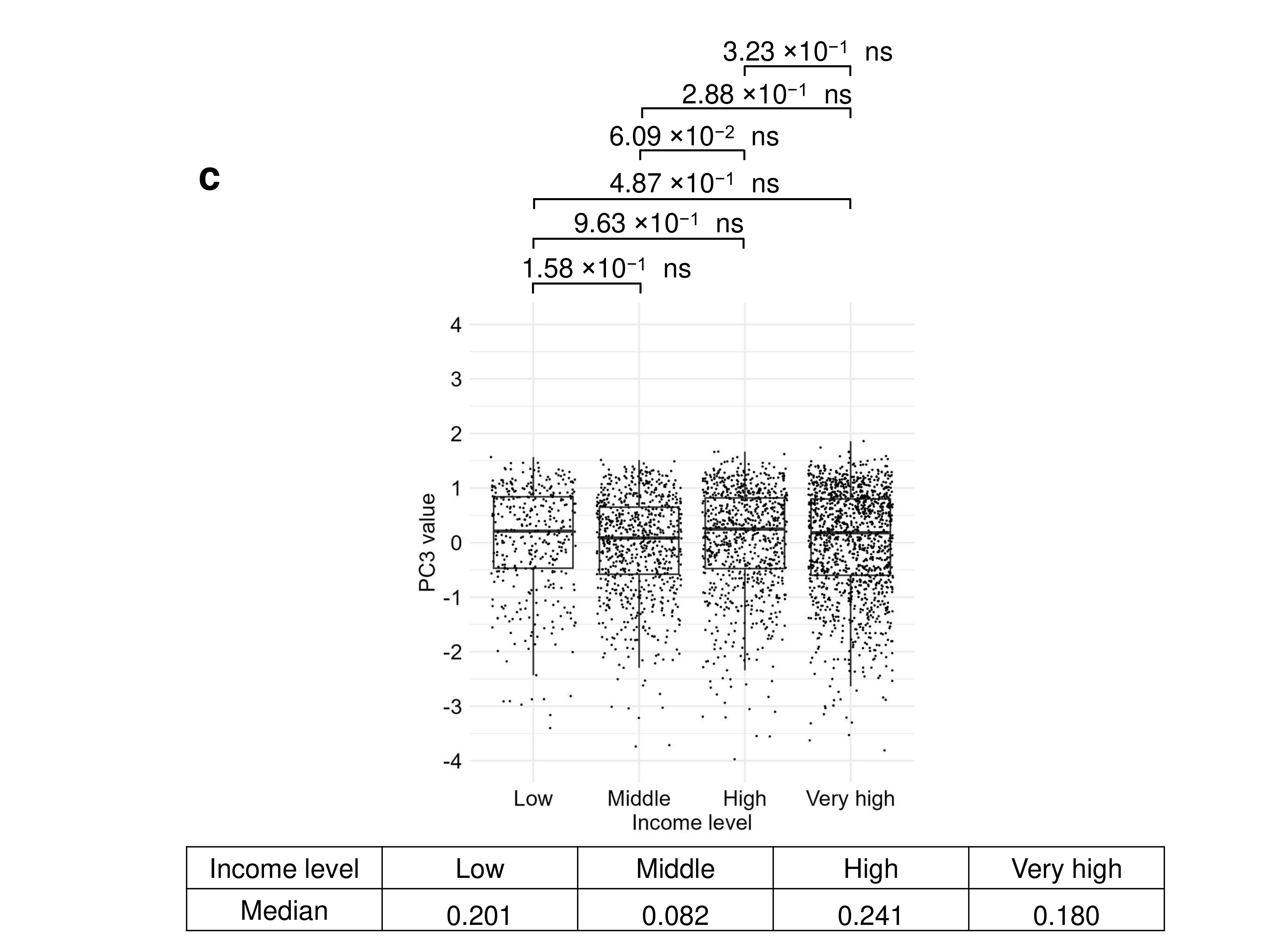

Supplement: Supplementary file 27 — Additional file 27. PC3 values grouped by income level. Two-tailed t-test p-values are provided for each plot. Income levels are based on self-reported total monthly family income per capita. In Singapore, income levels are classified as follows: low (< SGD 2000), moderate (SGD 2000–3999), high (SGD 4000–5999), and very high (> SGD 6000). In Malaysia, the classifications are low (< RM 3000), middle (RM 3000–5999), high (RM 6000–12,999), and very high (> RM 13,000). Each plot displays the median PC values for low, middle, high, and very high income groups.p-values reported are two-tailed t-test p-values, with * indicating p < 0.05, ** p < 0.01, and *** p < 0.001. p > 0.05 was considered statistically non-significant (ns). [file 40101_2024_383_MOESM27_ESM.png]

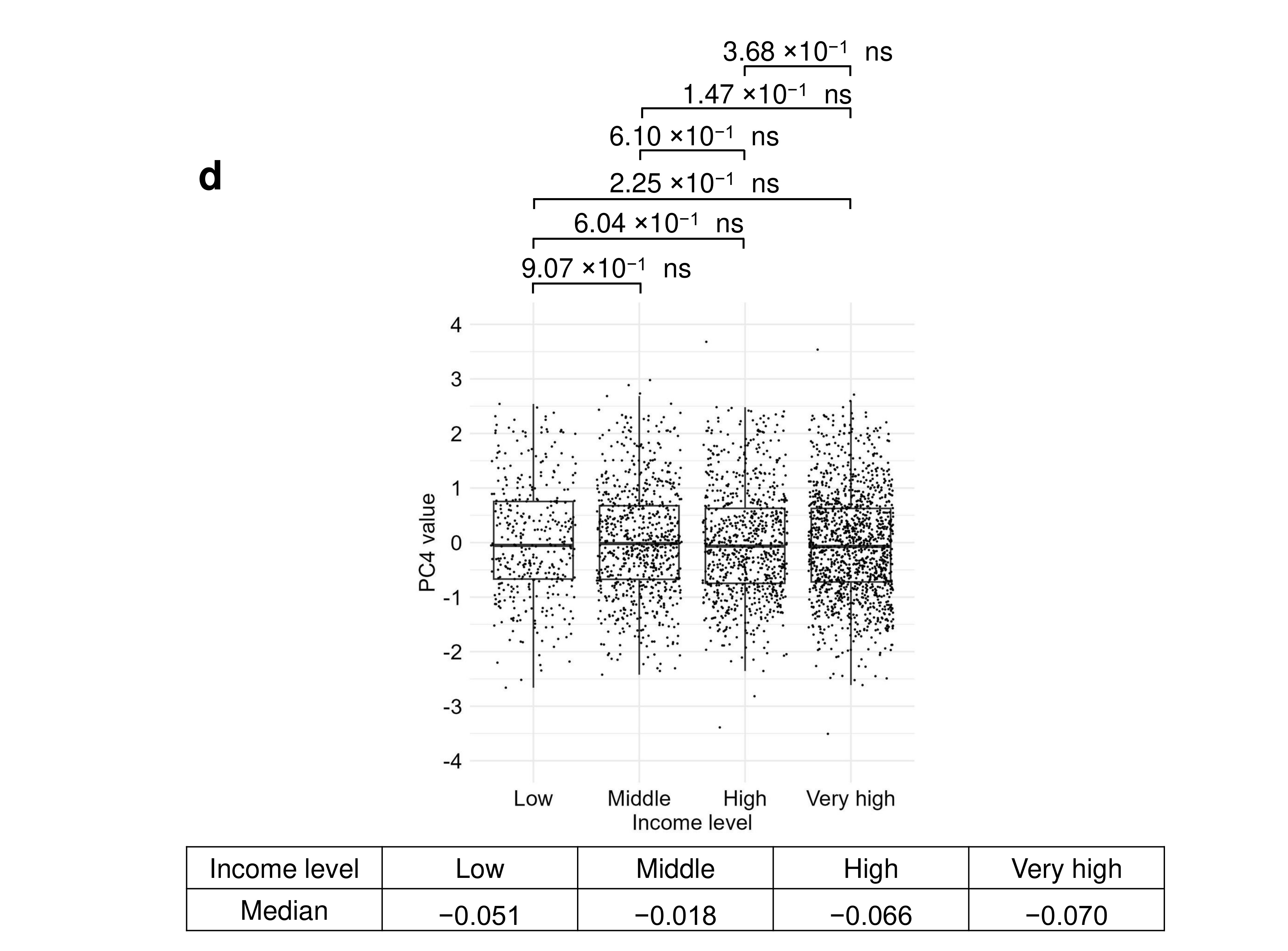

Supplement: Supplementary file 28 — Additional file 28. PC4 values grouped by income level. Two-tailed t-test p-values are provided for each plot. Income levels are based on self-reported total monthly family income per capita. In Singapore, income levels are classified as follows: low (< SGD 2000), moderate (SGD 2000–3999), high (SGD 4000–5999), and very high (> SGD 6000). In Malaysia, the classifications are low (< RM 3000), middle (RM 3000–5999), high (RM 6000–12,999), and very high (> RM 13,000). Each plot displays the median PC values for low, middle, high, and very high income groups.p-values reported are two-tailed t-test p-values, with * indicating p < 0.05, ** p < 0.01, and *** p < 0.001. p > 0.05 was considered statistically non-significant (ns). [file 40101_2024_383_MOESM28_ESM.png]

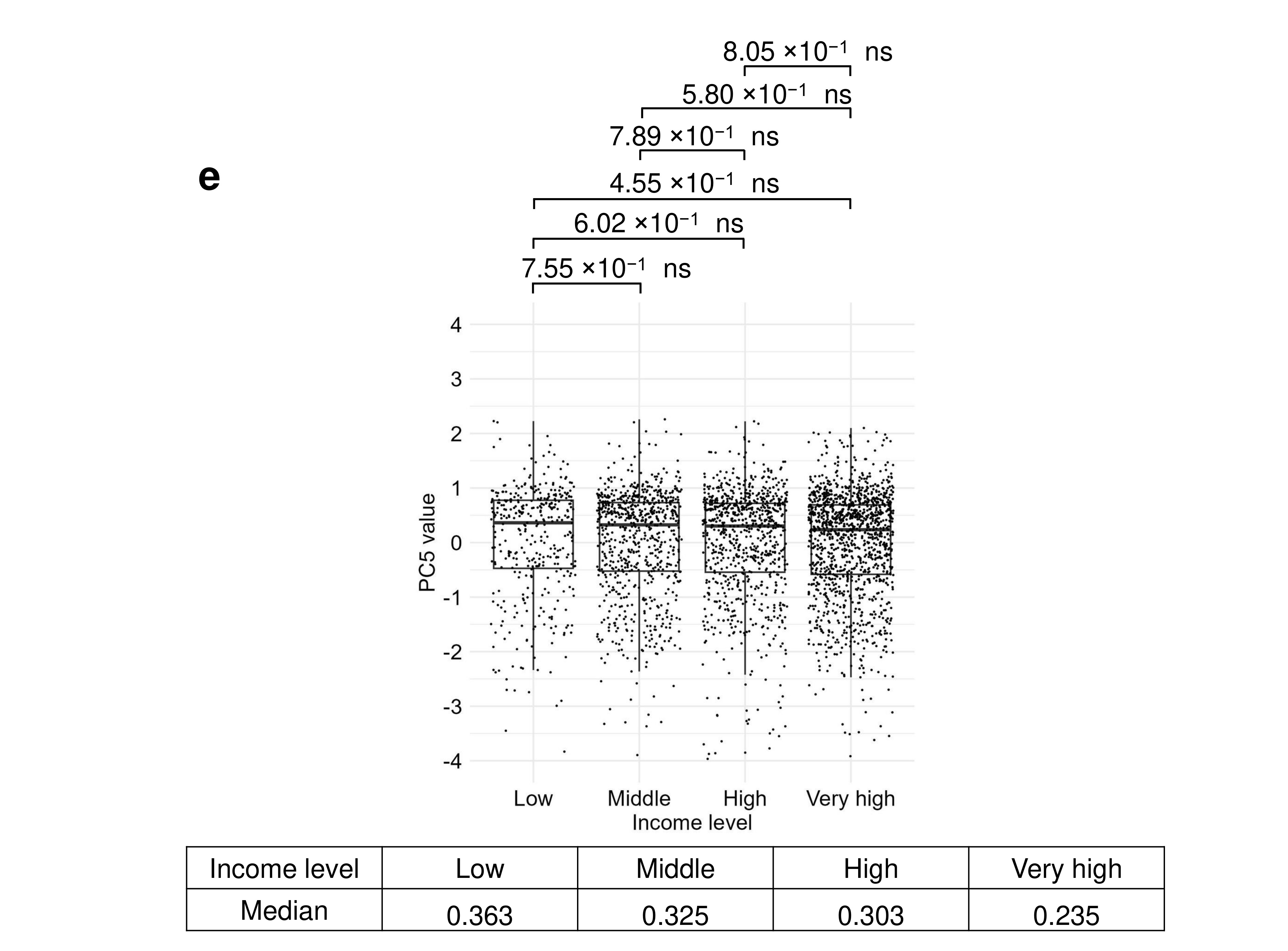

Supplement: Supplementary file 29 — Additional file 29. PC5 values grouped by income level. Two-tailed t-test p-values are provided for each plot. Income levels are based on self-reported total monthly family income per capita. In Singapore, income levels are classified as follows: low (< SGD 2000), moderate (SGD 2000–3999), high (SGD 4000–5999), and very high (> SGD 6000). In Malaysia, the classifications are low (< RM 3000), middle (RM 3000–5999), high (RM 6000–12,999), and very high (> RM 13,000). Each plot displays the median PC values for low, middle, high, and very high income groups.p-values reported are two-tailed t-test p-values, with * indicating p < 0.05, ** p < 0.01, and *** p < 0.001. p > 0.05 was considered statistically non-significant (ns). [file 40101_2024_383_MOESM29_ESM.png]

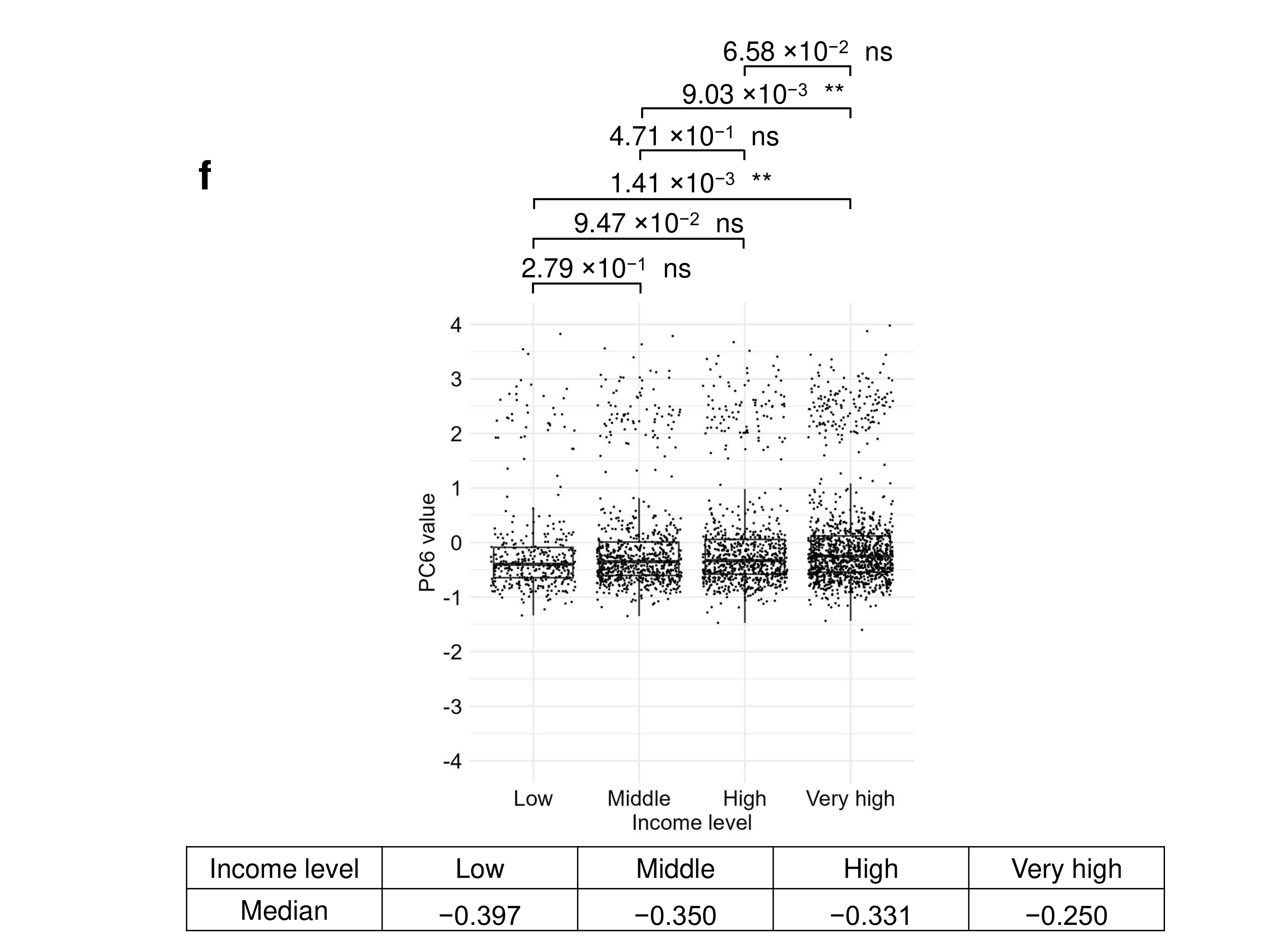

Supplement: Supplementary file 30 — Additional file 30. PC6 values grouped by income level. Two-tailed t-test p-values are provided for each plot. Income levels are based on self-reported total monthly family income per capita. In Singapore, income levels are classified as follows: low (< SGD 2000), moderate (SGD 2000–3999), high (SGD 4000–5999), and very high (> SGD 6000). In Malaysia, the classifications are low (< RM 3000), middle (RM 3000–5999), high (RM 6000–12,999), and very high (> RM 13,000). Each plot displays the median PC values for low, middle, high, and very high income groups.p-values reported are two-tailed t-test p-values, with * indicating p < 0.05, ** p < 0.01, and *** p < 0.001. p > 0.05 was considered statistically non-significant (ns). [file 40101_2024_383_MOESM30_ESM.png]

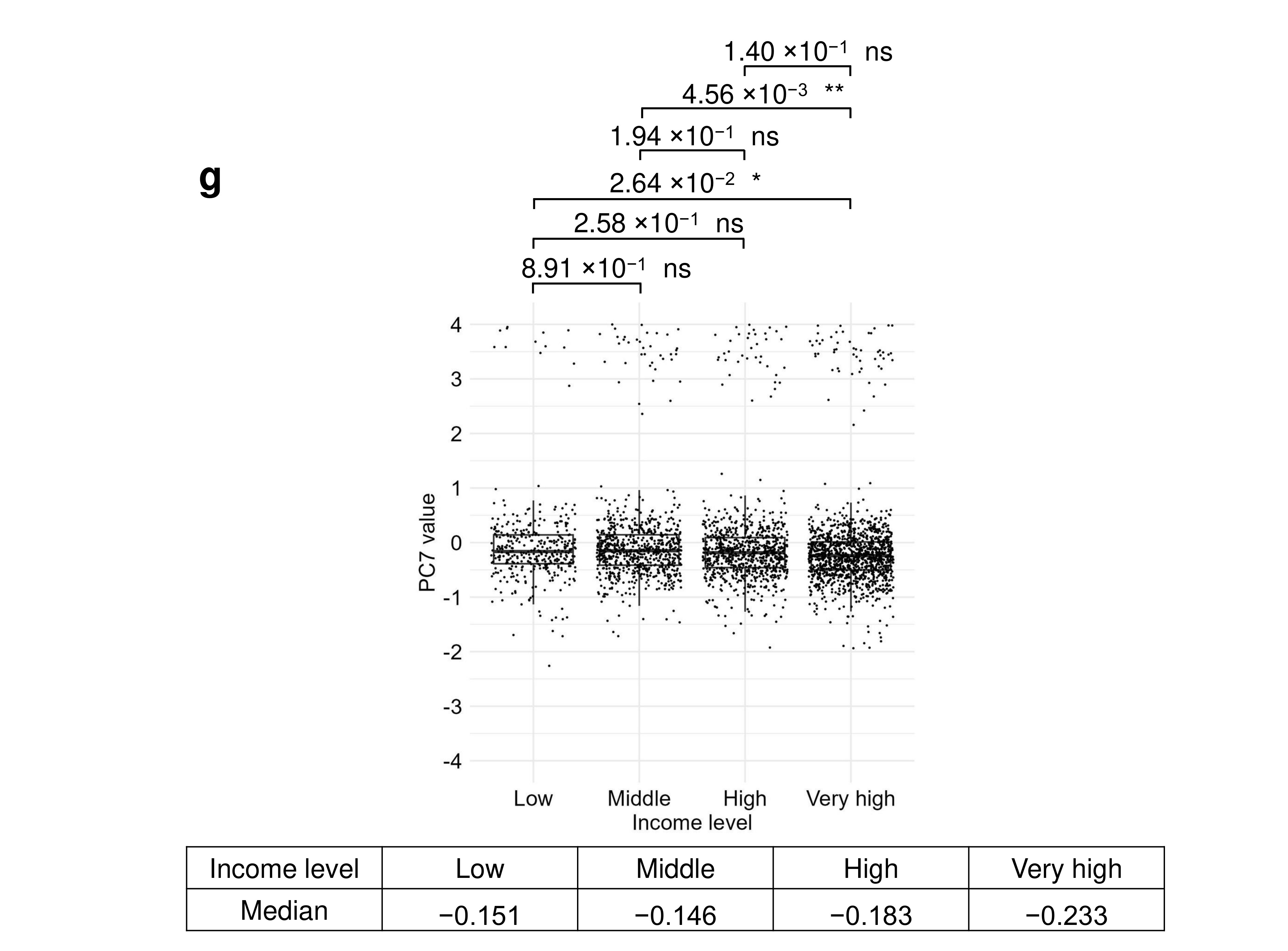

Supplement: Supplementary file 31 — Additional file 31. PC7 values grouped by income level. Two-tailed t-test p-values are provided for each plot. Income levels are based on self-reported total monthly family income per capita. In Singapore, income levels are classified as follows: low (< SGD 2000), moderate (SGD 2000–3999), high (SGD 4000–5999), and very high (> SGD 6000). In Malaysia, the classifications are low (< RM 3000), middle (RM 3000–5999), high (RM 6000–12,999), and very high (> RM 13,000). Each plot displays the median PC values for low, middle, high, and very high income groups.p-values reported are two-tailed t-test p-values, with * indicating p < 0.05, ** p < 0.01, and *** p < 0.001. p > 0.05 was considered statistically non-significant (ns). [file 40101_2024_383_MOESM31_ESM.png]

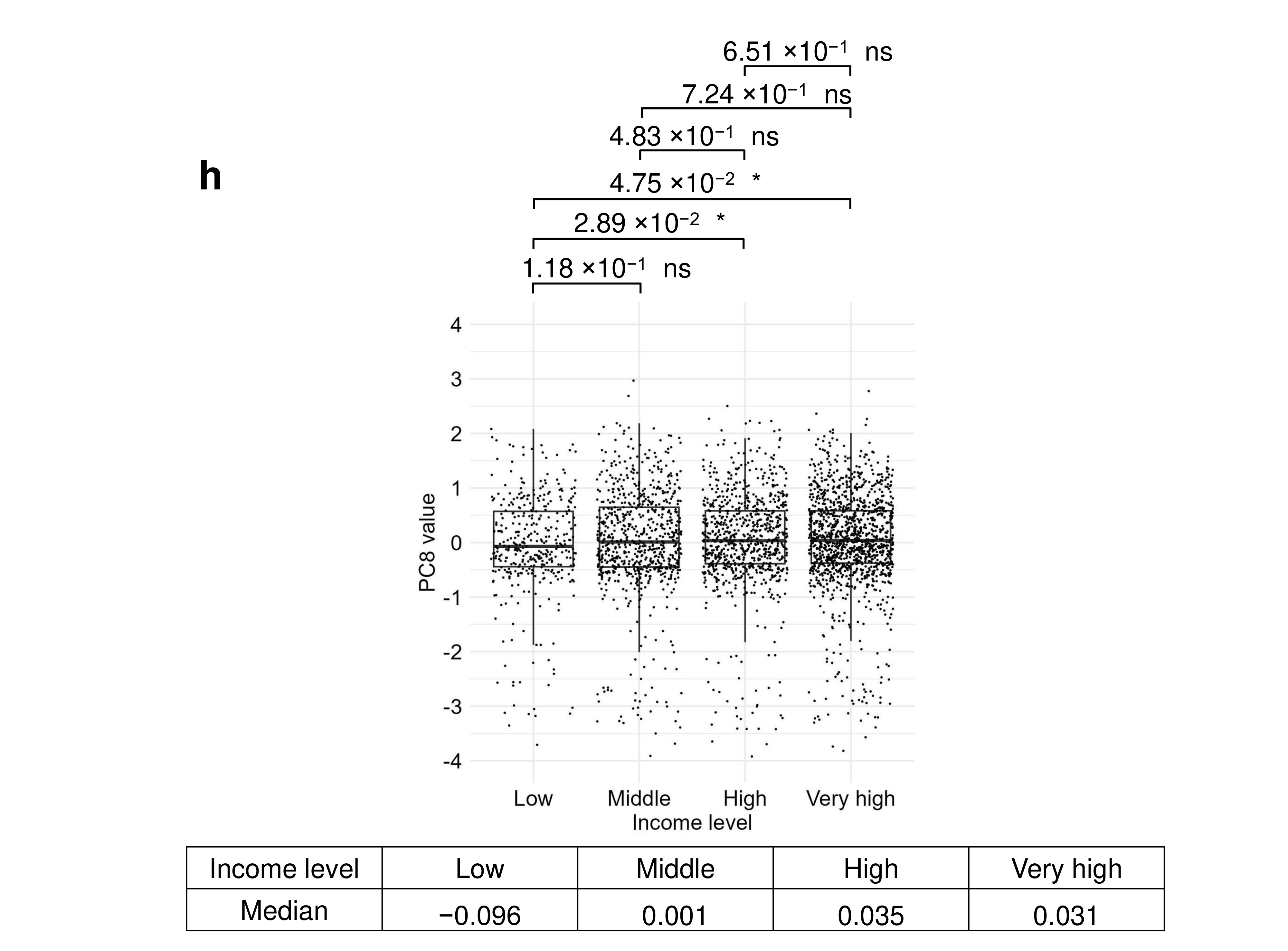

Supplement: Supplementary file 32 — Additional file 32. PC8 values grouped by income level. Two-tailed t-test p-values are provided for each plot. Income levels are based on self-reported total monthly family income per capita. In Singapore, income levels are classified as follows: low (< SGD 2000), moderate (SGD 2000–3999), high (SGD 4000–5999), and very high (> SGD 6000). In Malaysia, the classifications are low (< RM 3000), middle (RM 3000–5999), high (RM 6000–12,999), and very high (> RM 13,000). Each plot displays the median PC values for low, middle, high, and very high income groups.p-values reported are two-tailed t-test p-values, with * indicating p < 0.05, ** p < 0.01, and *** p < 0.001. p > 0.05 was considered statistically non-significant (ns). [file 40101_2024_383_MOESM32_ESM.png]

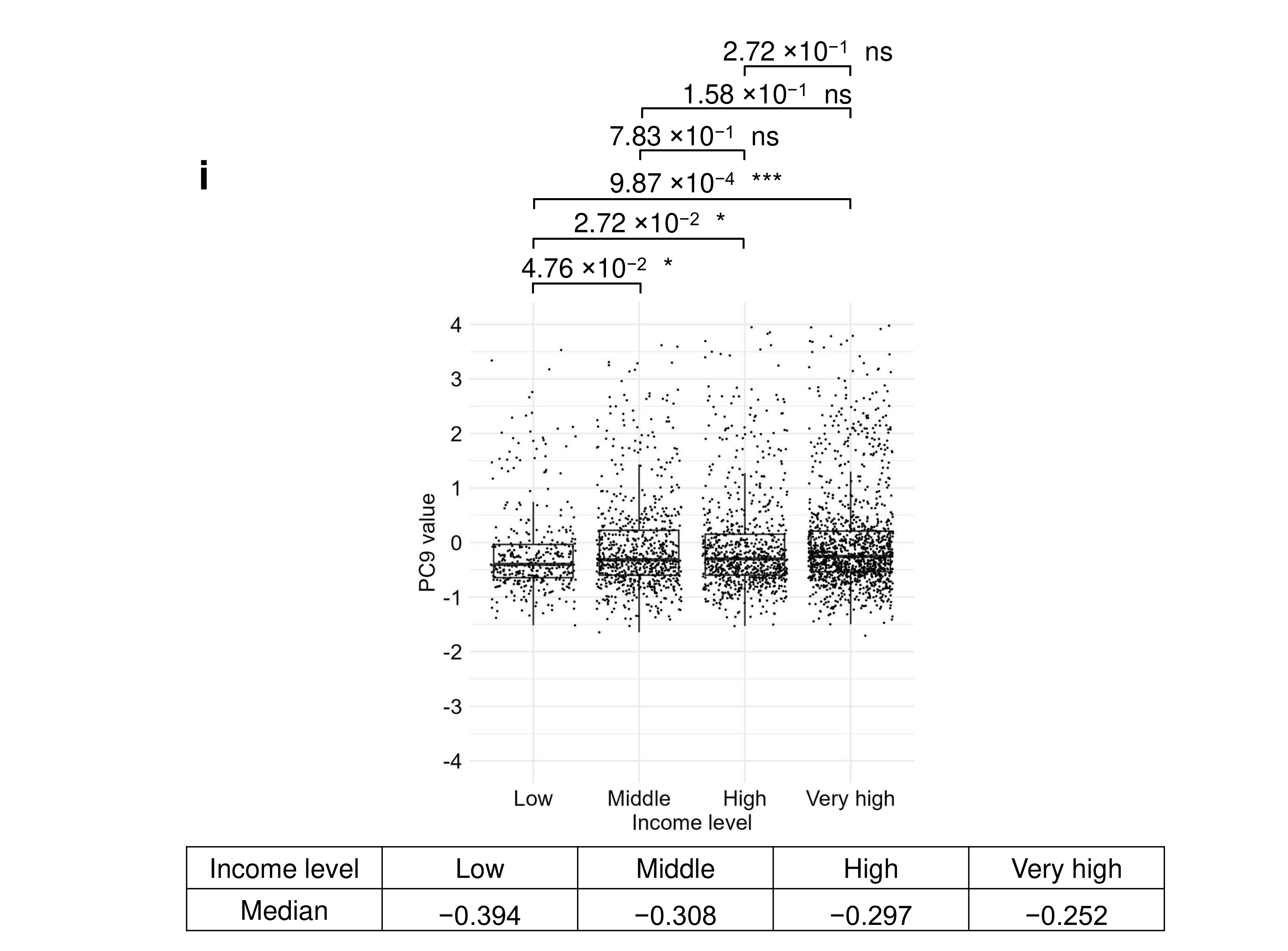

Supplement: Supplementary file 33 — Additional file 33. PC9 values grouped by income level. Two-tailed t-test p-values are provided for each plot. Income levels are based on self-reported total monthly family income per capita. In Singapore, income levels are classified as follows: low (< SGD 2000), moderate (SGD 2000–3999), high (SGD 4000–5999), and very high (> SGD 6000). In Malaysia, the classifications are low (< RM 3000), middle (RM 3000–5999), high (RM 6000–12,999), and very high (> RM 13,000). Each plot displays the median PC values for low, middle, high, and very high income groups.p-values reported are two-tailed t-test p-values, with * indicating p < 0.05, ** p < 0.01, and *** p < 0.001. p > 0.05 was considered statistically non-significant (ns). [file 40101_2024_383_MOESM33_ESM.png]

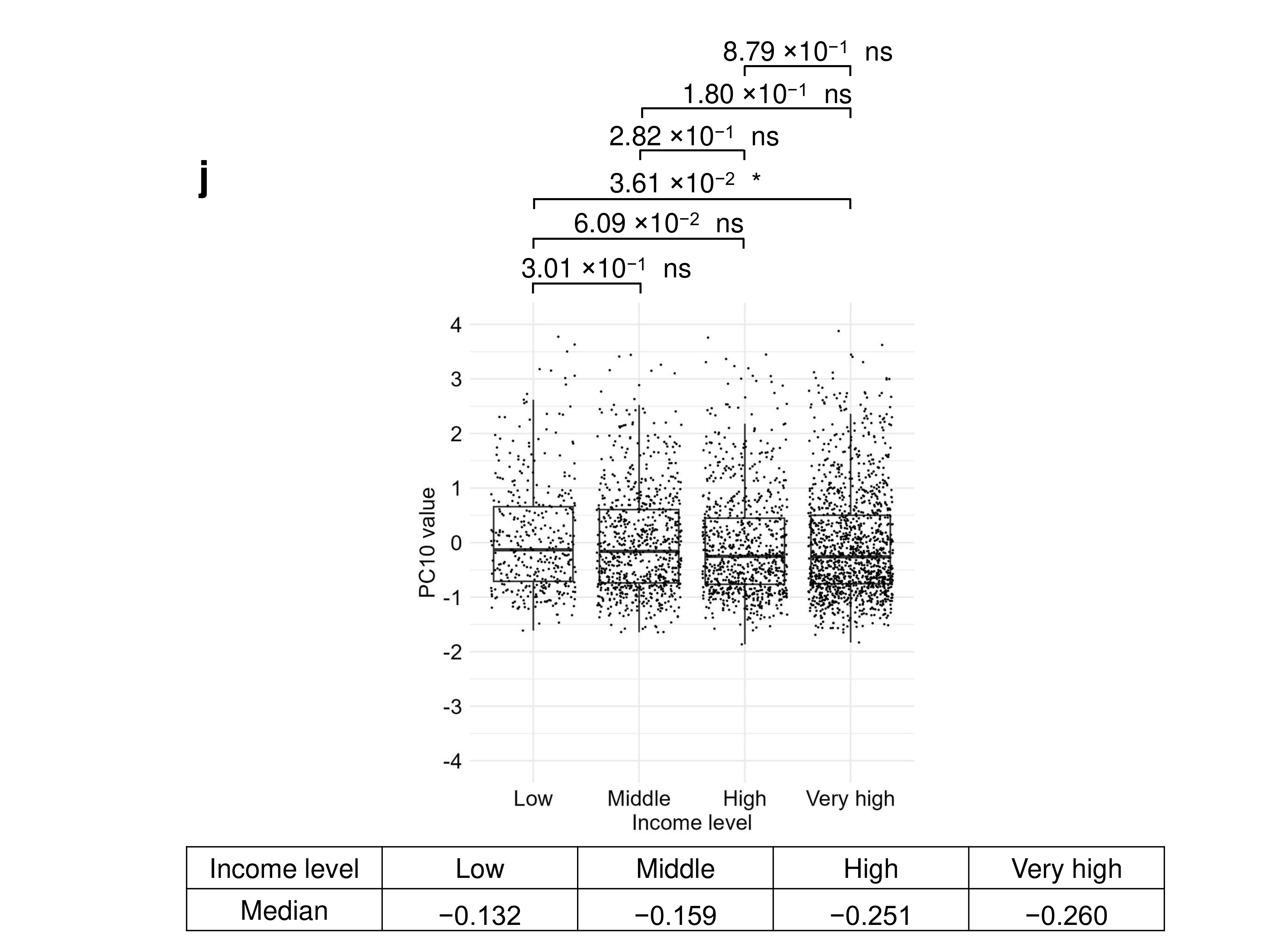

Supplement: Supplementary file 34 — Additional file 34. PC10 values grouped by income level. Two-tailed t-test p-values are provided for each plot. Income levels are based on self-reported total monthly family income per capita. In Singapore, income levels are classified as follows: low (< SGD 2000), moderate (SGD 2000–3999), high (SGD 4000–5999), and very high (> SGD 6000). In Malaysia, the classifications are low (< RM 3000), middle (RM 3000–5999), high (RM 6000–12,999), and very high (> RM 13,000). Each plot displays the median PC values for low, middle, high, and very high income groups.p-values reported are two-tailed t-test p-values, with * indicating p < 0.05, ** p < 0.01, and *** p < 0.001. p > 0.05 was considered statistically non-significant (ns). [file 40101_2024_383_MOESM34_ESM.png]

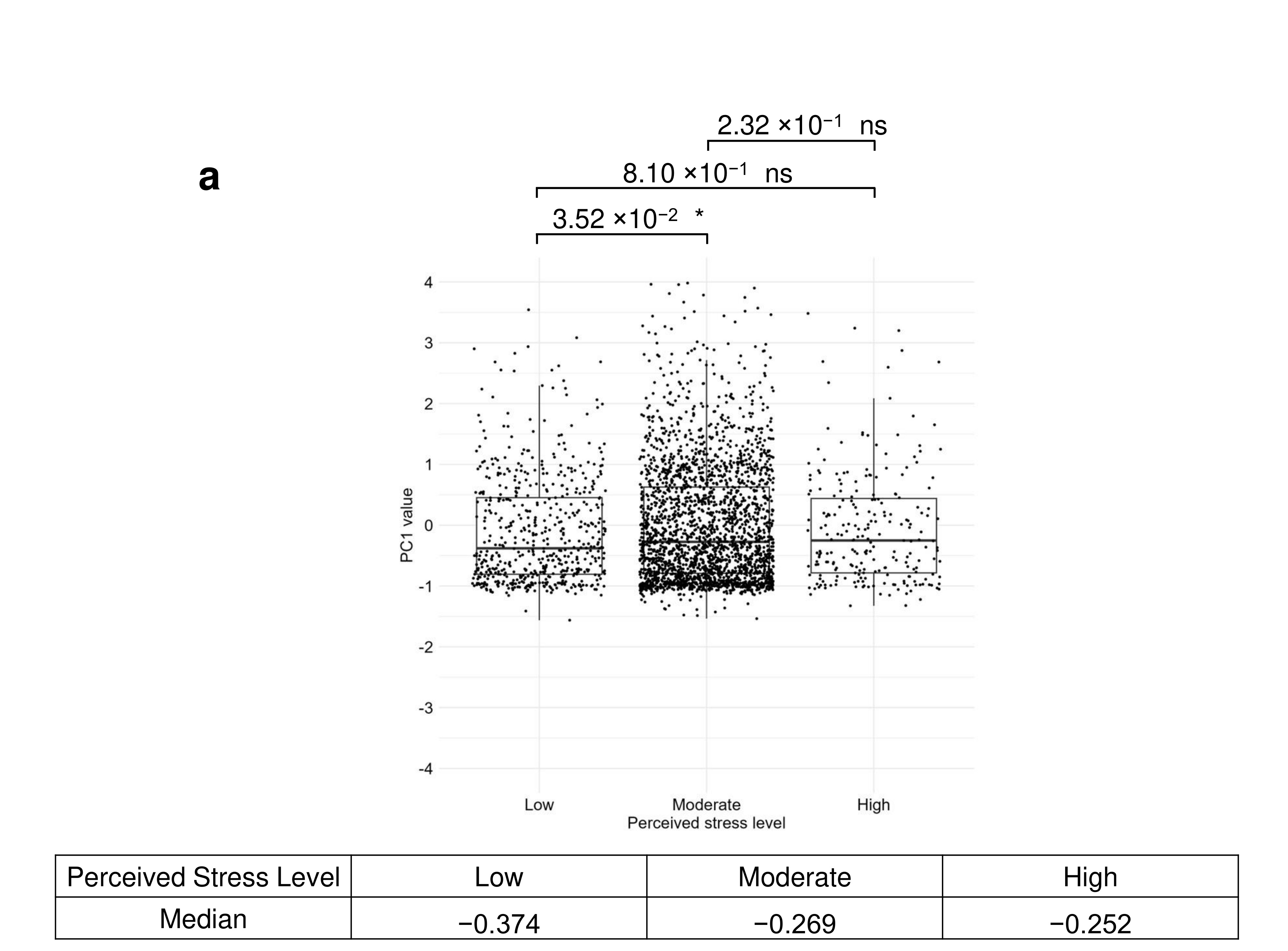

Supplement: Supplementary file 35 — Additional file 35. PC1 values stratified by perceived stress level. Two-tailed t-test p-values are computed for each plot. Perceived stress levels are based on self-reported scores from the Perceived Stress Scale (PSS): low stress (0–13), moderate stress (14–26), and high stress (27–40). The median PC values for low stress, moderate stress, and high stress levels are displayed in each plot. p-values reported are two-tailed t-test p-values, with * indicating p< 0.05, ** p < 0.01, and *** p < 0.001. p > 0.05 was considered statistically non-significant (ns). [file 40101_2024_383_MOESM35_ESM.png]

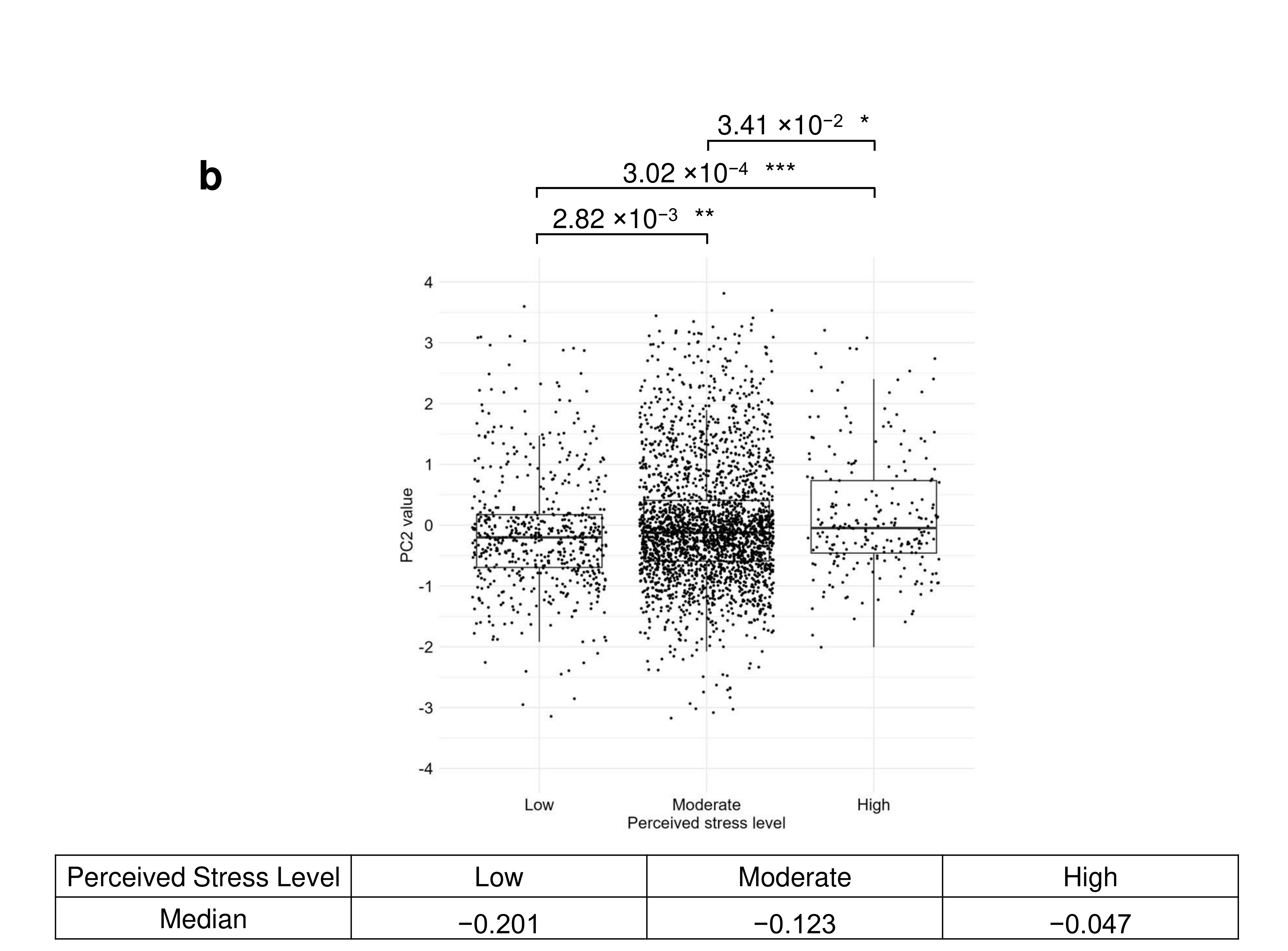

Supplement: Supplementary file 36 — Additional file 36. PC2 values stratified by perceived stress level. Two-tailed t-test p-values are computed for each plot. Perceived stress levels are based on self-reported scores from the Perceived Stress Scale (PSS): low stress (0–13), moderate stress (14–26), and high stress (27–40). The median PC values for low stress, moderate stress, and high stress levels are displayed in each plot. p-values reported are two-tailed t-test p-values, with * indicating p< 0.05, ** p < 0.01, and *** p < 0.001. p > 0.05 was considered statistically non-significant (ns). [file 40101_2024_383_MOESM36_ESM.png]

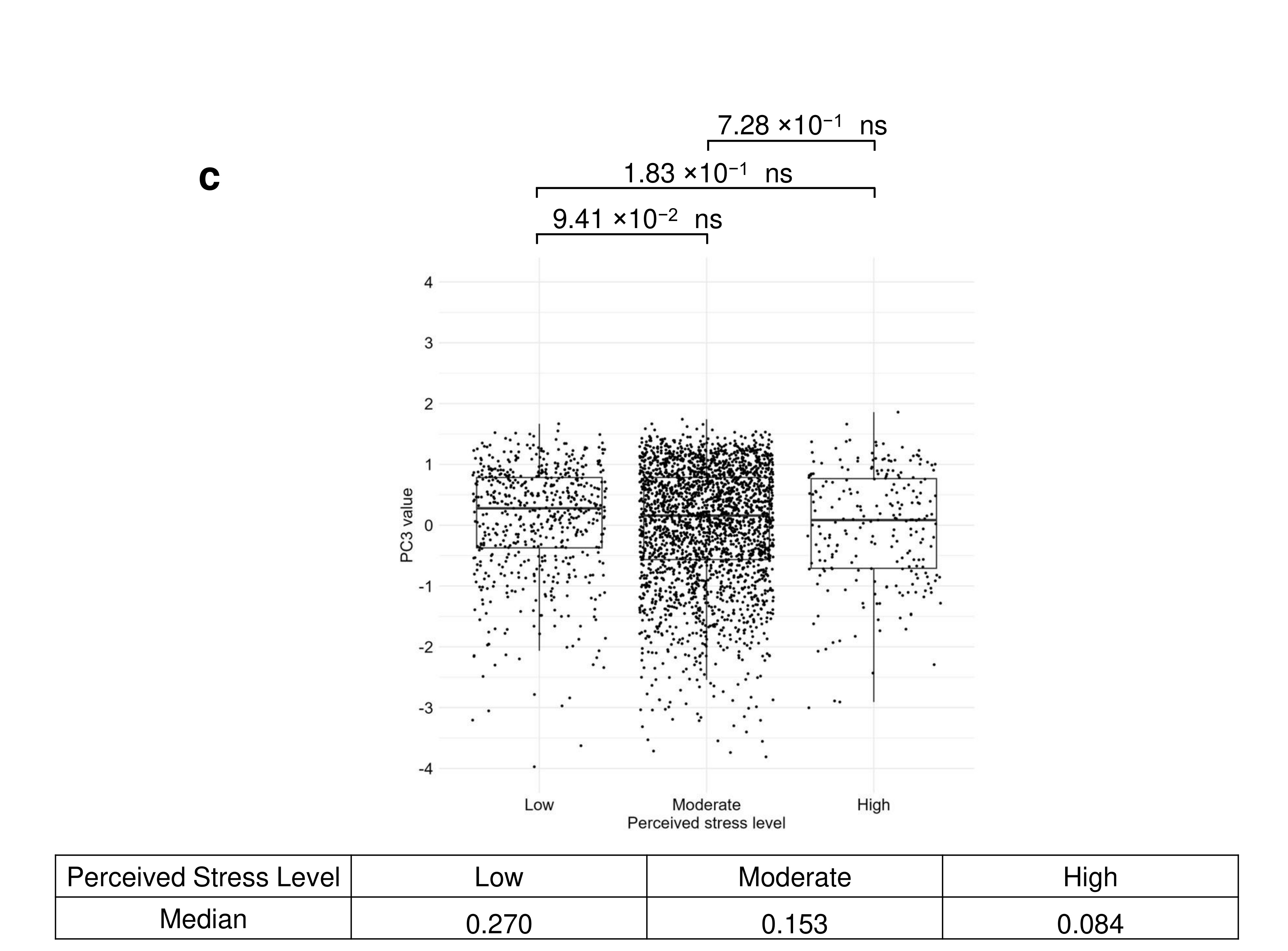

Supplement: Supplementary file 37 — Additional file 37. PC3 values stratified by perceived stress level. Two-tailed t-test p-values are computed for each plot. Perceived stress levels are based on self-reported scores from the Perceived Stress Scale (PSS): low stress (0–13), moderate stress (14–26), and high stress (27–40). The median PC values for low stress, moderate stress, and high stress levels are displayed in each plot. p-values reported are two-tailed t-test p-values, with * indicating p< 0.05, ** p < 0.01, and *** p < 0.001. p > 0.05 was considered statistically non-significant (ns). [file 40101_2024_383_MOESM37_ESM.png]

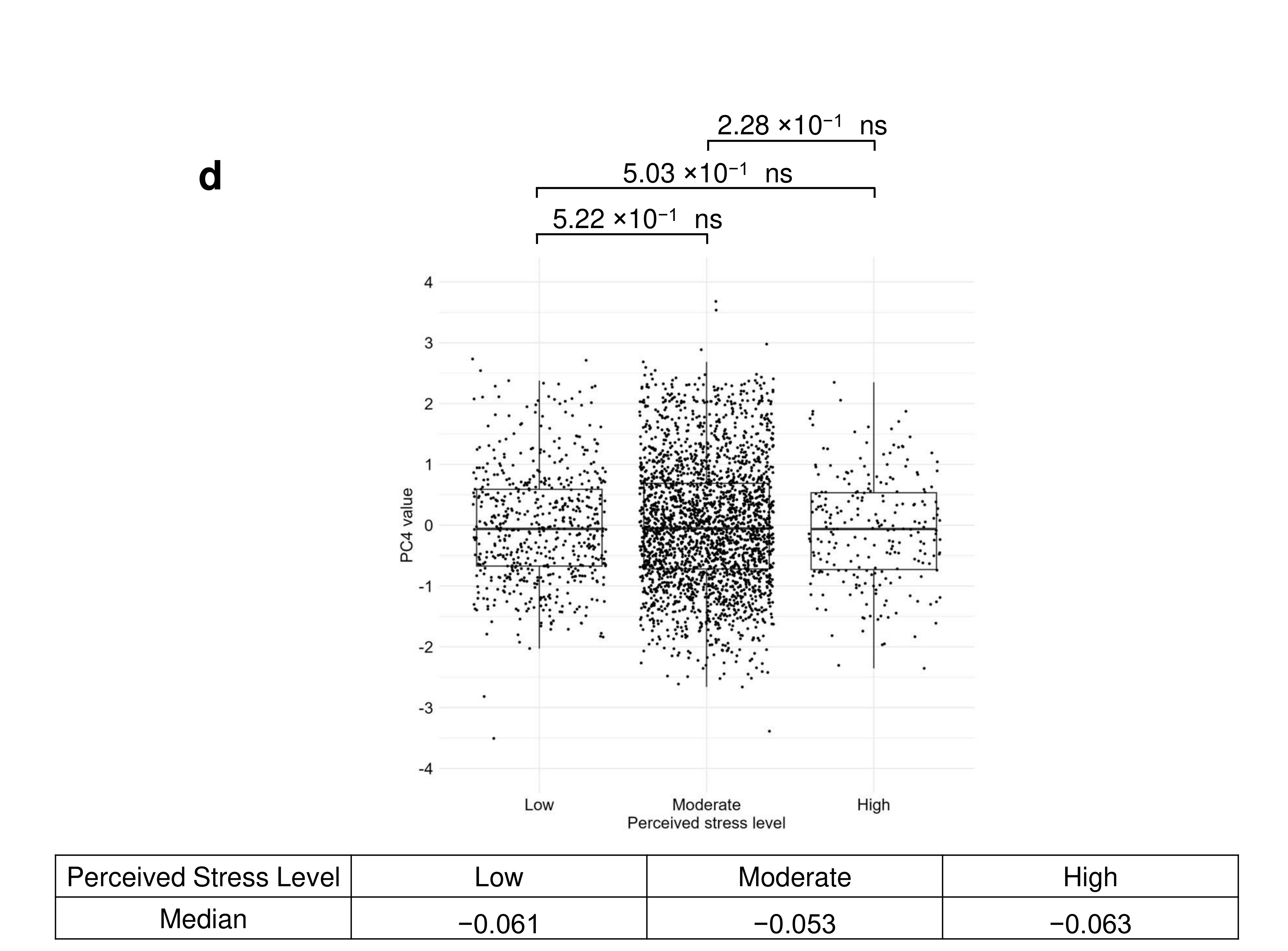

Supplement: Supplementary file 38 — Additional file 38. PC4 values stratified by perceived stress level. Two-tailed t-test p-values are computed for each plot. Perceived stress levels are based on self-reported scores from the Perceived Stress Scale (PSS): low stress (0–13), moderate stress (14–26), and high stress (27–40). The median PC values for low stress, moderate stress, and high stress levels are displayed in each plot. p-values reported are two-tailed t-test p-values, with * indicating p< 0.05, ** p < 0.01, and *** p < 0.001. p > 0.05 was considered statistically non-significant (ns). [file 40101_2024_383_MOESM38_ESM.png]

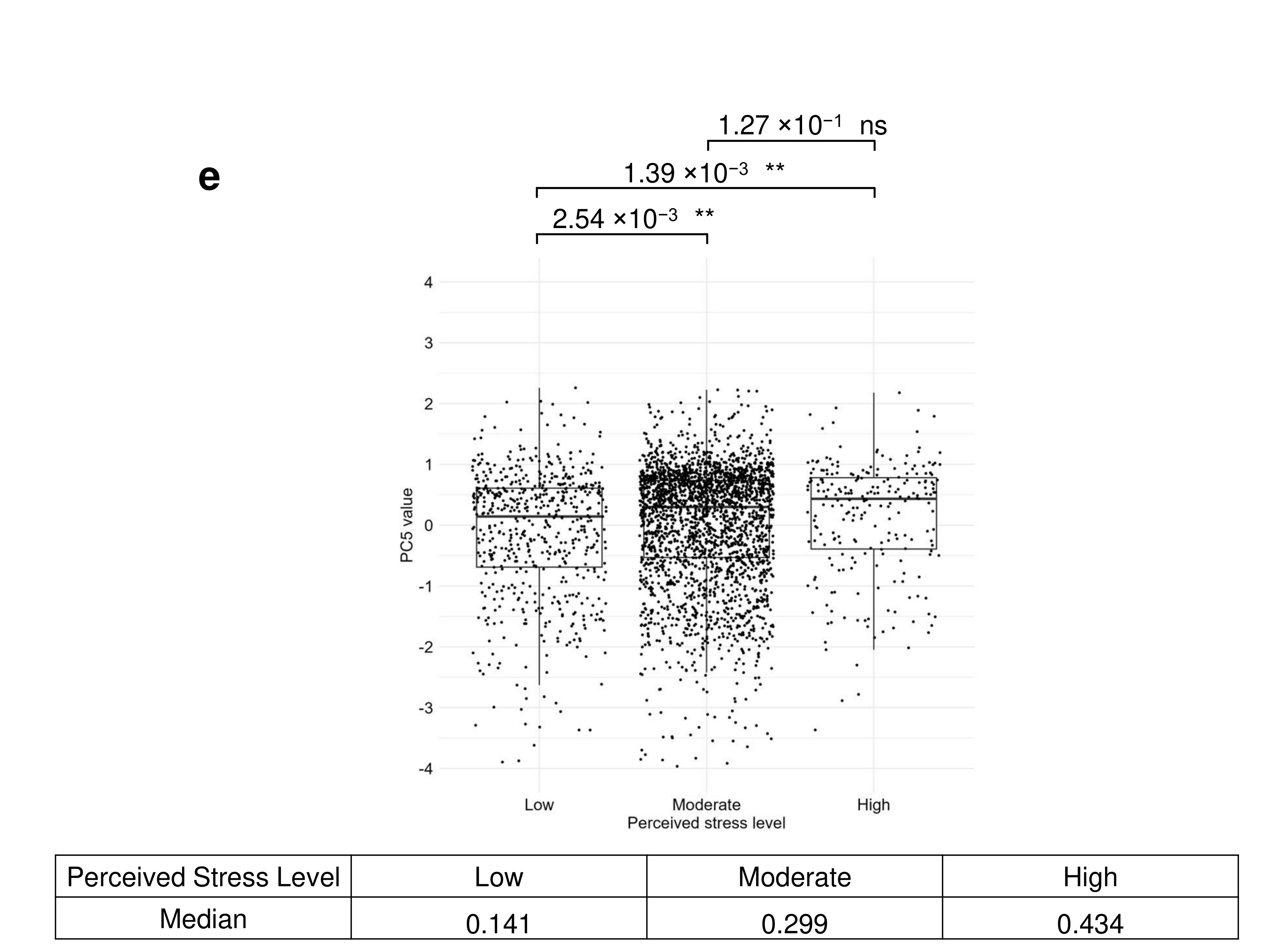

Supplement: Supplementary file 39 — Additional file 39. PC5 values stratified by perceived stress level. Two-tailed t-test p-values are computed for each plot. Perceived stress levels are based on self-reported scores from the Perceived Stress Scale (PSS): low stress (0–13), moderate stress (14–26), and high stress (27–40). The median PC values for low stress, moderate stress, and high stress levels are displayed in each plot. p-values reported are two-tailed t-test p-values, with * indicating p< 0.05, ** p < 0.01, and *** p < 0.001. p > 0.05 was considered statistically non-significant (ns). [file 40101_2024_383_MOESM39_ESM.png]

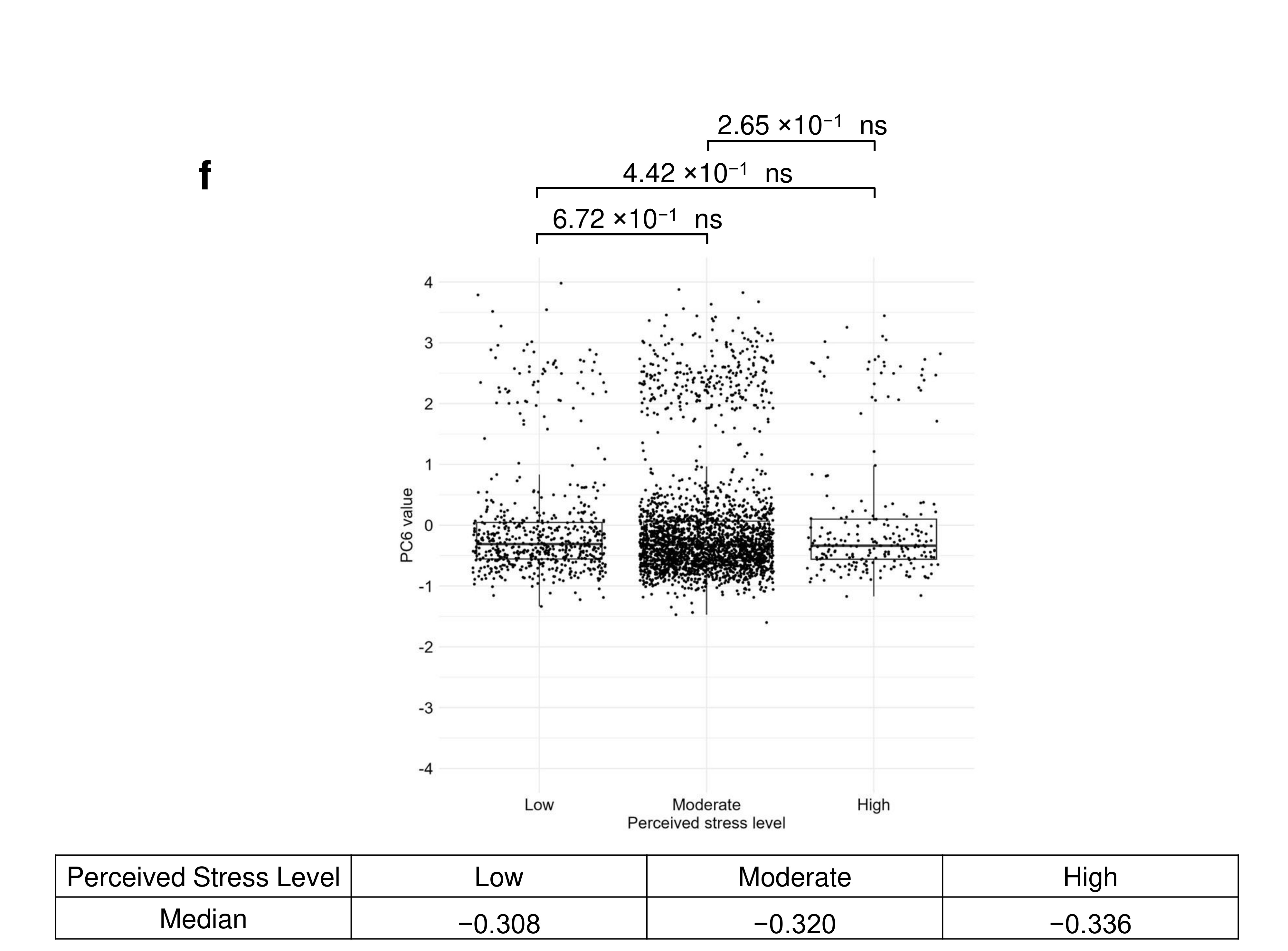

Supplement: Supplementary file 40 — Additional file 40. PC6 values stratified by perceived stress level. Two-tailed t-test p-values are computed for each plot. Perceived stress levels are based on self-reported scores from the Perceived Stress Scale (PSS): low stress (0–13), moderate stress (14–26), and high stress (27–40). The median PC values for low stress, moderate stress, and high stress levels are displayed in each plot. p-values reported are two-tailed t-test p-values, with * indicating p< 0.05, ** p < 0.01, and *** p < 0.001. p > 0.05 was considered statistically non-significant (ns). [file 40101_2024_383_MOESM40_ESM.png]

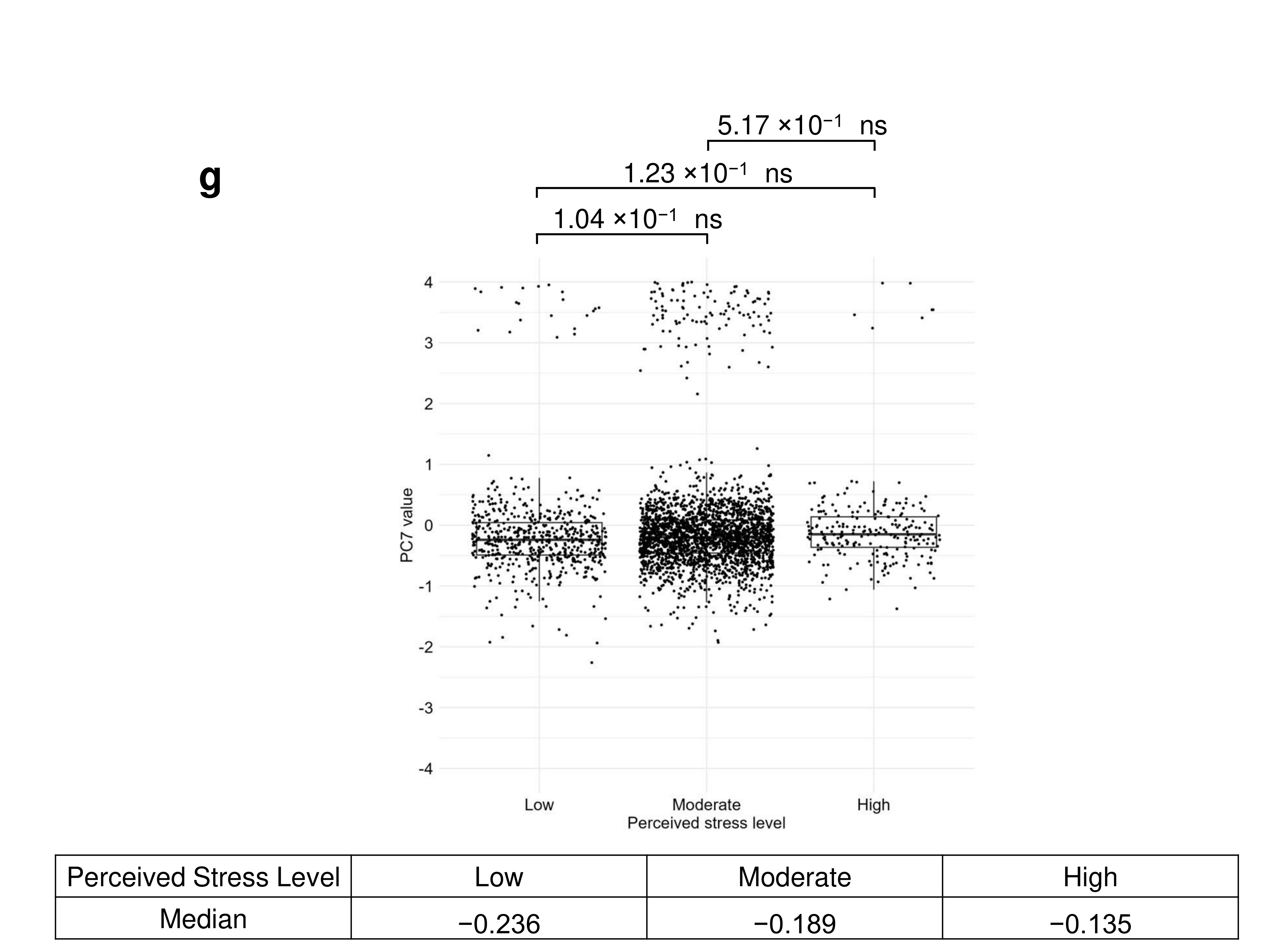

Supplement: Supplementary file 41 — Additional file 41. PC7 values stratified by perceived stress level. Two-tailed t-test p-values are computed for each plot. Perceived stress levels are based on self-reported scores from the Perceived Stress Scale (PSS): low stress (0–13), moderate stress (14–26), and high stress (27–40). The median PC values for low stress, moderate stress, and high stress levels are displayed in each plot. p-values reported are two-tailed t-test p-values, with * indicating p< 0.05, ** p < 0.01, and *** p < 0.001. p > 0.05 was considered statistically non-significant (ns). [file 40101_2024_383_MOESM41_ESM.png]

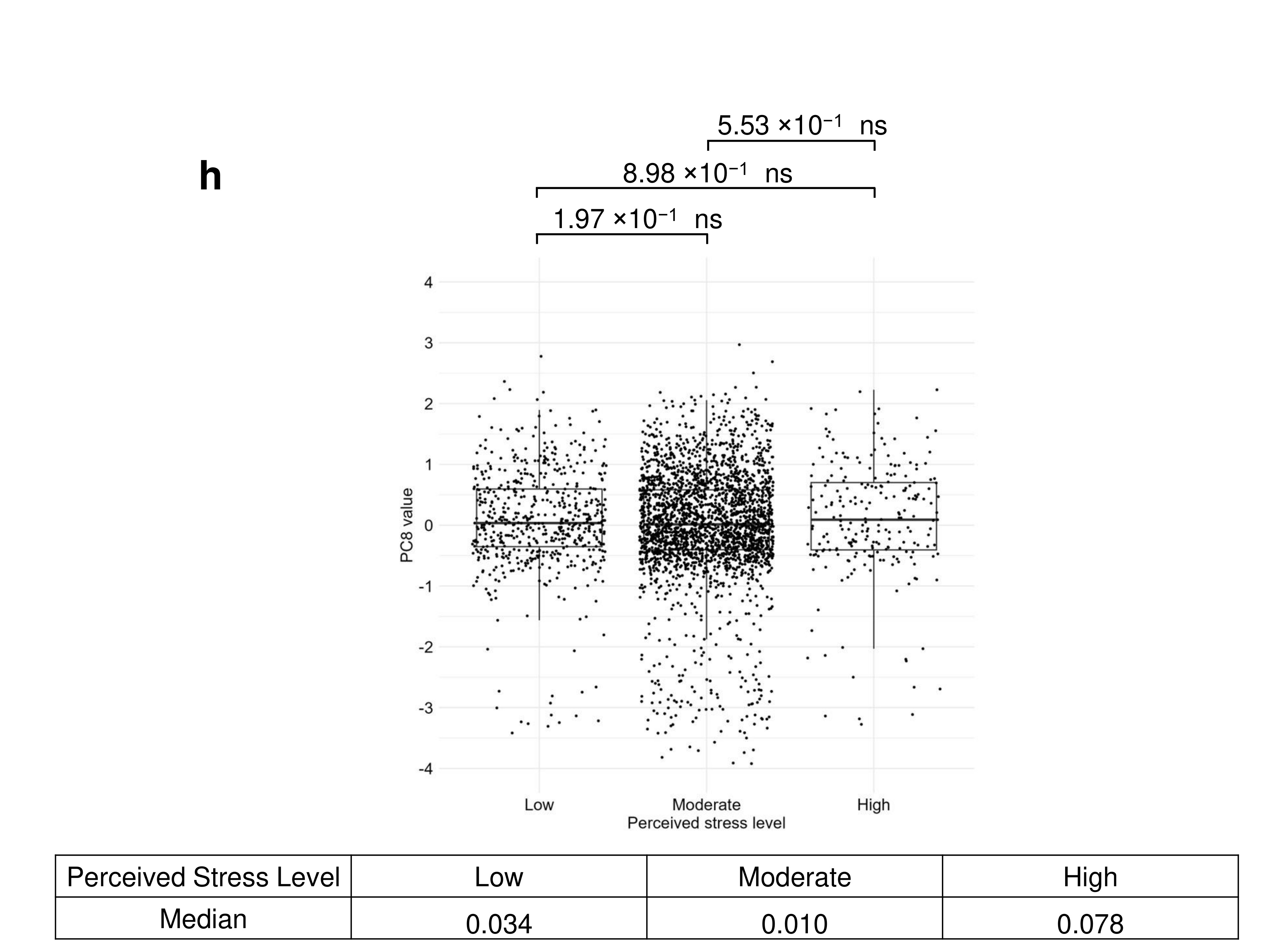

Supplement: Supplementary file 42 — Additional file 42. PC8 values stratified by perceived stress level. Two-tailed t-test p-values are computed for each plot. Perceived stress levels are based on self-reported scores from the Perceived Stress Scale (PSS): low stress (0–13), moderate stress (14–26), and high stress (27–40). The median PC values for low stress, moderate stress, and high stress levels are displayed in each plot. p-values reported are two-tailed t-test p-values, with * indicating p< 0.05, ** p < 0.01, and *** p < 0.001. p > 0.05 was considered statistically non-significant (ns). [file 40101_2024_383_MOESM42_ESM.png]

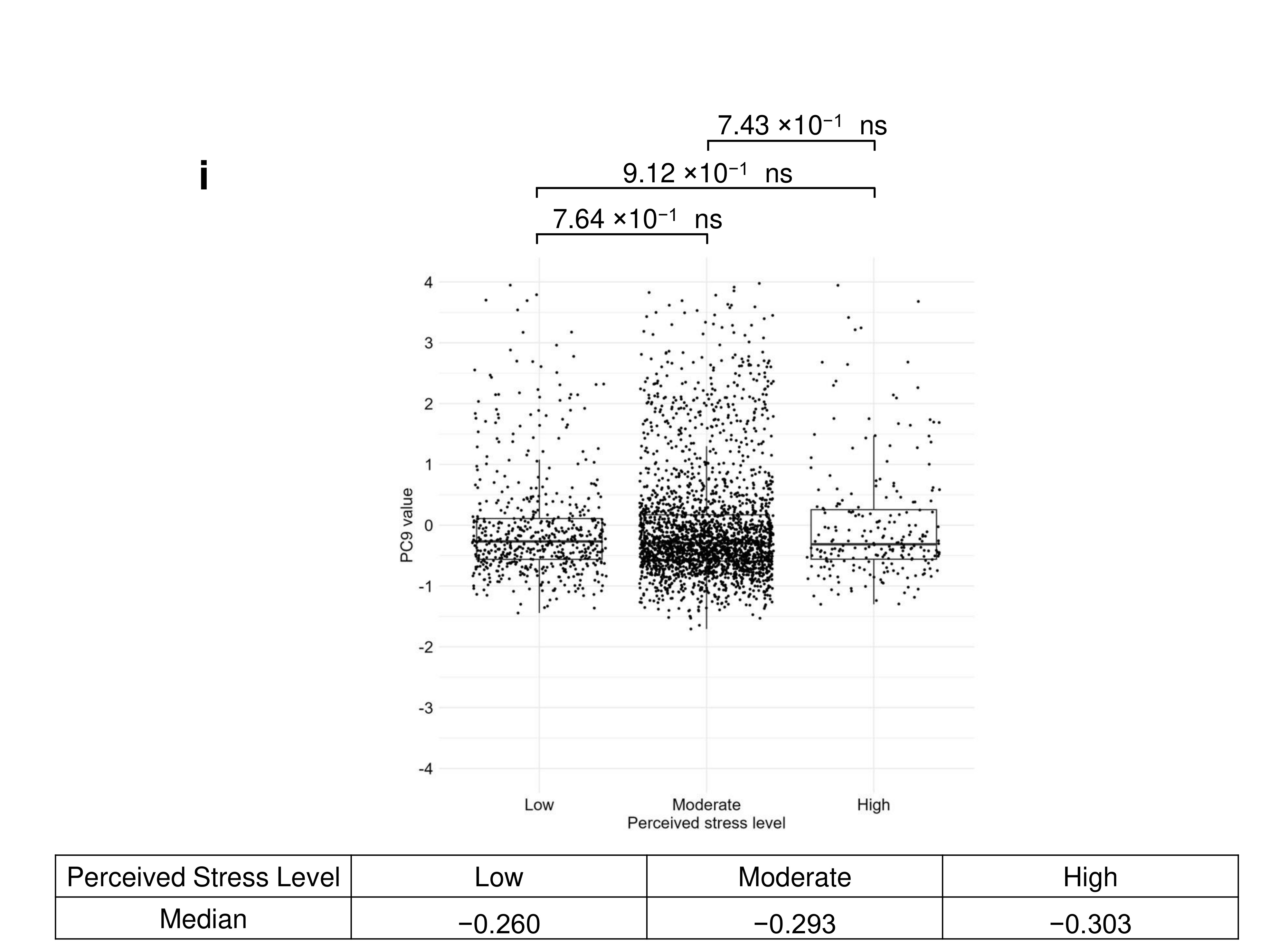

Supplement: Supplementary file 43 — Additional file 43. PC9 values stratified by perceived stress level. Two-tailed t-test p-values are computed for each plot. Perceived stress levels are based on self-reported scores from the Perceived Stress Scale (PSS): low stress (0–13), moderate stress (14–26), and high stress (27–40). The median PC values for low stress, moderate stress, and high stress levels are displayed in each plot. p-values reported are two-tailed t-test p-values, with * indicating p< 0.05, ** p < 0.01, and *** p < 0.001. p > 0.05 was considered statistically non-significant (ns). [file 40101_2024_383_MOESM43_ESM.png]

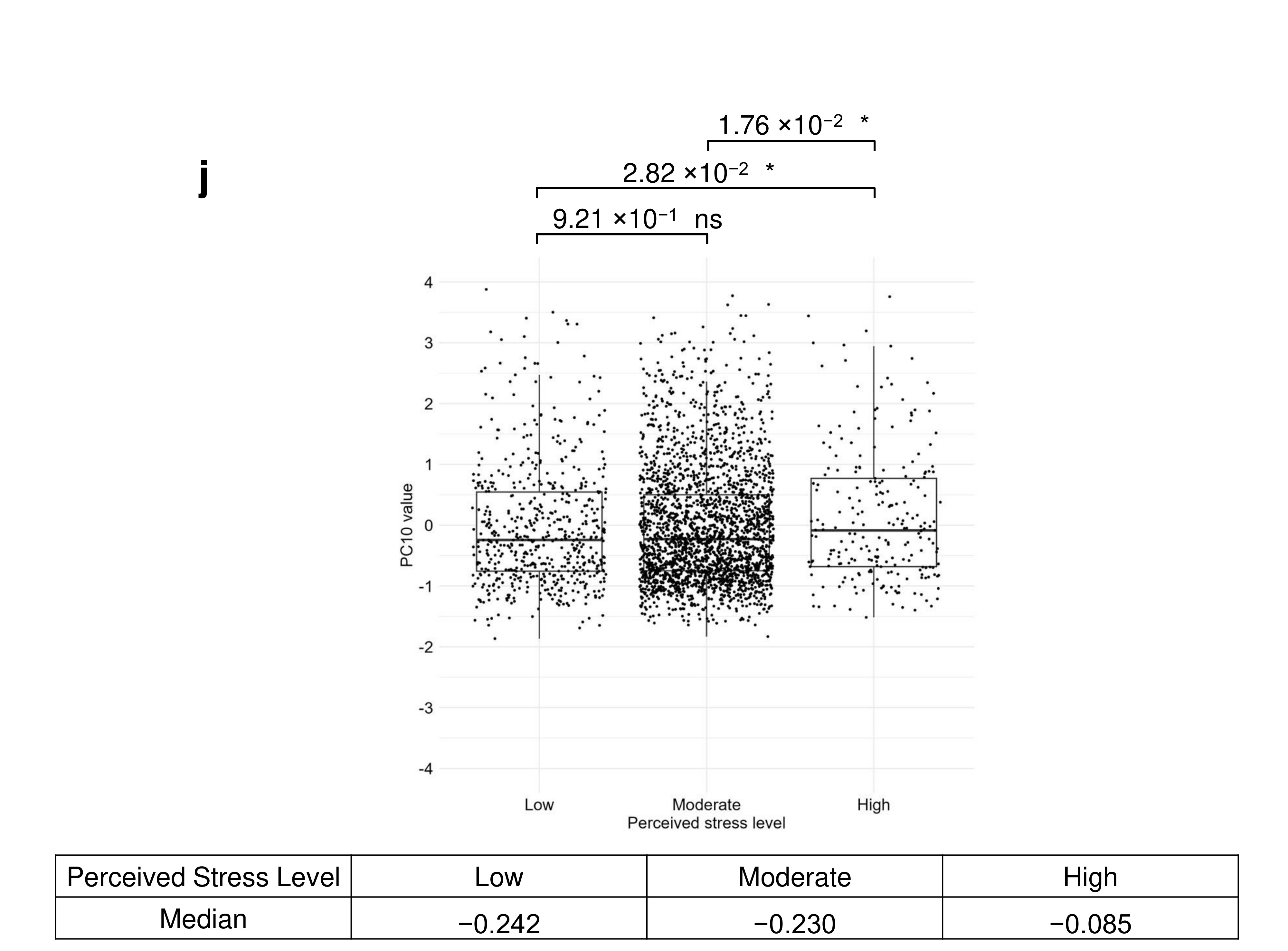

Supplement: Supplementary file 44 — Additional file 44. PC10 values stratified by perceived stress level. Two-tailed t-test p-values are computed for each plot. Perceived stress levels are based on self-reported scores from the Perceived Stress Scale (PSS): low stress (0–13), moderate stress (14–26), and high stress (27–40). The median PC values for low stress, moderate stress, and high stress levels are displayed in each plot. p-values reported are two-tailed t-test p-values, with * indicating p< 0.05, ** p < 0.01, and *** p < 0.001. p > 0.05 was considered statistically non-significant (ns). [file 40101_2024_383_MOESM44_ESM.png]
